# Design, Synthesis, Antiviral Evaluation, and SAR Studies of New 1-(Phenylsulfonyl)-1*H*-Pyrazol−4-yl-Methylaniline Derivatives

**DOI:** 10.3389/fchem.2019.00214

**Published:** 2019-04-09

**Authors:** Nicoletta Desideri, Rossella Fioravanti, Luca Proietti Monaco, Elena Maria Atzori, Antonio Carta, Ilenia Delogu, Gabriella Collu, Roberta Loddo

**Affiliations:** ^1^Dipartimento di Chimica e Tecnologie del Farmaco, Università “La Sapienza”, Rome, Italy; ^2^Dipartimento di Chimica e Farmacia, Università di Sassari, Sassari, Italy; ^3^Dipartimento di Scienze Biomediche, Università di Cagliari, Cagliari, Italy

**Keywords:** 1-(phenylsulfonyl)-1H-pyrazole derivatives, antiviral activity, anti-Flavivirus activity, BVDV, RSV

## Abstract

A series of *N*-((3-phenyl-1-(phenylsulfonyl)-1*H*-pyrazol-4-yl)methyl)anilines **7a-p** and **8a-l**, structurally related to previously synthesized and tested (*N*-(1,3-diphenyl-1*H*-pyrazol-4-yl)methyl)anilines (**1a-v**), were designed and synthesized. The new derivatives were evaluated in cell-based assays for their cytotoxicity and antiviral activity against a large panel of RNA and DNA viruses of public health significance. Generally, the tested compounds did not display cytotoxicity toward the cell lines used. The majority of derivatives **7a**-**p** were able to interfered with YFV and RSV replication in the micromolar range showing a marked improvement in potency and selectivity with respect to the reference inhibitors 6-azauridine and ribavirin, respectively. The introduction of a *p*-methoxy substituent on the phenylsulfonyl group (compounds **8a-l**) completely abolished the anti-RSV activity and reduced or eliminated the potency against YFV. On the contrary, several *p*-methoxy analogs were able to interfere with BVDV replication with a comparable (**8b, 8c, 8g**, and **8k**) or better (**8a** and **8f**) potency than the reference inhibitor, ribavirin. Compound **7e**, selected for time of addition experiments on BHK-21 cell cultures infected with YFV, achieved the highest reduction of virus titer when added 2 h post infection and maintained up to 4 h post infection.

## Introduction

The *Flaviviridae* family comprises single-stranded, positive-sense RNA viruses (ssRNA+) that are currently classified into four genera: Flavivirus, Hepacivirus, Pegivirus, and Pestivirus (Simmonds et al., 2017). *Flaviviridae* viruses are responsible for severe human and animal infectious diseases worldwide (Holbrook, 2017; Evans et al., 2018; Ray and Ray, 2019).

The genera Flavivirus and Hepacivirus include several human pathogenic viruses of global medical importance. Within the approximately 70 species of the genus Flavivirus, Yellow Fever Virus (YFV), West-Nile Virus (WNV), Dengue Virus (DENV), Japanese Encephalitis Virus (JEV), Tick-Borne Encephalitis Virus (TBEV), and Zika virus (ZIKV) are arthropod-borne emerging or reemerging pathogens. Flavivirus infections can result in diseases ranging from a “flu-like” illness with fever and general malaise, to sever and potentially fatal disease including hemorrhagic fever, jaundice, seizures, or fatal encephalitis (Sips et al., 2012; Holbrook, 2017). The Hepacivirus genus includes only the Hepatitis C Virus (HCV), a major cause of human hepatitis, worldwide. HCV can cause both acute and chronic infection. Acute HCV infection is usually asymptomatic and often undiagnosed. However, more than 60% of infected person will develop chronic HCV infection with risk of liver cirrhosis or hepatocellular carcinoma. Recently, effective, safer and well-tolerated new anti-HCV drugs have been developed (Li and De Clercq, 2017; Zajac et al., 2019). On the contrary, no effective antiviral therapy is currently available to fight Flavivirus infections. Although human vaccines are available for YFV, JEV, TBEV, and recently DENV, their use is lacking in many areas and outbreaks of Flavivirus infections still occur, with a significant mortality rate (Deen, 2016; Collins and Metz, 2017). Therefore, the development of effective drugs for the treatment of Flavivirus infections is urgently needed.

Viruses belonging to the Pestivirus genus comprise animal pathogens producing heavy economic losses for the livestock industry. The type specie Bovine Viral Diarrhea Virus (BVDV) together with Border Disease Virus (BDV) of sheep and Classical Swine Fever Virus (CSFV) are responsible of a range of clinical manifestations including respiratory problems, chronic wasting disease, immunosuppression leading to a higher susceptibility to secondary infections, abortion and teratogenicity. Despite the morbidity and even mortality caused by Pestivirus infections, no approved antiviral therapy is currently available (Yeşilbag et al., 2017).

As a part of our researches on heterocyclic compounds with antiviral activity (Conti et al., 2014, 2017; Carta et al., 2016; Fioravanti et al., 2017), we recently identified (*N*-(1,3-diphenyl-1*H*-pyrazol-4-yl)methyl)anilines (**1a-v**) as a new class of potent and selective inhibitors of human respiratory syncytial virus (RSV) replication (Fioravanti et al., 2015). Some derivatives were also endowed with a moderate activity against YFV and BVDV representative type members of the Flavivirus genus and the Pestivirus genus, respectively, within the *Flaviviridae* family (Fioravanti et al., 2015). *N*-((1,3-diphenyl-1H-pyrazol-4-yl)methyl)anilines **(1a-d, 1g-i, 1k-o**) able to interfere with YFV and/or BVDV replication were successively tested against two additional significant human pathogens such as DENV-2 and WNV, both belonging to the Flavivirus genus. The new antiviral data showed that the activity of tested compounds **(1a-b, 1d, 1g-i, 1k-o**) extended to DENV-2 replication, while WNV replication was marginally affected by few compounds (**1g**, **1i**, **1n**-**o**) (Table 1). In addition, the compounds exhibited a higher potency against DENV-2 replication than against other selected viruses belonging to the *Flaviviridae* family.

**Table 1:** Cytotoxicity and antiviral activity of *N*-((1,3-diphenyl-1*H*-pyrazol-4-yl)methyl)anilines **(1a-d, 1g-i, 1k-o)** against DENV-2 and WNV viruses.

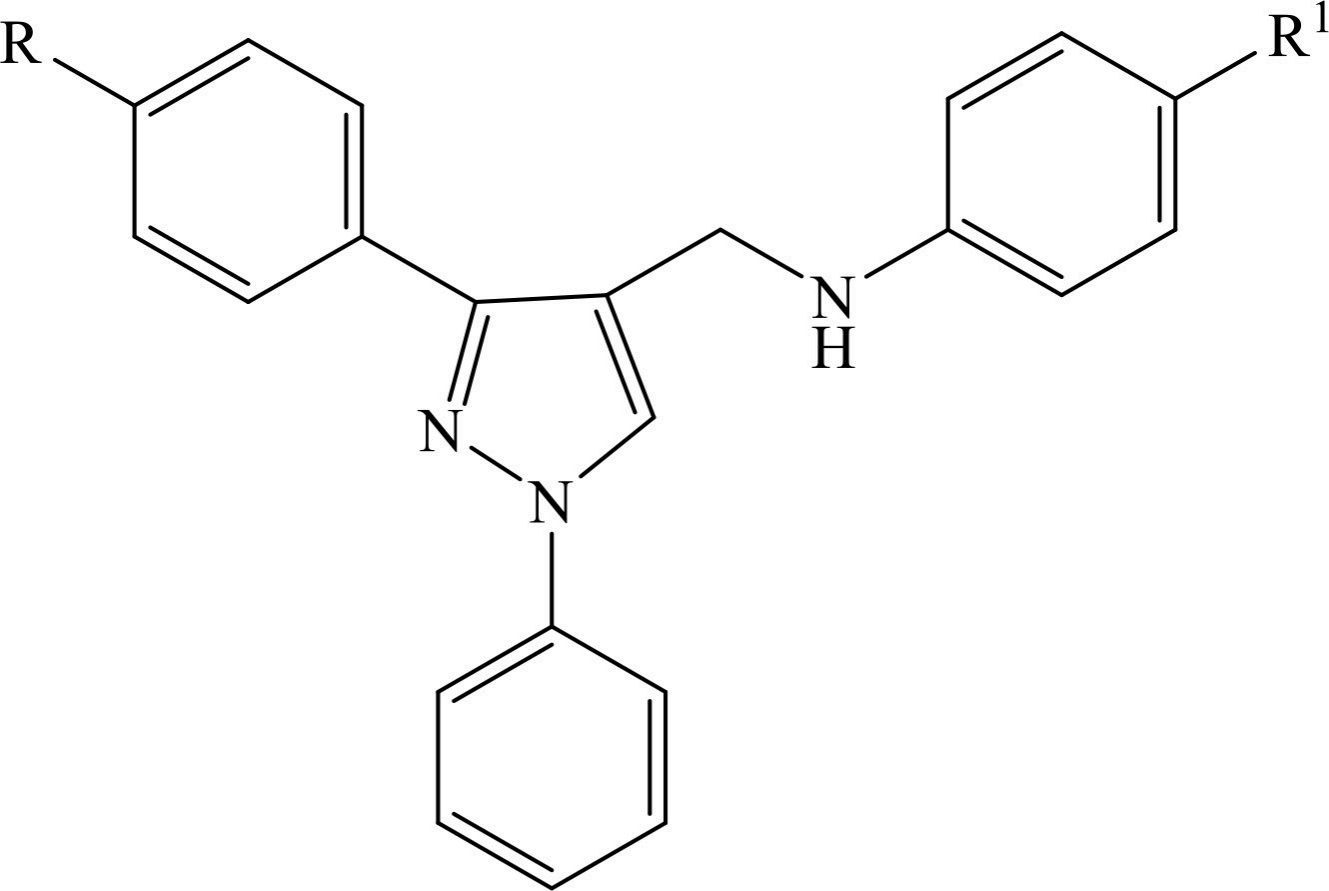							
**Compounds**	**R**	**R**^**1**^	**BHK-21**	**DENV-2**	^**c**^**SI**	**WNV**	^**c**^**SI**
			^**a**^**CC**_**50**_ **(μM)**	^**b**^**EC**_**50**_ **(μM)**	**CC**_**50**_**/EC**_**50**_	^**d**^**EC**_**50**_ **(μM)**	**CC**_**50**_**/EC**_**50**_
**1a**	CH_3_	H	>100	30.0 ± 4.0	>3.3	^e^N.A.	–
**1b**	Br	H	>100	22.0 ± 6.0	>4.5	^e^N.A.	–
**1c**	H	Br	>100	^e^N.A.	–	^e^N.A.	–
**1d**	Cl	Br	>100	11.0 ± 0.4	>9.1	^e^N.A.	–
**1g**	Br	Br	>100	23.0 ± 6.0	>4.3	80.0 ± 8.5	>1.25
**1h**	H	Cl	>100	13.0 ± 5.0	>7.7	^e^N.A.	–
**1i**	Cl	Cl	73.0 ± 1	14.5 ± 3.5	5.0	57.5 ± 0.5	1.3
**1k**	CH_3_	Cl	>100	12.1 ± 3.0	>8.3	^e^N.A.	–
**1l**	Br	Cl	>100	12.9 ± 2.3	>7.8	^e^N.A.	–
**1m**	H	CH_3_	>100	11.2 ± 3.6	>8.9	^e^N.A.	–
**1n**	Cl	CH_3_	>100	11.1 ± 1.6	>9.0	39.0 ± 1.0	>2.6
**1o**	CF_3_	CH_3_	88.0 ± 0.5	14.3 ± 0.7	6.2	59.0 ± 1.1	1.5
**REFERENCE COMPOUND**							
^**f**^**NM 108**			60.0 ± 3	1.2 ± 0.1	50.0	0.7 ± 0.2	85.7

*Data represent mean values for three independent determinations. Variation among duplicate samples was < 15%*.

a *Compound concentration required to reduce the viability of mock-infected BHK (Hamster normal kidney fibroblast) monolayers by 50%, as determined by the MTT method*.

b *Compound concentration required to achieve 50% protection of BHK cells from DENV-2 induced cytopathogenicity, as determined by the MTT method*.

c *Selectivity index (SI) was the ratio between CC_50_ and EC_50_*.

d *Compound concentration required to achieve 50% protection of BHK cells from WNV induced cytopathogenicity, as determined by the MTT method*.

e *No activity (N.A.) up to the highest concentration tested (100 μM)*.

f ***NM108** (2′-C-methylguanosin)*.

**Figure 1:**
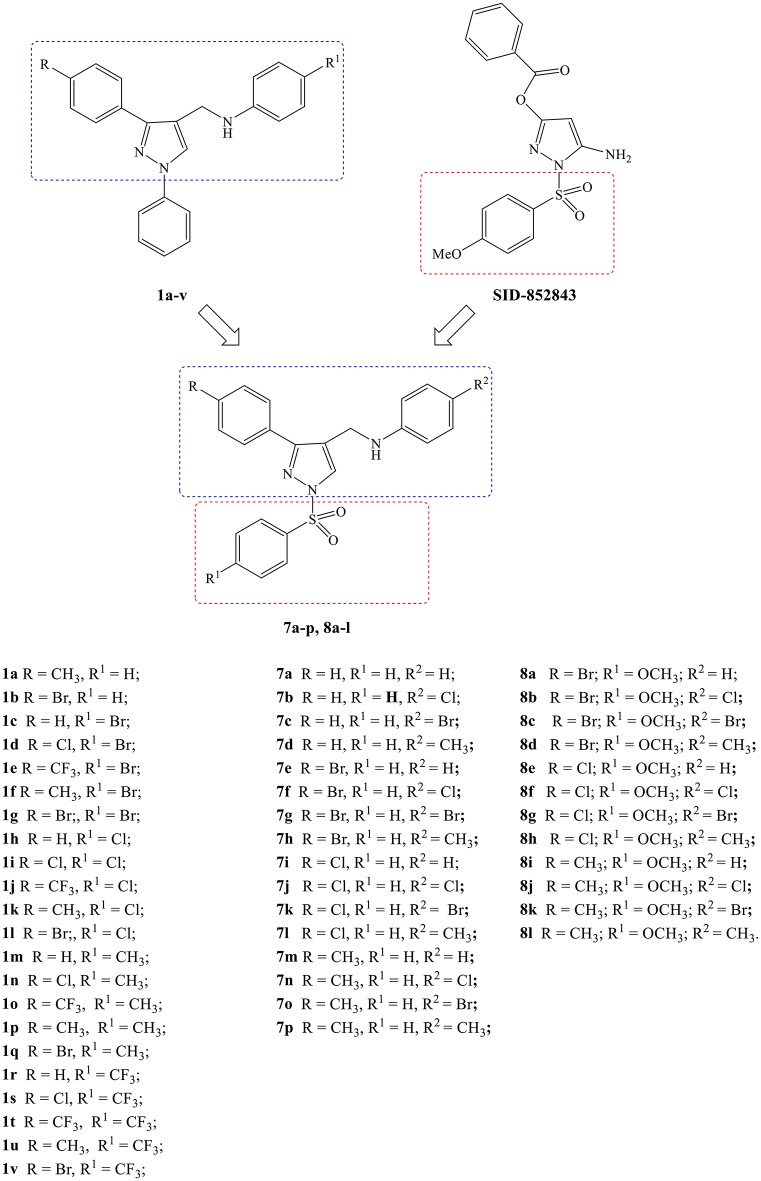
Design of new *N*-((3-phenyl-1-(phenylsulfonyl)-1*H*-pyrazol-4-yl)methyl)anilines (**7a-p**, **8a-l**).

The identification of 5-amino-1-(phenylsulfonyl)-1*H*-pyrazol-3-yl benzoate derivatives (SIDs) as potent but hydrolytically unstable, allosteric inhibitors of WNV NS2B-NS3 proteinase (Johnston et al., 2007; Sidique et al., 2009) suggested to combine the 1-phenylsulfonyl fragment of SIDs with the *N*-((3-phenyl-1*H*-pyrazol-4-yl)methyl)aniline core of compounds **1a-v** in order to identify new promising *Flaviviridae* inhibitors (Figure 1).

In this paper, we report the design and synthesis of novel 1-(phenylsulfonyl)-1*H*-pyrazol-4-yl-methylaniline derivatives (**7a-p** and **8a-l**), and their evaluation in cell-based assays for cytotoxicity and antiviral activity against a large panel of RNA and DNA viruses.

## Results and Discussion

### Chemistry

**Scheme 1:**
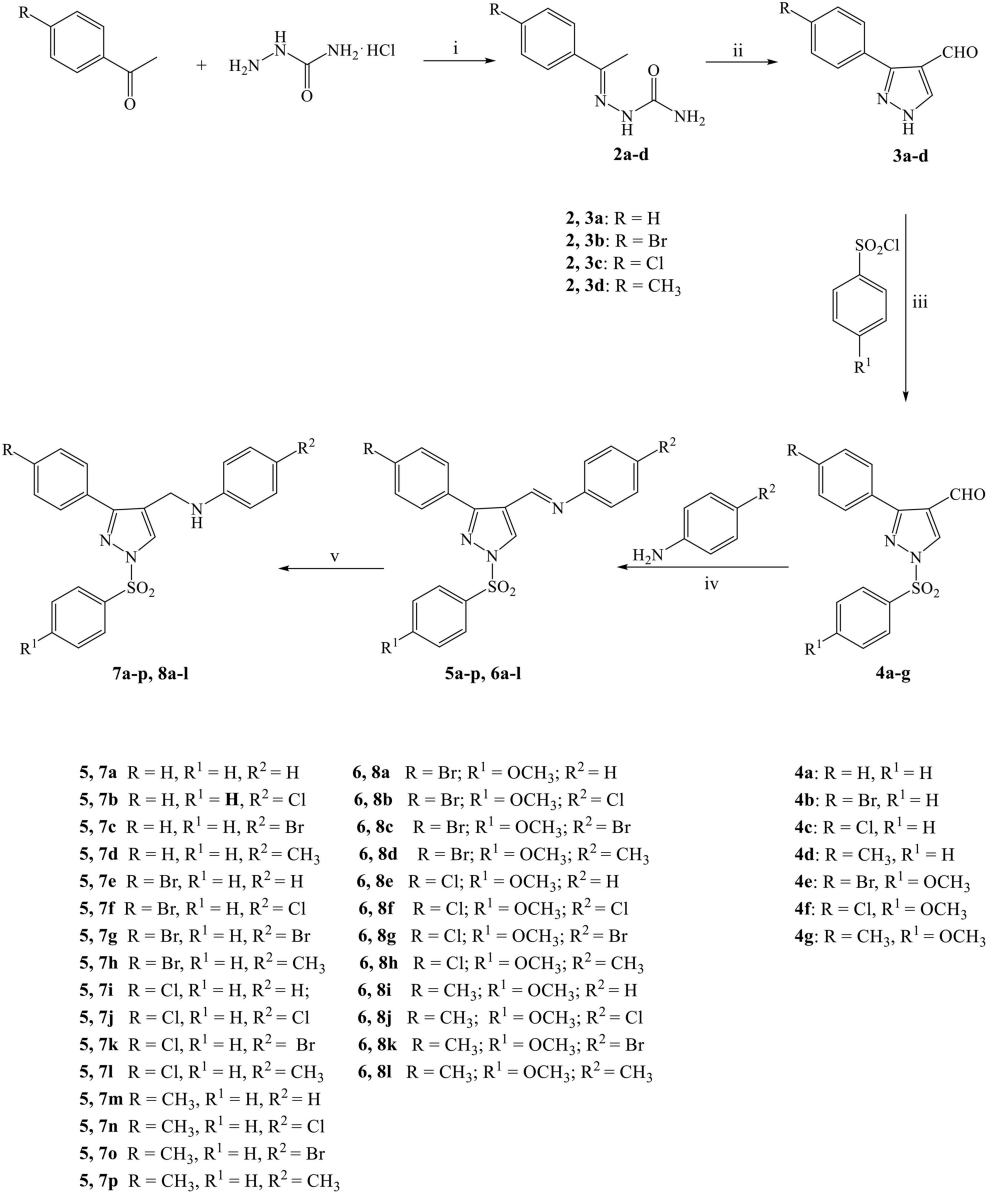
Synthesis of compounds **7a-p** and **8a-l**. Reagents and conditions (i) (1) EtOH, AcONa, r.t. (2) Semicarbazide hydrochloride, water, refluxed, 6 h. (3) r.t., 18 h; (ii) (1) dry DMF, POCl_3_, 0°C, 30′ (2) 65°C, 6 h (3) r.t., 18 h; (iii) dry THF, NaH, r.t., 24 h; (iv) dry EtOH, glacial AcOH, 80–90°C, 6 h; (v) dry THF, NaBH_4_, r.t., 24 h.

As shown in Scheme 1, the compounds **7a-p** and **8a-l** were synthesized in five steps starting from the condensation of the suitable acetophenone with semicarbazide hydrochloride, in the presence of sodium acetate. The obtained semicarbazones **2a-d** were treated with Vilsmeier-Haack reagent (DMF-POCl_3_) to give the corresponding 3-phenyl-1*H*-pyrazole-4-carbaldehydes (**3a-d**) which were converted into the respective 3-phenyl-1-(phenylsulfonyl)-1*H-*pyrazole-4-carbaldehydes (**4a-g**) by treatment with the appropriate benzenesulfonyl chloride in the presence of sodium hydride. The following condensation of the carbaldehydes **4a-g** with properly substituted anilines provided the corresponding Schiff bases **5a-p** and **6a-l** which were directly converted into the desired *N*-((3-phenyl-1-(phenylsulfonyl)-1*H*-pyrazol-4-yl)methyl)anilines (**7a-p** and **8a-l**) by reduction with sodium borohydride.

### Antiviral Tests

All the new synthesized pyrazole derivatives (**7a-p** and **8a-l**) and reference inhibitors were initially tested in cell based assays for their cytotoxicity and antiviral activity against YFV and BVDV, representative of the Flavivirus and the Pestivirus genus, respectively, within the *Flaviviridae* family (Table 2). Their efficacy was also initially evaluated against RSV, a single-stranded, negative RNA virus (ssRNA^−^) belonging to *Paramyxoviridae* family (Table 2). The previously studied *N*-((1,3-diphenyl-1H-pyrazol-4-yl)methyl)anilines and the new derivatives able to inhibit YFV and/or BVDV replication were also evaluated against two additional pathogenic viruses belonging to the Flavivirus genus, DENV-2 and WNV (Tables 1, 2). All the new compounds were further assayed against representative members of a large panel of virus families. Among ssRNA^+^ viruses, a retrovirus (Human Immunodeficiency Virus type-1, HIV-1), and two Picornaviruses (Coxsackie Virus type-5, CVB-5, and Poliovirus type-1, Sabin strain, Sb-1) were also considered. Among ssRNA^−^ viruses, in addition to RSV, a *Rhabdoviridae* family member (Vesicular Stomatitis Virus, VSV) was selected. Among double-stranded RNA (dsRNA) viruses, a reovirus (Reo-1, *Reoviridae*) was included. Finally, among DNA viruses, Herpes Simplex Virus type-1 (HSV-1, *Herpesviridae*), and Vaccinia Virus (VV, *Poxviridae*) were involved (Table 3).

**Table 2:** Cytotoxicity and antiviral activity of *N*-((3-phenyl-1-(phenylsulfonyl)-1*H*-pyrazol-4-yl)methyl)anilines **7a-p** and **8a-l** against ssRNA^+^ (BVDV, YFV, DENV-2, WNV) and ssRNA^−^ (RSV) viruses.

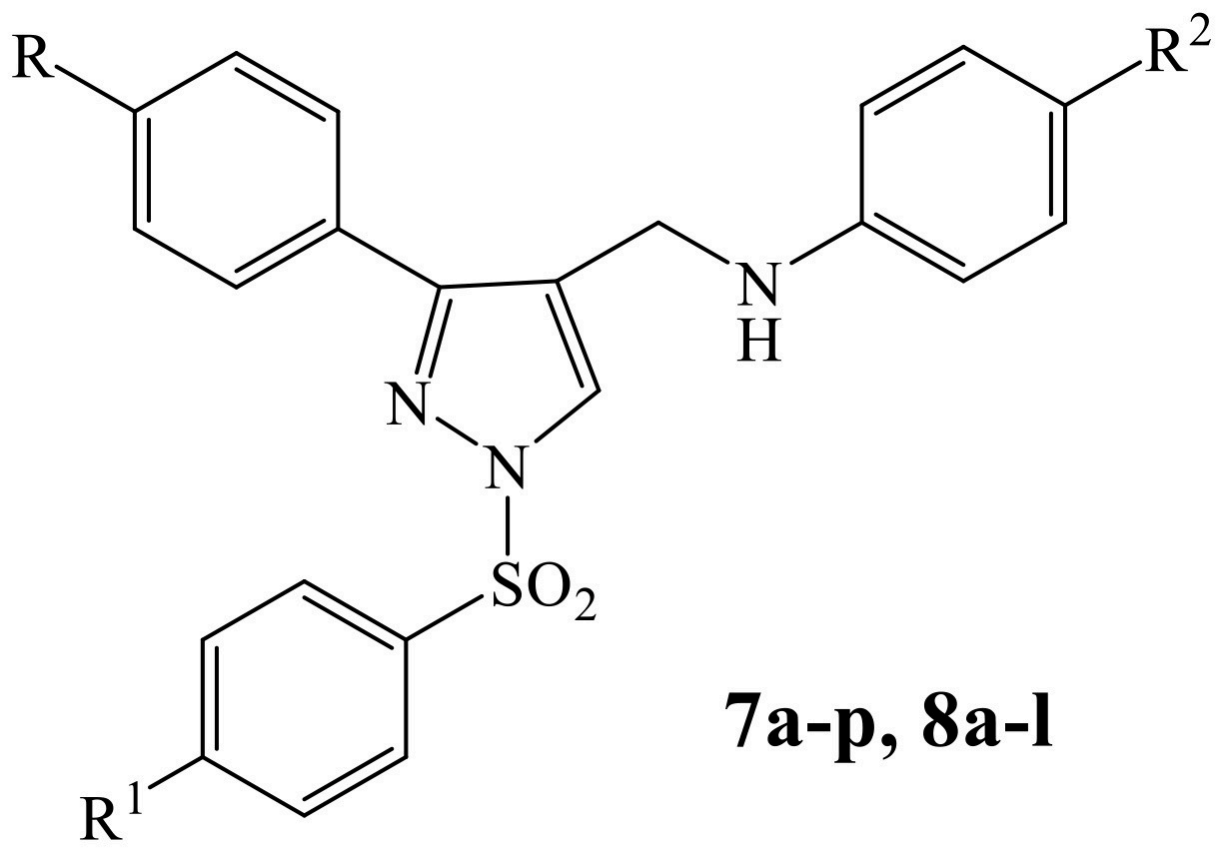													
**Compounds**	**R**	**R**^**1**^	**R**^**2**^	**MDB K**	**BVDV**	^**c**^**SI**	**BHK-21**	**YFV**	^**c**^**SI**	**DENV-2**	**WNV**	**Vero76**	**RSV**	^**c**^**SI**
				^**a**^**CC**_**50**_ **(μM)**	^**b**^**EC**_**50**_ **(μM)**	**CC**_**50**_**/EC**_**50**_	^**d**^**CC**_**50**_ **(μM)**	^**e**^**EC**_**50**_ **(μM)**	**CC**_**50**_**/EC**_**50**_	^**f**^**EC**_**50**_ **(μM)**	^**g**^**EC**_**50**_ **(μM)**	^**h**^**CC**_**50**_ **(μM)**	^**i**^**EC**_**50**_ **(μM)**	**CC**_**50**_**/EC**_**50**_
**7a**	H	H	H	>100	^j^N.A.	–	>100	^j^N.A.	–	–	–	>100	^j^N.A.	–
**7b**	H	H	Cl	>100	^j^N.A.	–	>100	8.1 ± 1.3	>12.3	^j^N.A.	^j^N.A.	10.0 ± 0.1	^j^N.A.	–
**7c**	H	H	Br	>100	^j^N.A.	–	>100	5.9 ± 0.2	>16.9	^j^N.A.	^j^N.A.	3.0 ± 0.01	>3.0	–
**7d**	H	H	CH_3_	>100	^j^N.A.	–	>100	^j^N.A.	–	–	–	≥100	^j^N.A.	–
**7e**	Br	H	H	>100	19.5 ± 2.1	>5.1	>100	3.6 ± 0.8	>27.8	^j^N.A.	^j^N.A.	87.0 ± 1	17.8 ± 1.8	4.9
**7f**	Br	H	Cl	>100	^j^N.A.	–	>100	4.0 ± 0.7	>25.0	^j^N.A.	^j^N.A.	90.0 ± 2	15.3 ± 2.5	5.9
**7g**	Br	H	Br	>100	^j^N.A.	–	>100	6.2 ± 0.5	>16.1	^j^N.A.	^j^N.A.	90.0 ± 1	15.8 ± 2.0	5.7
**7h**	Br	H	CH_3_	>100	^j^N.A.		>100	4.9 ± 0.9	>20.4	^j^N.A.	^j^N.A.	93.0 ± 0.5	12.5 ± 2.5	7.4
**7i**	Cl	H	H	>100	^j^N.A.	–	>100	7.2 ± 0.9	>13.9	^j^N.A.	^j^N.A.	>100	11.8 ± 2.5	>8.5
**7j**	Cl	H	Cl	>100	43.3 ± 1.1	>2.3	>100	4.4 ± 0.4	>22.7	^j^N.A.	^j^N.A.	>100	8.5 ± 0.5	>11.8
**7k**	Cl	H	Br	>100	^j^N.A.	–	>100	5.6 ± 0.6	>17.9	^j^N.A.	^j^N.A.	>100	13.8 ± 0.4	>7.2
**7l**	Cl	H	CH_3_	>100	^j^N.A.	–	>100	6.6 ± 0.6	>15.2	^j^N.A.	^j^N.A.	>100	24.0 ± 1.0	>4.2
**7m**	CH_3_	H	H	>100	^j^N.A.	–	>100	11.5 ± 1.0	>8.7	^j^N.A.	^j^N.A.	>100	12.5 ± 2.1	>8.0
**7n**	CH_3_	H	Cl	>100	^j^N.A.	–	>100	5.1 ± 0.6	>19.6	^j^N.A.	^j^N.A.	>100	13.0 ± 2.8	>7.7
**7o**	CH_3_	H	Br	>100	^j^N.A.	–	>100	3.7 ± 1.3	>27.0	^j^N.A.	^j^N.A.	>100	13.5 ± 0.5	>7.4
**7p**	CH_3_	H	CH_3_	>100	^j^N.A.	–	>100	3.6 ± 0.5	>27.8	^j^N.A.	^j^N.A.	>100	12.0 ± 2.0	>8.3
**8a**	Br	OCH_3_	H	>100	5.6 ± 0.8	>17.9	>100	^j^N.A.	–	^j^N.A.	^j^N.A.	>100	^j^N.A.	–
**8b**	Br	OCH_3_	Cl	>100	20.0 ± 1.4	>5.0	>100	21.6 ± 0.8	>4.6	^j^N.A.	^j^N.A.	>100	^j^N.A.	–
**8c**	Br	OCH_3_	Br	>100	23.0 ± 3.5	>4.4	>100	>100	–	^j^N.A.	^j^N.A.	>100	^j^N.A.	–
**8d**	Br	OCH_3_	CH_3_	>100	^j^N.A.	–	>100	18.3 ± 1.1	>5.5	^j^N.A.	^j^N.A.	100	^j^N.A.	–
**8e**	Cl	OCH_3_	H	>100	^j^N.A.	–	>100	^j^N.A.	–	–	–	>100	^j^N.A.	–
**8f**	Cl	OCH_3_	Cl	>100	7.9 ± 0.8	>12.7	>100	16.0 ± 0.7	>6.25	^j^N.A.	^j^N.A.	>100	^j^N.A.	–
**8g**	Cl	OCH_3_	Br	>100	12.4 ± 0.8	>8.1	>100	18.6 ± 0.8	>5.4	^j^N.A.	^j^N.A.	>100	^j^N.A.	–
**8h**	Cl	OCH_3_	CH_3_	>100	^j^N.A.	–	>100	^j^N.A.	–	–	–	>100	^j^N.A.	–
**8i**	CH_3_	OCH_3_	H	>100	^j^N.A.	–	>100	^j^N.A.	–	–	–	>100	^j^N.A.	–
**8j**	CH_3_	OCH_3_	Cl	>100	^j^N.A.	–	>100	^j^N.A.	–	–	–	>100	^j^N.A.	–
**8k**	CH_3_	OCH_3_	Br	>100	19.5 ± 1.4	>5.1	>100	N.A.	–	^j^N.A.	^j^N.A.	>100	^j^N.A.	–
**8l**	CH_3_	OCH_3_	CH_3_	>100	^j^N.A.	–	>100	N.A.	–	–	–	>100	^j^N.A.	–
**REFERENCE COMPOUNDS**													
**Ribavirin**				55.0 ± 7.0	16.0 ± 2.0	3.4						>100	37.5 ± 2.5	>2.7
**6-Azauridine**							>100	46.0 ± 1.5	>2.2			9.3 ± 1.6	1.3 ± 0.3	7.2
^**k**^**NM108**							60.0 ± 3.0			1.2 ± 0.1	0.7 ± 0.2			

*Data represent mean values for three independent determinations. Variation among duplicate samples was < 15%*.

a *Compound concentration required to reduce the viability of mock-infected MDBK (Bovine normal kidney) cells by 50%, as determined by the MTT method*.

b *Compound concentration required to achieve 50% protection of MDBK cells from the BVDV-induced cytopathogenicity, as determined by the MTT method*.

c *Selectivity index (SI) was the ratio between CC_50_ and EC_50_*.

d *Compound concentration required to reduce the viability of mock-infected BHK (Hamster normal kidney fibroblast) monolayers by 50%, as determined by the MTTmethod*.

e *Compound concentration required to reduce the viability of mock-infected BHK cells from the YFV-induced cytopathogenicity, as determined by the MTT method*.

f *Compound concentration required to achieve 50% protection of BHK cells from DENV-2 induced cytopathogenicity, as determined by the MTT method*.

g *Compound concentration required to achieve 50% protection of BHK cells from WNV induced cytopathogenicity, as determined by the MTT method*.

h *Compound concentration required to reduce the viability of mock-infected VERO76 (monkey normal kidney) monolayers by 50% after contact with the cells for 5 days*.

i *Compound concentration required to reduce the plaque number of RSV (Respiratory Syncytial Virus) by 50% in VERO 76 monolayers*.

j *No activity (N.A.) up to the highest concentration tested (100 μM)*.

k ***NM108** (2′-C-methylguanosin)*.

**Table 3:** Cytotoxicity and antiviral activity of *N*-((3-phenyl-1-(phenylsulfonyl)-1*H*-pyrazol-4-yl)methyl)anilines **7a-p** and **8a-l** against ssRNA^+^ (HIV-1, CVB-5, Sb-1), ssRNA^−^ (VSV), dsRNA (Reo-1) and DNA (VV, HSV-1) viruses.

**Compounds**	**R**	**R^**1**^**	**R^**2**^**	**MT-4**	**HIV-1**	**BHK-21**	**Reo-1**	**Vero76**	**CVB-5**	**Sb-1**	**VV**	**HSV-1**	**VSV**
				**^a^CC_**50**_** **(μM)**	**^b^EC_**50**_** **(μM)**	**^c^CC_**50**_** **(μM)**	**^d^EC_**50**_** **(μM)**	**^e^CC_**50**_** **(μM)**			**EC**_****50****_ ^**f**^ **(μM)**						
**7a**	H	H	H	>100	^g^N.A.	>100	^g^N.A.	>100	^g^N.A.	^g^N.A.	^g^N.A.	^g^N.A.	^g^N.A.
**7b**	H	H	Cl	>100	^g^N.A.	>100	^g^N.A.	80.0 ± 1	20.0 ± 5.0	>80.0	>80.0	>80.0	>80.0
**7c**	H	H	Br	20	>20	>100	^g^N.A.	85.0 ± 0.5	24.0 ± 11.0	>85.0	>85.0	>85.0	>85.0
**7d**	H	H	CH_3_	>100	^g^N.A.	>100	^g^N.A.	≥100	^g^N.A.	^g^N.A.	^g^N.A.	^g^N.A.	^g^N.A.
**7e**	Br	H	H	>100	20.0 ± 2.4	>100	^g^N.A.	87.0 ± 2	>87.0	>87.0	>87.0	>87.0	>87.0
**7f**	Br	H	Cl	>100	^g^N.A.	>100	^g^N.A.	90.0 ± 1	>90.0	>90.0	>90.0	>90.0	>90.0
**7g**	Br	H	Br	>100	^g^N.A.	>100	^g^N.A.	90.0 ± 1	>90.0	>90.0	>90.0	>90.0	>90.0
**7h**	Br	H	CH_3_	>100	^g^N.A.	>100	^g^N.A.	93.0 ± 0.5	>93.0	>93.0	>93.0	>93.0	>93.0
**7i**	Cl	H	H	>100	^g^N.A.	>100	^g^N.A.	^g^N.A.	^g^N.A.	^g^N.A.	^g^N.A.	^g^N.A.	^g^N.A.
**7j**	Cl	H	Cl	>100	^g^N.A.	>100	^g^N.A.	^g^N.A.	^g^N.A.	^g^N.A.	^g^N.A.	^g^N.A.	^g^N.A.
**7k**	Cl	H	Br	>100	^g^N.A.	>100	^g^N.A.	^g^N.A.	^g^N.A.	^g^N.A.	^g^N.A.	^g^N.A.	^g^N.A.
**7l**	Cl	H	CH_3_	>100	^g^N.A.	>100	^g^N.A.	^g^N.A.	^g^N.A.	^g^N.A.	^g^N.A.	^g^N.A.	^g^N.A.
**7m**	CH_3_	H	H	>100	^g^N.A.	>100	^g^N.A.	^g^N.A.	^g^N.A.	^g^N.A.	^g^N.A.	^g^N.A.	^g^N.A.
**7n**	CH_3_	H	Cl	>100	^g^N.A.	>100	^g^N.A.	^g^N.A.	^g^N.A.	^g^N.A.	^g^N.A.	^g^N.A.	^g^N.A.
**7o**	CH_3_	H	Br	>100	^g^N.A.	>100	^g^N.A.	^g^N.A.	^g^N.A.	^g^N.A.	^g^N.A.	^g^N.A.	^g^N.A.
**7p**	CH_3_	H	CH_3_	>100	^g^N.A.	>100	^g^N.A.	^g^N.A.	^g^N.A.	^g^N.A.	^g^N.A.	^g^N.A.	^g^N.A.
**8a**	Br	OCH_3_	H	>100	^g^N.A.	>100	^g^N.A.	^g^N.A.	^g^N.A.	^g^N.A.	^g^N.A.	^g^N.A.	^g^N.A.
**8b**	Br	OCH_3_	Cl	>100	^g^N.A.	>100	^g^N.A.	^g^N.A.	^g^N.A.	^g^N.A.	^g^N.A.	^g^N.A.	^g^N.A.
**8c**	Br	OCH_3_	Br	>100	^g^N.A.	>100	^g^N.A.	^g^N.A.	^g^N.A.	^g^N.A.	^g^N.A.	^g^N.A.	^g^N.A.
**8d**	Br	OCH_3_	CH_3_	>100	^g^N.A.	>100	^g^N.A.	^g^N.A.	^g^N.A.	^g^N.A.	^g^N.A.	^g^N.A.	^g^N.A.
**8e**	Cl	OCH_3_	H	>100	^g^N.A.	>100	^g^N.A.	^g^N.A.	^g^N.A.	^g^N.A.	^g^N.A.	^g^N.A.	^g^N.A.
**8f**	Cl	OCH_3_	Cl	>100	^g^N.A.	>100	^g^N.A.	^g^N.A.	^g^N.A.	^g^N.A.	^g^N.A.	^g^N.A.	^g^N.A.
**8g**	Cl	OCH_3_	Br	>100	^g^N.A.	>100	^g^N.A.	^g^N.A.	^g^N.A.	^g^N.A.	^g^N.A.	^g^N.A.	^g^N.A.
**8h**	Cl	OCH_3_	CH_3_	>100	^g^N.A.	>100	^g^N.A.	^g^N.A.	^g^N.A.	^g^N.A.	^g^N.A.	^g^N.A.	^g^N.A.
**8i**	CH_3_	OCH_3_	H	>100	^g^N.A.	>100	^g^N.A.	^g^N.A.	^g^N.A.	^g^N.A.	^g^N.A.	^g^N.A.	^g^N.A.
**8j**	CH_3_	OCH_3_	Cl	>100	^g^N.A.	>100	^g^N.A.	^g^N.A.	^g^N.A.	^g^N.A.	^g^N.A.	^g^N.A.	^g^N.A.
**8k**	CH_3_	OCH_3_	Br	>100	^g^N.A.	>100	^g^N.A.	^g^N.A.	^g^N.A.	^g^N.A.	^g^N.A.	^g^N.A.	^g^N.A.
**8l**	CH_3_	OCH_3_	CH_3_	>100	^g^N.A.	>100	^g^N.A.	^g^N.A.	^g^N.A.	^g^N.A.	^g^N.A.	^g^N.A.	^g^N.A.
**REFERENCE COMPOUNDS**													
^**h**^**EFV**				40 ± 2.5	0.002 ± 0.001								
^**i**^**ACG**								>100				2.9 ± 0.1	
**Pleconaril**								70.0 ± 1	0.0025 ± 0.0005	2.0 ± 0.1			
^**j**^**M 5255**								20.0 ± 0.3			2.0 ± 0.1		
^**k**^**NM107**						>100	7.5 ± 1.5						

*Data represent mean values for three independent determinations. Variation among duplicate samples was < 15%*.

a *Compound concentration required to reduce the viability of mock-infected MT-4 (cd4+ Human T-cells containing an integrated HTL V-1 genome) cells by 50%, as determined by the MTT method*.

b *Compound concentration required to achieve 50% protection of MT-4 cells from the HIV-1 induced cytioathigenicity, as determined by the MTT method*.

c *Compound concentration required to reduce the viability of mock-infected BHK (hamster normal kidney fibroblast) monolayers by 50%, as determined by the MTT method*.

d *Compound concentration required to achieve 50% protection of BHK cells (kideney fibroblast) from the Reo (Reovirus 1), induced cytopathogenicity, as determined by the MTT method*.

e *Compound concentration required to reduce the viability of mock-infected VERO76 (monkey normal kidney) monolayers by 50% after contact with the cells for 2 days for Sb-1 and VSV, and 3 days for CVB-5, VV and HSV-1*.

f *Compound concentration required to reduce the plaque number of CVB-5 (Coxsackievirus B5), Sb-1 (Poliovirus 1), VV (Vaccina virus), HSV-1 (Herpesvirus 1) and VSV (Vescicular Stomatitis Virus) by 50% in VERO76 monolayers*.

g *No activity (N.A.) up to the highest concentration tested (100 μM)*.

h ***EFV** (Efavirenz)*.

i ***ACG** (Acyclovir)*.

j ***M 5255** (Mycophenolic acid)*.

k ***NM107** (2′-C-methylcitidine)*.

Results reported in Tables 2, 3 displayed that the new N-((3-phenyl-1-(phenylsulfonyl)-1H-pyrazol-4-yl)methyl)anilines **7a-p** and **8a-l** exhibited no cytotoxicity against cell lines (MDBK, BHK-21, Vero76 and MT-4) used to support replication of selected viruses. The only exceptions were represented by the halo derivatives **7b** and **7c** that showed a significant cytotoxicity against Vero cells after incubation for 5 days, time necessary to detect RSV-induced cytopathogenicity (CC_50_ = 10.0 and 3.0 μM, respectively) (Table 2). Their cytotoxicity decreases substantially when the CC_50_ values were detected after 2 or 3 days of contact with Vero cells, time appropriate to detect Sb-1, VSV, CVB-5, VV and HSV-1 induced cytopathogenicity (CC_50_ = 80.0 μM and 85.0 μM, respectively) (Table 3).

The majority of new *N*-((3-phenyl-1-(phenylsulfonyl)-1*H*-pyrazol-4-yl)methyl)anilines **7e**-**p** exhibited a better activity than the parent compounds **1a-v** (Fioravanti et al., 2015) against YFV (EC_50_ ranging from 3.6 to 11.5 μM) coupled with the absence of cytotoxicity for BHK-21 cell line up to the highest concentration tested (100 μM), resulting in compounds with high selectivity (SI ranging from >27.8 to >8.7). Moreover, all these derivatives showed better activity and selectivity than the reference inhibitor, 6-azauridine (EC_50_ = 46.0 μM, SI > 2.2) (Table 2). Concerning the anti-YFV activity of the unsubstituted analogs in R and R^1^ (**7a-d**), the introduction in R^2^ of an electron-withdrawing group such as a chlorine (**7b**) or a bromine (**7c**) atom, is necessary for the antiviral activity.

Moreover, despite the presence of a *p*-methoxy substituent on the phenylsulfonyl group was required for a potent anti-flavivirus activity in SID compounds (Johnston et al., 2007; Sidique et al., 2009), the new *p*-methoxy phenylsulfonyl analogs **8a-l** were totally inactive or less potent YFV inhibitors than the corresponding unsubstituted compounds **7e-p**. Conversely, the introduction of a *p*-methoxy substituent generally converted the inactive or poor effective unsubstituted analogs **7e-p** in the more potent anti-BVDV agents **8a-l**. In particular, **8a** was the best anti-BVDV compound among the new derivatives (EC_50_ = 5.6 μM, SI > 17.9) showing almost a 3-fold improvement in potency and a 5-fold improvement in selectivity with respect to the reference inhibitor, ribavirin (EC_50_ = 16.0 μM, SI = 3.4) (Table 2).

Antiviral activities against DENV-2 and WNV of 1-phenyl and 1-phenylsulfonyl analogs were reported in Tables 1, 2, respectively. Results revealed that also the DENV replication was affected by several 1-phenyl derivatives (**1a-b**, **1d**, **1g-i**, **1k-o**), whereas only few compounds **(1g**, **1i**, **1n-o**) showed a modest activity against WNV (Table 1). Surprisingly, the replacement of the phenyl ring at N1 position with a phenylsulfonyl group completely abolished the activity against DENV-2 and WNV replication (Table 2).

Similarly to the N-phenyl derivatives **1a-v**, N-phenylsulfonyl analogs **7e-p** exhibited anti-RSV activity in the micromolar concentration (EC_50_ ranging from 8.5 to 24.0 μM) generally coupled with high selectivity (SI ranging from >11.8 to >4.2). SAR studies indicated that the *para* substitution at the 3-phenyl ring is necessary for RSV inhibitory activity. In fact, compounds **7a-d** were devoid of efficacy against RSV up to the highest concentration tested. On the contrary, the introduction of the *p*-methoxy substituent on the phenylsulfonyl moiety (**8a-l**) abolished the activity (Table 2). Moreover, all the derivatives **7e**-**p** able to interfere with RSV replication showed better activity and selectivity than the reference inhibitor ribavirin, a broad-spectrum antiviral agent licensed for the treatment of RSV infection (Wu et al., 2003).

When tested against HIV-1, Reo-1, CVB-5, Sb-1, VV, HSV-1, and VSV, the compounds were devoid of antiviral activity up to the highest concentration tested, with the exceptions of analogs **6b** and **6c** that showed a moderate activity against CVB-5 (EC_50_ = 20.0 and 24.0 μM, respectively) and **6e** that affected the HIV-1 replication (EC_50_ = 20.0 μM) (Table 3).

### Time of Addition Studies

**Figure 2:**
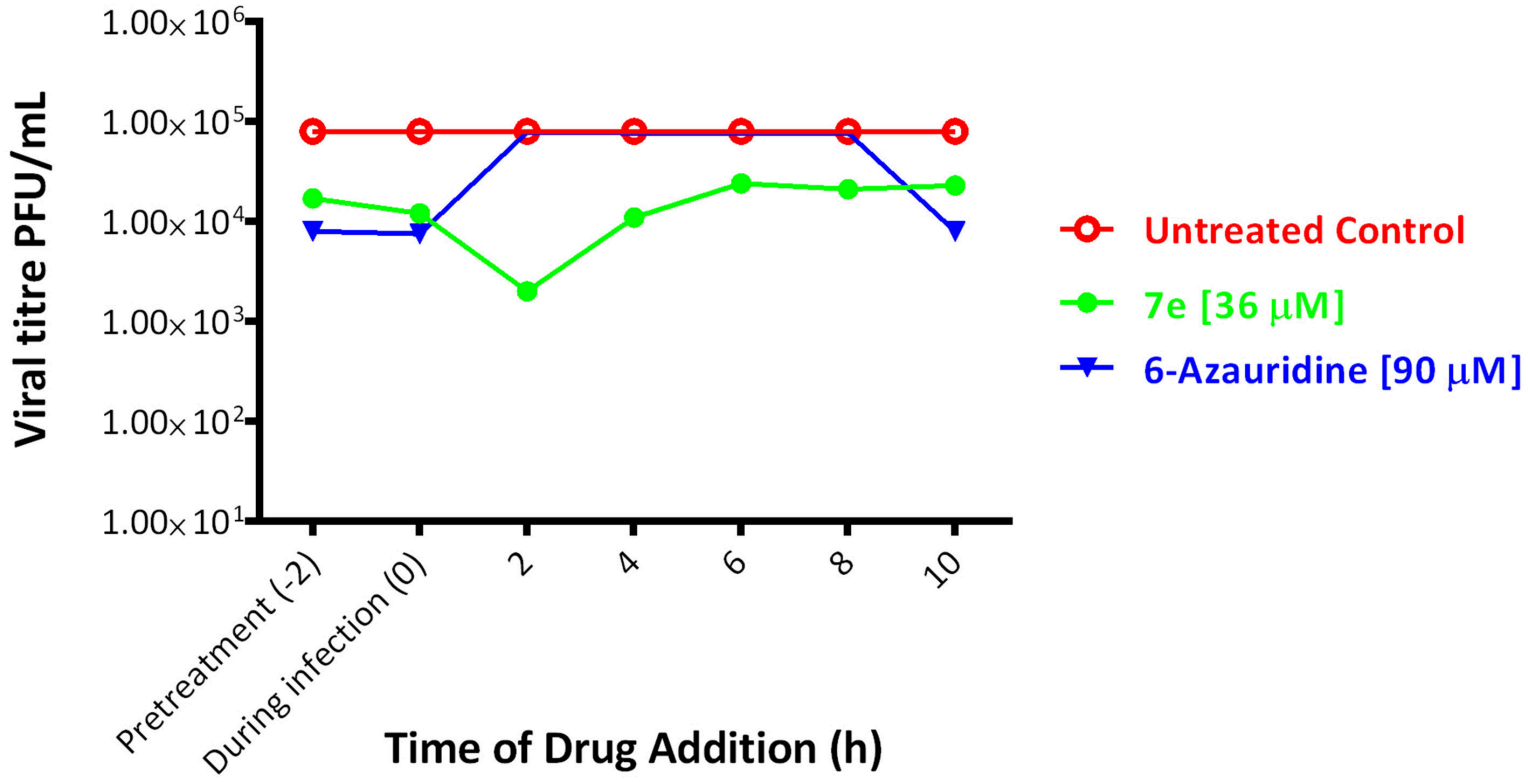
Effect of time of (drug)-addition on anti-YFV activity of derivative **7e** [36 μM] (green). The same test was performed using the reference compound 6-Azauridine [90 μM] (blue) for comparison. In red we observe the untreated control. Mean results of two different experiments, each performed in duplicate; SD < 10%.

Due to its potency against YFV (EC_50_ = 3.6 μM) and low cytotoxicity against BHK-21 cell cultures (CC_50_ > 100 μM), compound **7e** were selected for time of addition studies. In order to determine the possible step(s) in YFV replication cycle inhibited by the derivative **7e**, time of addition experiments were performed on infected BHK-21 cells under a YFV single cycle conditions. 6-Azauridine, a nucleoside analog inhibitor of the orotidine monophosphate (OMP) decarboxylase, was used as reference inhibitor. This enzyme is essential in the biosynthesis of pyrimidine nucleotides, as it converts the OMP in uridine monophosphate (UMP) (Neyts et al., 1996). In time of addition experiments, the selected pyrazole derivative **7e** (at the concentration of 36 μM = 10 x EC_50_) or the reference inhibitor 6-azauridine (at the concentration of 90 μM = 2 × EC_50_) were added to BHK-21 cell cultures for 2 h before the infection with YFV (pre-treatment = time −2), during the 2 h of infection (during infection = time 0) or every 2 h post infection (p.i.), from time 0 to 10 h p.i., and removed after 2 h of contact (0–2, 2–4, 4–6, 6–8, 8–10). Data represented in Figure 2 showed that both pyrazole **7e** and 6-azauridine reduced the YFV titer when present in the pre-treatment or during the 2 h of infection. However, 6-azauridine lost its effectiveness when added after the infection, while **7e** retained its inhibitory activity when added at any time p.i. In particular, the highest reduction of virus yield was observed when **7e** was added 2 h post infection and maintained up to 4 h post infection.

These data suggest that **7e** interferes with different steps of YFV replication starting from the virus binding to host cell membrane but also covering some subsequent phase of the virus replication. However, further studies are necessary to identify the antiviral target(s) of this pyrazole derivative.

## Conclusions

In order to improve the anti-*Flaviviridae* activity of previously studied (*N*-(1,3-diphenyl-1*H*-pyrazol-4-yl)methyl)anilines (**1a-v**), a series of *N*-((3-phenyl-1-(phenylsulfonyl)-1*H*-pyrazol-4-yl)methyl)anilines (**7a-p** and **8a-l**) was designed, synthesized and assayed against a large panel of viruses belonging to *Flaviviridae, Picornaviridae, Paramyxoviridae, Rhabdoviridae, Reoviridae, Retroviridae, Herpesviridae*, and *Poxviridae* families. SAR studies showed that the analogs unsubstituted in R^1^ (**7a**-**p**) were generally potent and high selective YFV inhibitors (EC_50_ ranging from 3.6 to 11.5 μM and SI ranging from >27.8 to >8.7). The analogs **7e**-**p** also interfered with RSV replication in the micromolar concentrations (EC_50_ ranging from 8.5 μM to 24.0 μM) providing an improvement in potency and selectivity with respect to the reference inhibitor ribavirin, the first drug licensed for the treatment of RSV infection. On the contrary, the introduction of a methoxy group in R^1^ resulted in compounds that preferentially affected BVDV replication. Among these derivatives, **8a** was the most potent and selective inhibitor of BVDV replication (EC_50_ = 5.6 μM, SI > 17.9).

In conclusion the results of this work allowed the selection of a new generation of hits for the development of anti-YFV, -BVDV, and -RSV agents.

## Materials and Methods

### Chemistry

Solvents and reagents were purchased from Sigma-Aldrich or Alfa Aesar and were used without further purification. The progress of reactions was routinely checked by thin-layer chromatography (TLC). TLC was performed on silica gel or aluminum oxide fluorescent coated plates (Fluka, DC-Alufolien Kieselgel or aluminum oxide F_254_). Melting points were determined on a Stuar Scientific SMP1 apparatus and are uncorrected. ^1^H NMR and ^13^C NMR spectra were recorded on a Bruker AM-400 spectrometer in CDCl_3_ or DMSO-d_6_, and chemical shifts were reported in ppm (δ) (Supplementary Figures S1–S31).

### General Procedure for the Synthesis of the (*E*)-2-(1-phenylethylidene)hydrazinecarboxamides (2a-d)

A solution of semicarbazide hydrochloride (2.3 mmol) in water (43 ml) was added dropwise to a stirred solution of the appropriate acetophenone (2.2 mmol) and sodium acetate (3 mmol) in ethanol (43 ml) at room temperature. After the addition, the mixture was refluxed for 6 h under magnetic stirring, cooled at room temperature and further stirred for 18 h. Afterwards the ethanol was removed under reduced pressure and the precipitate was collected by filtration and used for the next reaction without further purification.

#### (*E*)-2-(1-Phenylethylidene)hydrazinecarboxamide (2a)

Yield: 83%, m.p. = 196–197°C. ^1^H NMR (DMSO-d_6_, 400 MHz): δ (ppm) 9.40 (s, 1H, NH), 7.84 (d, 2H, H2, H6, *J*_2−3_ = 6.4 Hz), 7.37–7.35 (m, 3H, H3, H4, H5), 6.53 (s, 2H, NH_2_), 2.19 (s, 3H, CH_3_). ^13^C NMR (DMSO-d_6_, 100 MHz): δ (ppm) 157.29, 143.93, 138.17, 128.32, 128.08, 125.84, 13.21.

#### (*E*)-2-(1-(4-Bromophenyl)ethylidene)hydrazinecarboxamide (2b)

Yield: 77%, m.p. = 198–201°C. ^1^H (DMSO-d_6_, 400 MHz): δ (ppm) 9.47 (s, 1H, NH), 7.81 (d, 2H, H2, H6, *J*_2−3_ = 8.0 Hz), 7.54 (d, 2H, H3, H5, *J*_2−3_ = 8.0 Hz), 6.58 (s, 2H, NH_2_), 2.18 (s, 3H, CH_3_). ^13^C (DMSO-d6, 100 MHz): δ (ppm) 157.23, 142.84, 137.44, 130.99, 127.99, 121.74, 13.05.

#### (*E*)- 2-(1(4-Chlorophenyl)ethylidene)hydrazinecarboxamide (2c)

Yield: 74%, m.p. = 198–199°C. ^1^H NMR (DMSO-d_6_, 400 MHz): δ (ppm) 9.45 (s, 1H, NH), 7.88 (d, 2H, H2, H6, *J*_2−3_ = 8.4 Hz), 7.40 (d, 2H, H3, H5, *J*_2−3_ = 8.4 Hz), 6.57 (s, 2H, NH_2_), 2.18 (s, 3H, CH_3_). ^13^C NMR (DMSO-d_6_, 100 MHz): δ (ppm) 157.22, 142.73, 137.06, 133.01, 128.07, 127.70, 13.09.

#### (*E*)-2(1(p-Tolyl)ethylidene)hydrazinecarboxamide (2d)

Yield: 87%, m.p. = 199–200°C (lit. = 200°C). The compound exhibited spectroscopic data identical to those previously reported (Vila et al., 1998).

### General Procedure for the Synthesis of the 3-Phenyl-1*H*-Pyrazol-4-Carbaldeydes (3a-d)

To a solution of the suitable (*E*)-2-(1-phenylethylidene) hydrazinecarboxamides **2a-d** (12 mmol) in dry DMF (9 ml) cooled in ice bath, POCl_3_ (2 ml) was added dropwise. The mixture was stirred in ice bath for 30 min and then heated at 65°C for 6 h. After cooling and stirring at room temperature for 18 h, the mixture was diluted with water and ice, neutralized with NaOH 2N and extracted with ethyl acetate. The organic layer was washed with brine, dried over anhydrous Na_2_SO_4_, filtered and evaporated to dryness. The residue was purified by crystallization from CHCl_3_/n-Hexane. Intermediate **3c** was purified by column chromatography on silica gel eluting with a mixture of AcOEt and n-Hexane 1:1.

#### 3-Phenyl-1*H*-pyrazole-4-carbaldehyde (3a)

Yield: 93%, m.p. = 129–130°C (lit. = 145°C) (Lebedev et al., 2005) from CHCl_3_/n-Hexane. ^1^H NMR (DMSO-d_6_, 400 MHz): δ (ppm) 13.72 (s, 1H, NH), 9.91 (s, 1H, CHO), 8.60 (s, 1H, pyrazole-H5), 7.83 (d, 2H, H2, H6, *J*_2−3_ = 8.0 Hz), 7.56–7.50 (m, 3H, H3, H4, H5). ^13^C NMR (DMSO-d_6_, 100 MHz): δ (ppm) 184.71, 140.01, 133.08, 130.10, 128.76, 127.08, 126.49, 110.48.

#### 3-(4-Bromophenyl)-1*H*-pyrazole-4-carbaldehyde (3b)

Yield 97%, m.p. = 127–128°C (lit. = 163–165°C) (Lebedev et al., 2005) from CHCl_3_/n-Hexane. ^1^H NMR (DMSO-d_6_, 400 MHz): δ (ppm) 13.80 (s, 1H, NH), 9.90 (s, 1H, CHO), 8.49 (s, 1H, pyrazole-H5), 7.83 (d, 2H, H2, H6, *J*_2−3_ = 8.0 Hz), 7.69 (d, 2H, H3, H5, *J*_2−3_ = 8.0 Hz). ^13^C NMR (DMSO-d_6_, 100 MHz): δ (ppm) 184.57, 152.81, 138.86, 138.56, 131.41, 130.39, 122.36, 119.85.

#### 3-(4-Chlorophenyl)-1*H*-pyrazole-4-carbaldehyde (3c)

Yield: 99%, m.p. = 130–131°C (lit. = 142–144°C) (Lebedev et al., 2005) from AcOEt/n-Hexane. ^1^H NMR (DMSO-d_6_, 400 MHz): δ (ppm) 13.70 (s, 1H, NH), 9.91 (s, 1H, CHO), 8.49 (s, 1H, pyrazole-H5), 7.90 (d, 2H, H2, H6, *J*_2−3_ = 8.0 Hz), 7.56 (d, 2H, H3, H5, *J*_2−3_ = 8.0 Hz). ^13^C NMR (DMSO-d_6_, 100 MHz): δ (ppm) 184.64, 140.60, 133.71, 130.18, 128.49, 128.54, 125.39, 119.88.

#### 3-(p-Tolyl)-1*H*-pyrazole-4-carbaldehyde (3d)

Yield: 60%, m.p. = 123–124°C (lit. = 123–125°C) (Lebedev et al., 2005) from AcOEt/n-Hexane. ^1^H NMR (CDCl3, 400 MHz): δ (ppm) 12.58 (s, 1H, NH), 9.94 (s, 1H, CHO), 8.05 (s, 1H, pyrazole-H5), 7.53 (d, 2H, H2, H6, *J* 2−3 = 7.6 Hz), 7.29 (d, 2H, H3, H5, *J* 2−3 = 7.6 Hz), 2.43 (s, 3H, CH3). ^13^C NMR (CDCl3, 100 MHz): δ (ppm) 185.22, 150.08, 140.5, 138.80, 129.87, 128.69, 125.33, 120.03, 21.41.

### General Procedure for the Synthesis of the 3-phenyl-1-(phenylsulfonyl)-1*H*-pyrazol-4-carbaldehydes (4a-g)

NaH (6.8 mmol) was added to a stirred solution of the appropriate 3-phenyl-1*H*-pyrazole-4-carbaldehyde **3a-d** (6.8 mmol) in dry THF (100 ml). The mixture was stirred 30 min at room temperature, then the suitable phenylsulfonyl chloride (9.6 mmol) was added. After stirring for 24 h at room temperature, water was added to the mixture and THF was removed under reduced pressure. The obtained suspension was extracted with ethyl acetate and the organic phase was washed with brine and dried over Na_2_SO_4_ anhydrous, filtered and evaporated to dryness. The residue was purified by crystallization from suitable solvent.

#### 3-Phenyl-1-(phenylsulphonyl)-1*H*-pyrazole-4-carbaldehyde (4a)

Yield: 50%, m.p. = 110–111°C from EtOH/n-Hexane. ^1^H NMR (CDCl_3_, 400 MHz): δ (ppm) 9.92 (s, 1H, CHO), 8.71 (s, 1H, pyrazole-H5), 8.11 (d, 2H, H2, H6, *J*_2−3_ = 8.0 Hz), 7.71–7.69 (m, 3H, H2′, H6′, H4), 7.59 (t, 2H, H3, H5, *J*_2−3_ = 8.0 Hz), 7.46–7.44 (m, 3H, H3′, H4′, H5′). ^13^C NMR (CDCl_3_, 100 MHz): δ (ppm) 184.38, 156.81, 140.93, 136.16, 135.99, 135.34, 130.07, 129.98, 129.68, 129.07, 128.70, 122.66.

#### 3-(4-Bromophenyl)-1-(phenylsulfonyl)-1*H*-pyrazole-4-carbaldehyde (4b)

Yield: 85%, m.p. = 121–122°C from AcOEt/Pet.Et. ^1^H NMR (CDCl_3_, 400 MHz): δ (ppm) 9.97 (s, 1H, CHO), 8.71 (s, 1H, pyrazole-H5), 8.11 (d, 2H, H2, H6, *J*_2−3_ = 7.6 Hz), 7.25 (t, 1H, H4, *J*_3−−4_ = 7.6 Hz), 7.66–7.56 (m, 6H, H3, H5, H2′, H6′, H3′, H5′). ^13^C NMR (CDCl_3_, 100 MHz): δ (ppm) 183.63, 155.33, 137.24, 135.84, 135.45, 131.84, 130.58, 129.73, 129.02, 128.73, 124.56, 122.61.

#### 3-(4-Clorophenyl)-1-(phenylsulphonyl)-1*H*-pyrazole-4-carbaldehyde (4c)

Yield: 60%, m.p. = 121–122°C from EtOH/n-Hexane. ^1^H NMR (DMSO-d_6_, 400 MHz): δ (ppm) 9.95 (s, 1H, CHO), 9.41 (s, 1H, pyrazole-H5), 8.15 (d, 2H, H2, H6, *J*_2−3_ = 7.6 Hz), 7.87 (t, 1H, H4, *J*_3−−4_ = 7.6 Hz), 7.83 (d, 2H, H2′, H6′, *J*${}_{2^{\prime }-3^{\prime }}$ = 8.4 Hz), 7.74 (t, 2H, H3, H5, *J*_2−3_ = *J*_3−−4_ = 7.6 Hz), 7.54 (d, 2H, H3′, H5′, *J*${}_{2^{\prime }-3^{\prime }}$ = 8.4 Hz). ^13^C NMR (DMSO-d_6_, 100 MHz): δ (ppm) 184.65, 153.70, 140.92, 136.02, 135.10, 134.75, 130.52, 130.27, 128.74, 128.55, 128.26, 122.62.

#### 1-(Phenylsulfonyl)-3-(p-tolyl)-1*H*-pyrazole-4-carbaldehyde (4d)

Yield: 90%, m.p. = 126°C da AcOEt/Pet. Et. ^1^H NMR (CDCl_3_, 400 MHz): δ (ppm) 9.98 (s, 1H, CHO), 8.69 (s, 1H, pyrazole-H5), 8.10 (d, 2H, H2, H6, *J*_2−3_ = 8.0 Hz), 7.69 (t, 1H, H4, *J*_3−−4_ = 8.0 Hz), 7.61–7.56 (m, 4H, H2′, H6′, H3, H5), 7.24 (d, 2H, H3′, H5′, *J*_2−3_ = 8.0 Hz), 2,38 (s, 1H, CH_3_). ^13^C NMR (CDCl_3_, 100 MHz): δ (ppm) 184.47, 156.87, 140.18, 136.14, 136.07, 135.27, 129.65, 129.41, 128.95, 128.67, 127.22, 122.65, 21.36.

#### 3-(4-Bromophenyl)-1-((4-methoxyphenyl)sulfonyl)-1*H*-pyrazole-4-carbaldehyde (4e)

Yield: 62%, m.p. = 110–112°C from AcOEt/n-Hexane. ^1^H NMR (DMSO-d_6_, 400 MHz): δ (ppm) 9.94 (s, 1H, CHO), 9.33 (s, 1H, pyrazole-H5), 8.07 (d, 2H, H2, H6, *J* 2−3 = 8.0 Hz), 7.75 (d, 2H, H2′, H6′, *J*${}_{2^{\prime }-3^{\prime }}$ = 8.0 Hz), 7.67 (d, 2H, H3′, H5′, *J*2′-3′ = 8.0 Hz), 7.23 (d, 2H, H3, H5, *J*_2−3_ = 8.0 Hz), 3.87 (s, 1H, OCH_3_). ^13^C NMR (DMSO-d_6_, 100 MHz): δ (ppm) 184.84, 164.93, 153.43, 140.27, 131.46, 130.96, 130.70, 129.24, 125.86, 123.43, 122.32, 115.50, 56.09.

#### 3-(4-Chlorophenyl)-1-((4-methoxyphenyl)sulfonyl)-1*H*-pyrazole-4-carbaldehyde (4f)

Yield: 35%, m.p. = 114–115°C from AcOEt/n-Hexane. ^1^H NMR (DMSO-d_6_, 400 MHz): δ (ppm) 9.93 (s, 1H, CHO), 9.34 (s, 1H, pyrazole-H5), 8.07 (d, 2H, H2, H6, *J* 2−3 = 8.0 Hz), 7.82 (d, 2H, H2′, H6′, *J*${}_{2^{\prime }-3^{\prime }}$ = 8.0 Hz), 7.52 (d, 2H, H3′, H5′, *J*2′-3′ = 8.0 Hz), 7.23 (d, 2H, H3, H5, *J* 2−3 = 8.0 Hz), 3.87 (s, 1H, OCH_3_). ^13^C NMR (DMSO-d_6_, 100 MHz): δ (ppm) 185.15, 165.42, 153.85, 140.94, 135.17, 131.46, 130.98, 129.35, 129.01, 126.33, 122.81, 115.98, 56.58.

#### 1-((4-Methoxyphenyl)sulfonyl)-3-(p-tolyl)-1*H*-pyrazole-4-carbaldehyde (4g)

Yield: 68%, m.p. = 107–108°C from AcOEt/n-Hexane. ^1^H NMR (DMSO-d_6_, 400 MHz): δ (ppm) 9.94 (s, 1H, CHO), 9.27 (s, 1H, pyrazole-H5), 8.07 (d, 2H, H2, H6, *J*2−3 = 8.0 Hz), 7.68 (d, 2H, H2′, H6′, *J* ${}_{2^{\prime }-3^{\prime }}$ = 8.0 Hz), 7.27 (d, 2H, H3′, H5′, *J*2′-3′ = 8.0 Hz), 7.22 (d, 2H, H3, H5, *J* 2−3 = 8.0 Hz), 3.86 (s, 1H, OCH_3_), 2.35 (s, 1H, -CH_3_). ^13^C NMR (DMSO-d_6_, 100 MHz): δ (ppm) 185.24, 165.34, 155.25, 140.26, 140.04, 131.40, 129.52, 129.11, 127.65, 126.52, 122.79, 115.94, 56.55, 21.36.

### General Procedure for the Synthesis of (*E*)-*N*-((3-Phenyl-1-(Phenylsulfonyl)-1*H*-Pyrazol-4-yl)Methylene)Anilines (5a-p and 6a-l)

The suitable aniline (1.09 mmol) was added to a solution of the appropriate 1-phenylsulfonyl-1*H*-pyrazol-4-carbaldehyde **4a-g** (1.2 mmol) in dry ethanol (30 mL) and glacial acetic acid (0.1 mL). The mixture was refluxed for 6 h under magnetic stirring. After cooling, water was added and ethanol was removed under reduced pressure. The obtained suspension was extracted with diethyl ether and the organic layer was washed with brine, dried on Na_2_SO_4_ anhydrous, filtered and evaporated to dryness. The residual oil was used for the next reaction without further purification.

### General Procedure for the Synthesis of *N*-[(3-Phenil-1-(Phenylsulfonyl)-1*H*-Pyrazol-4-il)Methyl]Anilines (7a-p and 8a-l)

To a stirred solution of the crude 1-phenylsulphonyl-1*H*-pirazol-4-yl-methyleneaniline **5a-p** or **6a-l** (1 mmol) in dry THF (17 mL), NaBH_4_ (10 mmol) was added and the mixture was stirred at room temperature for 24 h. After this period, water was added and THF was removed under reduced pressure. The suspension was extracted with ethyl acetate and the organic phase was washed with brine, dried under Na_2_SO_4_ anhydrous, filtered and evaporated to dryness. The residue obtained was purified by crystallization from suitable solvent.

#### *N*-[(3-Phenyl-1-(phenysulfonyl)-1*H*-pyrazol-4-yl)methyl]aniline (7a)

Yield: 64%, m.p. = 139°C, from CHCl_3_/n-Hexane. ^1^H NMR (CDCl_3_, 400 MHz): δ (ppm) 8.1 (s, 1H, pyrazole-H5), 8.01 (d, 2H, H2, H6, *J*_2−3_ = 8.0 Hz), 7.66–7.61 (m, 3H, H4, H2′, H6′), 7.52 (t, 2H, H3, H5, *J*_2−3_ = *J*_3−4_ = 8.0 Hz), 7.39–7.37 (m, 3H, H3′, H4′, H5′), 7.17 (t, 2H, H3′′, H5′′, *J*${}_{2^{\prime \prime }-3^{\prime \prime }}$ = *J*${}_{3^{\prime \prime }-4^{\prime \prime }}$ = 7.6 Hz), 6.76 (t, 1H, H4″, *J*${}_{3^{\prime \prime }-4^{\prime \prime }}$ = 7.6 Hz), 6.58 (d, 2H, H2″, H6″, *J*${}_{2^{\prime \prime }-3^{\prime \prime }}$ = 7.6 Hz), 4.27 (s, 2H, CH_2_). ^13^C NMR (CDCl_3_, 100 MHz): δ (ppm) 155.77, 147.26, 137.17, 134.45, 131.66, 131.45, 129.36, 129.34, 129.11, 128.70, 128.12, 128.01, 121.04, 118.32, 113.20, 39.20. MS-ESI: *m/z* 390 (M + H^+^).

#### 4-Chloro-*N*-[(3-phenyl-1-(phenylsulphonyl)-1*H*-pyrazol-4-yl)methyl]aniline (7b)

Yield: 14%, m.p. = 136–137°C from CHCl_3_/n-Hexane. ^1^H NMR (CDCl_3_, 400 MHz): δ (ppm) 8.09 (s, 1H, pyrazole-H5), 8.01 (d, 2H, H2, H6, *J*_2−3_ = 8.0 Hz), 7.65–7.60 (m, 3H, H4, H2′, H6′), 7.52 (t, 2H, H3, H5, *J*_2−3_ = *J*_3−−4_ = 8.0 Hz), 7.38–7.37 (m, 3H, H3′, H4′, H5′), 7.08 (d, 2H, H3″, H5″, *J*${}_{2^{\prime \prime }-3^{\prime \prime }}$ = 8.4 Hz), 6.49 (d, 2H, H2″, H6″, *J*${}_{2^{\prime \prime }-3^{\prime \prime }}$ = 8.4 Hz), 4.25 (s, 1H, CH_2_). ^13^C NMR (CDCl_3_, 100 MHz): δ (ppm) 155.79, 145.29, 137.11, 134.52, 131.74, 131.33, 129.39, 129.18, 128.72, 128.12, 127.98, 123.33, 120.35, 114.62, 39.42. MS-ESI: *m/z* 424 (M + H^+^).

#### 4-Bromo-*N*-[(3-phenyl-1-(phenylsulfonyl)-1*H*-pyrazol-4-yl)methyl]aniline (7c)

Yield: 14%, m.p. = 122°C from CHCl_3_/n-Hexane. ^1^H NMR (CDCl_3_, 400 MHz): δ (ppm) 8.09 (s, 1H, pyrazole-H5), 8.01 (d, 2H, H2, H6, *J*_2−3_ = 7.6 Hz), 7.66–7.59 (m, 3H, H4, H2′, H6′), 7.53 (t, 2H, H3, H5, *J*_2−3_ = *J*_3−5_ = 7.6 Hz), 7.37–7.35 (m, 3H, H3′, H4′, H5′), 7.22 (d, 2H, H3″, H5″, *J*${}_{2^{\prime \prime }-3^{\prime \prime }}$ = 7.6 Hz), 6.45 (d, 2H, H2″, H6″, *J*${}_{2^{\prime \prime }-3^{\prime \prime }}$ = 7.6 Hz), 4.25 (s, 2H, CH2). ^13^C NMR (CDCl_3_, 100 MHz): δ (ppm) 155.79, 145.67, 137.10, 134.53, 132.06, 131.75, 131.31, 129.40, 129.20, 128.73, 128.13, 127.98, 120.25, 115.13, 110.46, 39.34. MS-ESI: *m/z* 470 (M + H^+^).

#### 4-Methyl-*N*-[(3-phenyl-1-(phenylsulfonyl)-1*H*-pyrazol-4-yl)methyl]aniline (7d)

Yield: 62% m.p. = 199°C, from CHCl_3_/n-Hexane. IR: 3396, 1376, 1185 cm-1. ^1^H NMR (CDCl_3_, 400 MHz): δ (ppm) 8.12 (s, 1H, pyrazole-H5), 8.02 (d, 2H, H2, H6, *J*_2−3_ = 8.0 Hz), 7.64–7.61 (m, 3H, H4, H2′, H6′), 7.52 (t, 2H, H3, H5, *J*_2−3_ = *J*_3−4_ = 8.0 Hz), 7.38–7.36 (m, 3H, H3′, H4′, H5′), 6.97 (d, 2H, H3″, H5″, *J*${}_{2^{\prime \prime }-3^{\prime \prime }}$ = 7.2 Hz), 6.52 (d, 2H, H2″, H6″, *J*${}_{2^{\prime \prime }-3^{\prime \prime }}$ = 7.2 Hz), 4.25 (s, 2H, CH_2_), 2.24 (s, 3H, CH_3_). ^13^C NMR (CDCl_3_, 100 MHz): δ (ppm) 155.86, 144.51, 137.25, 134.41, 131.77, 131.47, 129.84, 129.34, 129.08, 128.67, 128.14, 128.05, 120.92, 113.77, 39.74, 20.42. MS-ESI: *m/z* 404 (M + H^+^).

#### *N*-[(3-(4-Bromophenyl)-1-(phenylsulfonyl)-1*H*-pyrazol-4-yl)methyl]aniline (7e)

Yield: 36%; m.p. = 110–11°C from AcOEt/n-Hexane. ^1^H NMR (CDCl_3_, 400 MHz): δ (ppm) 8.10 (s, 1H, pyrazole-H5), 8.01 (d, 2H, H2, H6, *J*_2−3_ = 7.2 Hz), 7.64 (t, 1H, H4, *J*_3−4_ = 7.2 Hz), 7.57–7.48 (m, 6H, H3, H5, H2′, H6′, H3′, H5′), 7.18 (t, 2H, H3″, H5″, *J*${}_{2^{\prime \prime }-3^{\prime \prime }}$ = *J*${}_{3^{\prime \prime }-4^{\prime \prime }}$ = 7.6 Hz), 6.77 (t, 1H, H4″, *J*${}_{3^{\prime \prime }-4^{\prime \prime }}$ = 7.6 Hz), 6.59 (d, 2H, H2″, H6″, *J*${}_{2^{\prime \prime }-3^{\prime \prime }}$ = 7.6 Hz), 4.23 (s, 2H, CH_2_). ^13^C NMR (CDCl_3_, 100 MHz): δ (ppm) 154.67, 147.24, 137.08, 134.57, 131.90, 131.86, 130.41, 129.56, 129.42, 129.40, 128.19, 123.53, 120.83, 118.48, 113.19, 39.12. MS-ESI: *m/z* 470 (M + H^+^).

#### 4-Chloro-*N*-[(3-(4-bromophenyl)-1-(phenysulfonyl)-1*H*-pyrazol-4-yl)methyl]aniline (7f)

Yield: 52%; m.p. = 137–138°C from AcOEt/n-Hexane. ^1^H NMR (CDCl_3_, 400 MHz): δ (ppm) 8.07 (s, 1H, pyrazole-H5), 8.01 (d, 2H, H2, H6, *J*_2−3_ = 8.0 Hz), 7.65 (t, 1H, H4, *J*_3−4_ = 8.0 Hz), 7.55–7.49 (m, 6H, H3, H5, H2′, H6′, H3′, H5′), 7.11 (d, 2H, H3″, H5″, *J*${}_{2^{\prime \prime }-3^{\prime \prime }}$ = 8.4 Hz), 6.49 (d, 2H, H2″, H6″, *J*${}_{2^{\prime \prime }-3^{\prime \prime }}$ = 8.4 Hz), 4.20 (s, 2H, CH_2_). ^13^C NMR (CDCl_3_, 100 MHz): δ (ppm) 154.61, 145.70, 136.99, 134.64, 131.94, 131.80, 130.31, 129.50, 129.45, 129.23, 128.18, 123.61, 123.10, 120.40, 114.24, 39.16. MS-ESI: *m/z* 504 (M + H^+^).

#### 4-Bromo-*N*-[(3-(4-bromophenyl)-1-(phenylsulfonyl)-1*H*-pyrazol-4-yl)methyl]aniline

**(7g)**. Yield: 42%; m.p. = 150–151°C from AcOEt/n-Hexane. ^1^H NMR (CDCl_3_, 400 MHz): δ (ppm) 8.07 (s, 1H, pyrazole-H5), 8.01 (d, 2H, H2, H6, *J*_2−3_ = 8.0 Hz), 7.65 (t, 1H, H4, *J*_3−4_ = 8.0 Hz), 7.56–7.49 (m, 6H, H3, H5, H2′, H6′, H3′, H5′), 7.24 (d, 2H, H3″, H5″, *J*${}_{2^{\prime \prime }-3^{\prime \prime }}$ = 8.4 Hz), 6.44 (d, 2H, H2″, H6″, *J*${}_{2^{\prime \prime }-3^{\prime \prime }}$ = 8.4 Hz), 4.20 (s, 2H, CH_2_). ^13^C NMR (CDCl_3_, 100 MHz): δ (ppm) 154.59, 146.10, 136.98, 134.64, 132.10, 131.94, 131.79, 130.28, 129.48, 129.44, 128.17, 123.60, 120.32, 114.71, 110.14, 39.04. MS-ESI: *m/z* 548 (M + H^+^).

#### 4-Methyl-*N*-[(3-(4-bromophenyl)-1-(phenylsulfonyl)-1*H*-pyrazol-4-yl)methyl]aniline (7h)

Yield: 37%; m.p. = 112°C from AcOEt/n-Hexane. ^1^H NMR (CDCl_3_, 400 MHz): δ (ppm) 8.09 (s, 1H, pirazolo-H5), 8.01 (d, 2H, H2, H6, *J*_2−3_ = 8.0 Hz), 7.63 (t, 1H, H4, *J*_3−4_ = 8.0 Hz), 7.57–7.48 (m, 6H, H3, H5, H2′, H6′, H3′, H5′), 6.98 (d, 2H, H3″, H5″, *J*${}_{2^{\prime \prime }-3^{\prime \prime }}$ = 7.6 Hz), 6.51 (d, 2H, H2″, H6″, *J*${}_{2^{\prime \prime }-3^{\prime }}$ = 7.6 Hz), 4.19 (s, 2H, CH_2_), 2.24 (s, 3H, CH_3_). ^13^C NMR (CDCl_3_, 100 MHz): δ (ppm) 154.69, 144.99, 137.09, 134.54, 131.87, 130.44, 129.87, 129.57, 129.40, 128.17, 127.73, 123.48, 121.02, 113.36, 39.41, 20.41. MS-ESI: *m/z* 484 (M + H^+^).

#### *N*-[(3-(4-Chlorophenyl)-1-(phenylsulfonyl)-1*H*-pyrazol-4-yl)methyl]aniline (7i)

Yield: 21%, m.p. = 117–118°C from n-Hexane. IR: 3316, 1375, 1886 cm-1. ^1^H NMR (CDCl_3_, 400 MHz): δ (ppm) 8.12 (s, 1H, pyrazole-H5), 8.02 (d, 2H, H2, H6, *J*_2−3_ = 7.6 Hz), 7.64 (t, 1H, H4, *J*_3−4_ = 7.6 Hz), 7.60 (d, 2H, H2′, H6′, *J*${}_{2^{\prime }-3^{\prime }}$ = 7.6 Hz), 7.53 (t, 2H, H3, H5, *J*_2−3_ = J_3−4_ = 7.6 Hz), 7.33 (d, 2H, H3′, H5′, *J*${}_{2^{\prime }-3^{\prime }}$ = 7.6 Hz), 7.18 (t, 2H, H3″, H5″, J${}_{2^{\prime \prime }-3^{\prime \prime }}$ = *J*${}_{3^{\prime \prime }-4^{\prime \prime }}$ = 7.6 Hz), 6.78 (t, 1H, H4″, *J*${}_{3^{\prime \prime }-4^{\prime \prime }}$ = 7.6 Hz), 6.60 (d, 2H, H2″, H6″, *J*${}_{2^{\prime \prime }-3^{\prime \prime }}$ = 7.6 Hz), 4.24 (s, 2H, CH_2_). ^13^C NMR (CDCl_3_, 100 MHz): δ (ppm) 154.72, 146.96, 137.09, 135.22, 134.58, 131.98, 129.87, 129.42, 129.40, 129.31, 128.92, 128.17, 120.50, 118.84, 113.54, 39.30. MS-ESI: *m/z* 424 (M + H^+^).

#### 4-Cloro-*N*-[(3-(4-chlorophenyl)-1-(phenylsulfonyl)-1*H*-pyrazol-4-yl)methyl]aniline (7j)

Yield: 21%, m.p. = 142–143°C from n-Hexane. ^1^H NMR (CDCl_3_, 400 MHz): δ (ppm) 8.12 (s, 1H, pyrazole-H5), 8.02 (d, 2H, H2, H6, *J*_2−3_ = 8.0 Hz), 7.66 (t, 1H, H4, *J*_3−4_ = 8.0 Hz), 7.58–7.53 (m, 4H, H2′, H6′, H3, H5), 7.35 (d, 2H, H3′, H5′, *J*${}_{2^{\prime }-3^{\prime }}$ = 6.8 Hz), 7.10 (d, 2H, H3″, H5″, *J*${}_{2^{\prime \prime }-3^{\prime \prime }}$ = 7.2 Hz), 6.52 (d, 2H, H2″, H6″, *J*${}_{2^{\prime \prime }-3^{\prime \prime }}$ = 7.2 Hz), 4.20 (s, 2H, CH_2_). ^13^C NMR (CDCl_3_, 100 MHz): δ (ppm) 154.67, 144.97, 136.93, 135.32, 134.66, 131.98, 129.74, 129.46, 129.43, 129.25, 128.96, 128.18, 123.73, 119.92, 114.78, 39.44. MS-ESI: *m/z* 458 (M + H^+^).

#### 4-Bromo-*N*-[(3-(4-chlorophenyl)-1-(phenylsulfonyl)-1*H*-pyrazol-4-yl)methyl]aniline (7k)

Yield: 28%, m.p. = 142–143°C from n-Hexane. ^1^H NMR (CDCl_3_, 400 MHz): δ (ppm) 8.14 (s, 1H, pyrazole-H5), 8.02 (d, 2H, H2, H6, *J*_2−3_ = 8.0 Hz), 7.67 (t, 1H, H4, *J*_3−4_ = 8.0 Hz), 7.57–7.54 (m, 4H, H2′, H6′, H3, H5), 7.35 (d, 2H, H3″, H5″, *J*2″−3″ = 7.6 Hz), 7.24 (d, 2H, H3′, H5′, *J*${}_{2^{\prime }}$${}_{-3^{\prime }}=$ 8.4 Hz), 6.48 (d, 2H, H2″, H6″, *J*${}_{2^{\prime \prime }-3^{\prime \prime }}$ = 7.6 Hz), 4.24 (s, 2H, CH_2_). ^13^C NMR (CDCl_3_, 100 MHz): δ (ppm) 154.66, 145.40, 136.95, 135.33, 134.66, 132.12, 132.01, 129.74, 129.46, 129.25, 128.96, 128.17, 119.89, 115.28, 110.83, 39.35. MS-ESI: *m/z* 504 (M + H^+^).

#### 4-Methyl-*N*-[(3-(4-chlorophenyl)-1-(phenylsulfonyl)-1*H*-pyrazol-4-yl)methyl]anyline (7l)

Yield 29%, m.p. = 100–101°C from n-Hexane. ^1^H NMR (CDCl_3_, 400 MHz): δ (ppm) 8.13 (s, 1H, pyrazole-H5), 8.02 (d, 2H, H2, H6, *J*_2−3_ = 8.0 Hz), 7.66–7.60 (m, 3H, H4, H2, H6), 7.53 (t, 2H, H3, H5, *J*_2−3_ = *J*_3−4_ = 8.0 Hz), 7.33 (d, 2H, H3′, H5′, *J*${}_{2^{\prime }-3^{\prime }}$ = 8.0 Hz), 6.98 (d, 2H, H3″, H5″, *J*${}_{2^{\prime \prime }-3^{\prime \prime }}$ = 7.6 Hz), 6.53 (d, 2H, H2″, H6″, *J*${}_{2^{\prime \prime }-3^{\prime \prime }}$ = 7.6 Hz), 4.21 (s, 3H, CH_3_), 2.25 (s, 2H, CH_2_). ^13^C NMR (CDCl_3_, 100 MHz): δ (ppm) 154.72, 144.38, 137.04, 135.22, 134.55, 131.98, 129.88, 129.41, 129.33, 128.90, 128.27, 128.17, 120.61, 113.79, 39.65, 20.44. MS-ESI: *m/z* 438 (M + H^+^).

#### *N*-[(1-(Phenylsulfonyl)-3-(p-tolyl)-1*H*-pyrazol-4-yl)methyl]aniline (7m)

Yield: 20%, m.p. = 128–129°C from AcOEt/n-Hexane. ^1^H NMR (CDCl_3_, 400 MHz): δ (ppm) 8.09 (s, 1H, pyrazole-H5), 7.99 (d, 2H, H2, H6, *J*_2−3_ = 8.0 Hz), 7.60 (t, 1H, H4, *J*_3−4_ = 8.0 Hz), 7.54–7.47 (m, 4H, H3, H5, H2′, H6′), 7.17–7.14 (m, 4H, H3′, H5′, H3″, H5″), 6.76 (t, 1H, H4″, *J*${}_{3^{\prime \prime }-4^{\prime \prime }}$ = 8.0 Hz), 6.58 (d, 2H, H2″, H6″, *J*${}_{2^{\prime \prime }-3^{\prime \prime }}$ = 8.0 Hz), 4.25 (s, 2H, CH_2_), 2.34 (s, 3H, CH_3_). ^13^C NMR (CDCl_3_, 100 MHz): δ (ppm) 155.89, 146.92, 139.12, 137.25, 134.37, 131.72, 129.38, 129.33, 128.55, 128.08, 127.90, 120.76, 118.57, 114.96, 113.49, 39.39, 21.29. MS-ESI: *m/z* 404 (M + H^+^).

#### 4-Chloro-*N*-[(1-(phenylsulfonyl)-3-(p-tolyl)-1*H*-pyrazol-4-yl)methyl]aniline (7n)

Yield: 22%, m.p. = 115°C from AcOEt/n-Hexane. ^1^H NMR (CDCl_3_, 400 MHz): δ (ppm) 8.05 (s, 1H, pyrazole-H5), 7.98 (d, 2H, H2, H6, *J*_2−3_ = 7.2 Hz), 7.61 (t, 1H, H4, *J*_3−4_ = 7.2 Hz), 7.54–7.48 (m, 4H, H3, H5, H2′, H6′), 7.17 (d, 2H, H3′, H5′, *J*${}_{2^{\prime }-3^{\prime }}$ = 7.2 Hz), 7.07 (d, 2H, H3″, H5″, *J*${}_{2^{\prime \prime }-3^{\prime \prime }}$ = 8.0 Hz), 6.47 (d, 2H, H2″, H6″, *J*${}_{2^{\prime \prime }-3^{\prime \prime }}$ = 8.0 Hz), 4.25 (s, 2H, CH_2_) 2.34 (s, 3H, CH_3_) ^13^C NMR (CDCl_3_, 100 MHz): δ (ppm) 155.85, 145.52, 139.22, 137.15, 134.44, 131.66, 129.41, 129.35, 129.13, 128.45, 128.06, 127.84, 123.05, 120.43, 114.47, 39.36, 21.29. MS-ESI: *m/z* 438 (M + H^+^).

#### 4-Bromo-*N*-[(1-(phenylsulfonyl)-3-(p-tolyl)-1*H*-pyrazol-4-yl)methyl]aniline (7o)

Yield: 24%, m.p. = 110°C from AcOEt/n-Hexane. ^1^H NMR (CDCl_3_, 400 MHz): δ ppm 8.03 (s, 1H, pyrazole-H5), 7.98 (d, 2H, H2, H6, *J*_2−3_ = 8.0 Hz), 7.61 (t, 1H, H4, *J*_3−4_ = 8.0 Hz), 7.51–7.48 (m, 4H, H3, H5, H2′, H6′), 7.21–7.15 (m, 4H, H3′, H5′, H3″, H5″), 6.42 (d, 2H, H2″, H6″, *J*${}_{2^{\prime \prime }-3^{\prime \prime }}$ = 7.6 Hz), 4.20 (s, 2H, CH_2_), 2.33 (s, 3H, CH_3_). ^13^C NMR (CDCl_3_, 100 MHz): δ (ppm) 155.84, 146.00, 139.22, 137.14, 134.44, 131.99, 131.66, 129.41, 129.35, 128.45, 128.05, 127.84, 120.41, 114.91, 110.05, 39.23, 21.29. MS-ESI: *m/z* 484 (M + H^+^).

#### 4-Methyl-*N*-[(1-(phenysulfonyl)-3-(p-tolyl)-1*H*-pyrazol-4-yl)methyl]aniline (7p)

Yield: 41%, m.p. = 123–124°C from AcOEt/n-Hexane. ^1^H (CDCl_3_, 400 MHz): δ (ppm) 8.08 (s, 1H, pyrazole-H5), 8.00 (d, 2H, H2, H6, *J*_2−3_ = 7.2 Hz), 7.61 (t, 1H, H4, *J*_3−4_ = 7.2 Hz), 7.55–7.47 (m, 4H, H3, H5, H2′, H6′), 7.17 (d, 2H, H3′, H5′, *J*${}_{2^{\prime }-3^{\prime }}$ = 7.6 Hz), 6.97 (d, 2H, H3″, H5″, *J*${}_{2^{\prime \prime }-3^{\prime \prime }}$ = 8.0 Hz), 6.51 (d, 2H, H2″, H6″, *J*${}_{2^{\prime \prime }-3^{\prime \prime }}$ = 8.0 Hz), 4.20 (s, 2H, CH_2_), 2.34 (s, 3H, CH_3_-Ar), 2.24 (s, 3H, CH_3_-Ar″). ^13^C (CDCl_3_, 100 MHz): δ (ppm) 155.90, 144.75, 139.09, 137.29, 134.34, 131.68, 129.81, 129.36, 129.31, 128.61, 128.08, 127.91, 127.78, 121.00, 113.61, 39.69, 21.29, 20.42. MS-ESI: *m/z* 418 (M + H^+^).

#### *N*-[(3-Bromophenyl)-1-((4-methoxphenyl)sulfonyl)-1*H*-pyrazol-4-yl)methyl]aniline (8a)

Yield: 41%, m.p. = 131–134°C from CHCl_3_/n-Hexane. ^1^H NMR (CDCl_3_, 400 MHz): δ (ppm) 7.96 (s, 1H, pyrazole-H5), 7.58 (d, 2H, H2′, H6′, *J*${}_{2^{\prime }-3^{\prime }}$ = 8.8 Hz), 7,57 (d, 2H, H2, H6, *J*_2−3_ = 8.4 Hz), 7.52(d, 2H, H3′, H5′, *J*${}_{2^{\prime }-3^{\prime }}$ = 8.8 Hz), 7.19 (t, 2H, H3″, H5″, *J*${}_{2^{\prime \prime }-3^{\prime \prime }}$ = *J*${}_{3^{\prime \prime }-4^{\prime \prime }}$ = 8.0 Hz), 6.98 (d, 2H, H3, H5, *J*_2−3_ = 8.4 Hz), 6.78 (t, 1H, H4″, *J*${}_{3^{\prime \prime }-4^{\prime \prime }}$ = 8.0 Hz), 6.60 (d, 2H, H2″, H6″, *J*${}_{2^{\prime \prime }-3^{\prime \prime }}$ = 8.0 Hz), 4.24 (s, 2H, CH_2_), 3.86 (s, 3H, OCH_3_). ^13^C NMR (CDCl_3_, 100 MHz): δ (ppm) 164.04, 146.71, 144.43, 131.37, 131.10, 130.15, 130.02, 129.06, 128.89, 127.65, 122.90, 119.80, 117.98, 114.15, 112.68, 55.28, 38.59. MS-ESI: *m/z* 498 (M + H^+^).

#### 4-Chloro-*N*-[(3-(4-bromophenyl)-1-((4-methoxphenyl)sulfonyl)-1*H*-pyrazol-4-yl)methyl]aniline (8b)

Yield: 50%, m.p. = 153–154°C from CHCl_3_/n-Hexane. ^1^H NMR (CDCl_3_, 400 MHz): δ (ppm) 8.07 (s, 1H, pyrazole-H5), 7.95 (d, 2H, H2, H6, *J*_2−3_ = 8.8 Hz), 7.52 (m, 4H, H2′, H6′, H3′, H5′), 7.12 (d, 2H, H3″, H5″, *J*${}_{2^{\prime \prime }-3^{\prime \prime }}$ = 8.8 Hz), 6.98 (d, 2H, H3, H5, *J*_2−3_ = 8.8 Hz), 6.50 (d, 2H, H2″, H6″, *J*${}_{2^{\prime \prime }-3^{\prime \prime }}$ = 8.8 Hz), 4.21 (s, 2H, CH_2_), 3.86 (s, 3H, OCH_3_). ^13^C NMR (CDCl_3_, 100 MHz): δ (ppm) 164.05, 153.78, 144.81, 132.55, 131.39, 131.14, 130.15, 129.88, 129.00, 128.73, 127.53, 122.97, 119.17, 114.17, 114.02, 55.31, 38.80. MS-ESI: *m/z* 534 (M + H^+^).

#### 4-Bromo-*N*-[(3-(4-bromophenyl)-1-((4-methoxphenyl)sulfonyl)-1*H*-pyrazol-4-yl)methyl]aniline (8c)

Yield: 63%, m.p. = 162–164°C from CHCl_3_/n-Hexane. ^1^H NMR (CDCl_3_, 400 MHz): δ (ppm) 8.06 (s, 1H, pyrazole-H5), 7.94 (d, 2H, H2′, H6′, *J*${}_{2^{\prime }-3^{\prime }}$ = 8.0 Hz), 7.52 (m, 4H, H2, H6, H3′, H5′), 7.25 (d, 2H, H3″, H5″, *J*${}_{2^{\prime \prime }-3^{\prime \prime }}$ = 8.8 Hz), 6.98 (d, 2H, H3, H5, *J*_2−3_ = 8.8 Hz), 6.46 (d, 2H, H2″, H6″, *J*${}_{2^{\prime \prime }-3^{\prime \prime }}$ = 8.8 Hz), 4.20 (s, 2H, CH_2_), 3.86 (3H, -OCH_3_). ^13^C NMR (CDCl_3_, 100 MHz): δ (ppm) 164.04, 153.41, 145.57, 132.55, 131.58, 131.40, 131.06, 130.14, 129.90, 128.98, 127.52, 122.97, 119.33, 114.22, 114.17, 55.32, 38.53. MS-ESI: *m/z* 578 (M + H^+^).

#### 4-Methyl-*N*-[(3-(4-bromophenyl)-1-((4-methoxphenyl)sulfonyl)-1*H*-pyrazol-4-yl)methyl]aniline (8d)

Yield: 50%, m.p. = 164–166°C from CHCl_3_/n-Hexane. ^1^H NMR (CDCl_3_, 400 MHz): (ppm) 8.06 (s, 1H, pyrazole-H5), 7.99 (d, 2H, H2′, H6′, *J*${}_{2^{\prime }-3^{\prime }}$ = 8.8 Hz), 7.61 (d, 2H, H2, H6, *J*_2−6_ = 8.8 Hz), 7.53 (d, 2H, H3′, H5′, *J*${}_{2^{\prime }-3^{\prime }}$ = 8.8 Hz), 7.03–6.99 (m, 4H, H3, H5, H3″, H5″), 6.55 (d, 2H, H2″, H6″, *J*${}_{2^{\prime \prime }-3^{\prime \prime }}$ = 8.0 Hz), 4.23 (s, 2H, CH_2_), 3.89 (s, 3H, OCH_3_), 2.28 (s, 3.H, CH_3_). ^13^C NMR (CDCl_3_, 100 MHz): δ (ppm) 163.98, 153.85, 144.44, 131.34, 131.09, 130.14, 129.37, 129.08, 127.68, 127.27, 122.86, 120.22, 119.98, 114.14, 112.87, 55.28, 38.91, 19.99. MS-ESI: *m/z* 514 (M + H^+^).

#### *N*-[(3-(4-Chlorophenyl)-1-(4-methoxphenyl)sulfonyl)-1*H*-pyrazol-4-yl)methy])aniline (8e)

Yield: 42%, m.p. = 143–145°C from CHCl_3_/n-Hexane. ^1^H NMR (CDCl_3_, 400 MHz): δ (ppm) 8.09 (s, 1H, pyrazole-H5), 7.95 (d, 2H, H2′, H6′, *J*${}_{2^{\prime }-3^{\prime }}$ = 8.0 Hz), 7.64 (d, 2H, H2, H6, *J*_2−3_ = 8.0 Hz), 7.35 (d, 2H, H3′, H5′, *J*${}_{2^{\prime }-3^{\prime }}$ = 8.0 Hz), 7.19 (t, 2H, H3″, H5″ *J*${}_{2^{\prime \prime }-3^{\prime \prime }}$ = *J*${}_{3^{\prime \prime }-4^{\prime \prime }}$ = 8.0 Hz), 6.98 (d, 2H, H3, H5, *J*_2−3_ = 8.0 Hz), 6.77 (t, 1H, H4″, *J*${}_{3^{\prime \prime }-4^{\prime \prime }}$ = 8.0 Hz), 6.60 (d, 2H, H2″, H6″, *J*${}_{2^{\prime \prime }-3^{\prime \prime }}$ = 8.0 Hz), 4.24 (s, 2H, CH_2_), 3.85 (s, 3H, OCH_3_). ^13^C NMR (CDCl_3_, 100 MHz): δ (ppm) 163.99, 153.78, 146.73, 134.60, 131.09, 130.14, 129.56, 128.88, 128.80, 128.41, 127.67, 119.83, 117.94, 114.16, 112.68, 55.29, 38.60. MS-ESI: *m/z* 454 (M + H^+^).

#### 4-Chloro-*N*-[(3-(4-chlorophenyl)-1-((4-methoxyphenyl)sulfonyl)-1*H*-pyrazol-4-yl)methyl]aniline (8f)

Yield: 36%, m.p. = 158–159°C from AcOEt/n-Hexane. ^1^H NMR (CDCl_3_, 400 MHz): δ (ppm) 8.08 (s, 1H, pyrazole-H5), 7.94 (d, 2H, H2′, H6′, *J*${}_{2^{\prime }-3^{\prime }}$ = 8.0 Hz), 7.59 (d, 2H, H2, H6 *J*_2−3_ = 7.2 Hz), 7.35 (d, 2H, H3′, H5′, *J*${}_{2^{\prime }-3^{\prime }}$ = 8.0 Hz), 7.12 (d, 2H, H3″, H5″, *J*${}_{2^{\prime \prime }-3^{\prime \prime }}$ = 8.0 Hz), 6.98 (d, 2H, H3, H5 *J*_2−3_ = 7.2 Hz), 6.51 (d, 2H, H2″, H6″, *J*${}_{2^{\prime \prime }-3^{\prime \prime }}$ = 8.0 Hz), 4.21 (s, 2H, CH_2_), 3.87 (s, 3H, OCH_3_). ^13^C NMR (CDCl_3_, 100 MHz): δ (ppm) 164.53, 154.26, 145.48, 135.19, 133.05, 131.61, 130.64, 129.92, 129,24, 128.95, 128.01, 123.21, 119.77, 114.40, 114.39, 55.82, 39.22. MS-ESI: *m/z* 488 (M + H^+^).

#### 4-Bromo-*N*-[(3-(4-chlorophenyl)-1-((4-methoxyphenyl)sulfonyl)-1*H*-pyrazol-4-yl)methyl]aniline (8g)

Yield: 35%, m.p. = 169–171°C from AcOEt/n-Hexane. ^1^H NMR (CDCl_3_, 400 MHz): δ (ppm) 8.07 (s, 1H, pyrazole-H5), 7.95 (d, 2H, H2′, H6′, *J*${}_{2^{\prime }-3^{\prime }}$ = 8.0 Hz), 7.59 (d, 2H, H2, H6 *J*_2−3_ = 7.2 Hz), 7.35 (d, 2H, H3′, H5′, *J*${}_{2^{\prime }-3^{\prime }}$ = 8.0 Hz), 7.26 (m, 2H, H3″, H5″), 6.99 (d, 2H, H3, H5, *J*_2−3_ = 7.2 Hz), 6.46 (d, 2H, H2″, H6″, *J*${}_{2^{\prime \prime }-3^{\prime \prime }}=$ 8.0 Hz), 4.22 (s, 2H, CH_2_), 3.87 (s,3H, OCH_3_). ^13^C NMR (CDCl_3_, 100 MHz): δ (ppm) 164.55, 154.22, 146.01, 135.19, 132.09, 131.60, 130.63, 129.94, 129.23, 128.94, 128.04, 119.80, 114.82, 114.68, 110.18, 55.82, 39.09. MS-ESI: *m/z* 534 (M + H^+^).

#### 4-Methyl-*N*-[(3-(4-chlorophenyl)-1-((4-methoxyphenyl)sulfonyl)-1*H*-pyrazol-4-yl)methyl]aniline (8h)

Yield: 35%, m.p. = 169–171°C from AcOEt/n-Hexane. ^1^H NMR (CDCl_3_, 400 MHz): δ (ppm) 8.12 (s, 1H, pyrazole-H5), 7.99 (d, 2H, H2′, H6′, *J*${}_{2^{\prime }-3^{\prime }}$ = 8.0 Hz), 7.64 (d, 2H, H2, H6, *J*_2−3_ = 7.2 Hz), 7.37 (d, 2H, H3′, H5′, *J*${}_{2^{\prime }-3^{\prime }}$ = 8.0 Hz), 7.01 (m, 4H, H3, H5, H3″, H5″), 6.46 (d, 2H, H2″, H6″, *J*${}_{2^{\prime \prime }-3^{\prime \prime }}=$ 8.0 Hz), 4.22 (s, 2H, CH_2_), 3.87 (s, 3H, OCH_3_), 2.28 (s, 3H, -CH_3_). ^13^C NMR (CDCl_3_, 100 MHz): δ (ppm) 164.48, 154.33, 144.82, 135.07, 131.61, 130.63, 130.10, 129.87, 129.32, 128.88, 128.23, 127.89, 120.42, 114.64, 113.50, 55.78, 39.50, 20.42. MS-ESI: *m/z* 468 (M + H^+^).

#### *N*-[(1-((4-Methoxyphenyl)sulfonyl)-3-(p-tolyl)-1*H*-pyrazol-4-yl)methyl]aniline (8i)

Yield: 40%, m.p. = 154–155°C from AcOEt/n-Hexane. 1H NMR (CDCl_3_, 400 MHz): δ (ppm) 7.97 (s, 1H, pyrazole-H5), 7.95 (d, 2H, H2, H6, *J*_2−3_ = 8.0 Hz), 7.54 (d, 2H, H2′, H6′, *J*${}_{2^{\prime }-3^{\prime }}$ = 8.0 Hz), 7.19–7.15 (m, 4H, H3′, H5′, H3″, H5″), 6.96 (d, 2H, H3, H5, *J*_2−3_ = 8.0), 6.77 (t, 1H, H4″ *J*${}_{3^{\prime \prime }-4^{\prime \prime }}=$ 7.6 Hz), 6.60 (d, 2H, H2″, H6″, *J*${}_{2^{\prime \prime }-3^{\prime \prime }}$ = 7.6 Hz), 4.27 (s, 2H, CH_2_), 3.85 (s, 3H, OCH_3_), 2.35 (s, 3H, CH_3_). ^13^C NMR (CDCl_3_, 100 MHz): δ (ppm) 163.82, 154.99, 146.88, 138.52, 130.83, 130.05, 128.88, 128.83, 128.22, 127.93, 127.39, 120.04, 117.70, 114.06, 112.63, 55.26, 38.70, 20.82. MS-ESI: *m/z* 434 (M + H^+^).

#### 4-Chloro-*N*-[(1-(4-methoxyphenyl)sulfonyl)-3-(p-tolyl)-1*H*-pyrazol-4-yl)methyl]aniline (8j)

Yield: 56%, m.p. = 157–158°C from AcOEt/n-Hexane. ^1^H NMR (CDCl_3_, 400 MHz): δ (ppm) 8.03 (s, 1H, pyrazole-H5), 7.93 (d, 2H, H2, H6, *J*_2−3_ = 7.2 Hz), 7.52 (d, 2H, H2′, H6′, *J*${}_{2^{\prime }-3^{\prime }}$ = 8.0 Hz), 7.18 (d, 2H, H3, H5, *J*_2−3_ = 8.0 Hz), 7.09 (d, 2H, H3″, H5″, *J*${}_{2^{\prime \prime }-3^{\prime \prime }}$ = 8.0 Hz), 6.95 (d, 2H, H3, H5, *J*_2−3_ = 7.2 Hz), 6.48 (d, 2H, H2″, H6″, *J*${}_{2^{\prime \prime }-3^{\prime \prime }}$ = 8.0 Hz), 4.22 (s, 2H, CH_2_), 3.85 (s, 3H, OCH_3_), 2.36 (s, 3H, CH_3_). ^13^C NMR (CDCl_3_, 100 MHz): δ (ppm) 164.38, 155.44, 145.83, 139.10, 131.32, 130.53, 129.40, 129.13, 128.63, 128.33, 127.84, 122.78, 120.11, 114.58, 114.24, 55.77, 39.26, 21.31. MS-ESI: *m/z* 468 (M + H^+^).

#### 4-Bromo-*N*-[(1-(4-methoxyphenyl)sulfonyl)-3-(p-tolyl)-1*H*-pyrazol-4-yl)methyl]aniline (8k)

Yield: 33%, m.p. = 151–153°C from AcOEt/n-Hexane. ^1^H NMR (CDCl_3_, 400 MHz): δ (ppm) 8.07 (s, 1H, pyrazole-H5), 7.96 (d, 2H, H2, H6, *J*_2−3_ = 8.8 Hz), 7.54 (d, 2H, H2′, H6′, *J*${}_{2^{\prime }-3^{\prime }}$ = 8.0 Hz), 7.28–7.20 (m, 4H, H3′, H5′, H3″, H5″), 7.01 (d, 2H, H3, H5, *J*_2−3_ = 8.8 Hz), 6.47 (d, 2H, H2″, H6″, *J*${}_{2^{\prime \prime }-3^{\prime \prime }}$ = 8.8 Hz), 4.26 (s, 2H, CH_2_), 3.88 (s, 3H, OCH_3_), 2.38 (s, 3H, CH_3_). ^13^C NMR (CDCl_3_, 100 MHz): δ (ppm) 164.36, 155.45, 146.26, 139.14, 132.01, 131.34, 130.54, 129.42, 128.57, 128.25, 127.83, 120.06, 114.68, 114.59, 109.77, 55.80, 39.12, 21.35. MS-ESI: *m/z* 514 (M + H^+^).

#### 4-Methyl-*N*-[(1-((4-methoxyphenyl)sulfonyl)-3-(p-tolyl)-1*H*-pyrazol-4-yl)methyl]aniline (8l)

Yield: 25%, m.p. = 145–146°C from CHCl_3_/n-Hexane. ^1^H NMR (CDCl_3_, 400 MHz): δ (ppm) 8.07 (s, 1H, pyrazole-H5), 7.95 (d, 2H, H2, H6, *J*_2−3_ = 8.8 Hz), 7.56 (d, 2H, H2′, H6′, *J*${}_{2^{\prime }-3^{\prime }}$ = 8.0 Hz), 7.18 (d, 2H, H3′, H5′, *J*${}_{2^{\prime }-3^{\prime }}$ 8.0 Hz), 6.99–6.95 (m, 4H, H3, H5, H3″, H5″), 6.53(d, 2H, H2″, H6″, *J*${}_{2^{\prime \prime }-3^{\prime \prime }}$ = 8.8 Hz), 4.24 (s, 2H, CH_2_), 3.85 (s, 3H, OCH_3_), 2.35 (s, 3H, CH_3_), 2.25 (s, 3H, CH_3_). ^13^C NMR (CDCl_3_, 100 MHz): δ (ppm) 163.81, 155.02, 144.41, 138.46, 130.86, 130.04, 129.31, 128.84, 128.26, 128.01, 127.41, 127.38, 120.10, 114.04, 113.00, 55.24, 39.14, 20.80, 19.92. MS-ESI: *m/z* 448 (M + H^+^).

### Biology

#### Test Compounds

New compounds and reference inhibitors were solubilized in DMSO at a concentration of 100 mM, and successively subjected to serial dilutions in the culture medium. The first dilution, with ratio 1:50, diluted the compounds from 100 mM to a concentration of 2 mM, reducing the percentage of DMSO from 100 to 2%. The second dilution, with ratio 1:20, leads the compounds from 2 mM to a concentration of 100 μM and reduces the concentration of DMSO from 2 to 0.1%, a non-toxic concentration for cells. Next dilutions, performed with ratio 1: 5, result in a further decrease in the percentage of DMSO in contact with the cells.

2′-C-methylguanosin (NM108), efavirenz (EFV), pleconaril, ribavirin, 6-azauridine were used as reference inhibitors for ssRNA^+^ viruses. 6-Azauridine, 2′-C-methylcitidine (NM107), acyclovir (ACG), and mycophenolic acid (M 5255) were used as reference compounds of ssRNA^−^, dsRNA and DNA viruses, respectively.

#### Cells and Viruses

Cell lines used to support the multiplication of RNA and DNA viruses were purchased from American Type Culture Collection (ATCC). The Hoechst staining method were employed to check periodically the absence of mycoplasma. Viruses were acquired from American Type Culture Collection (ATCC), with the exception of Yellow Fever Virus (YFV), Dengue virus type 2 (DENV-2), West Nile virus (WNV) and Human Immunodeficiency Virus type-1 (HIV-1).

Baby Hamster Kidney (BHK-21) [ATCC CCL10 (C-13) *Mesocricetus auratus*] cells were used for the replication of YFV [strain 17-D vaccine (Stamaril Pasteur J07B01)], DENV-2 [clinical isolate], WNV [clinical isolate], and reovirus type-1 (Reo-1) [simian virus 12, strain 3651 (ATCC VR- 214)]; Madin Darby Bovine Kidney (MDBK) [ATCC CCL22 (NBL-1) *Bos Taurus*] cells for bovine viral diarrhea virus (BVDV) [strain NADL (ATCC VR-534)]; Monkey kidney (Vero 76) [ATCCCRL 1587 *Cercopithecus Aethiops*] cells for human respiratory syncytial virus (RSV) [strain A2 (ATCC VR-1540)], human enterovirus B [coxsackie type B5 (CV-B5) [strain Faulkner (ATCC VR-185)], human enterovirus C [poliovirus type-1 (Sb-1), Sabin strain Chat (ATCC VR-1562)], vesicular stomatitis virus (VSV) [lab strain Indiana (ATCC VR 158)], vaccinia virus (VV) [strain Elstree (Lister Vaccine) (ATCC VR-1549)], and human herpesvirus 1 (HSV-1) [strain KOS (ATCC VR- 1493)]; CD4^+^ human T-cells containing an integrated HTLV-1 genome (MT-4) for the III_B_ laboratory strain of HIV-1, obtained from the supernatant of the persistently infected H9/III_B_ cells (NIH 1983).

#### Cytotoxicity Assays

Cytotoxicity tests were performed in parallel with antiviral tests following the procedures previously described by us (Fioravanti et al., 2017). In brief, exponentially growing MT-4 cells were seeded at an initial density of 1 × 10^5^ cells/mL in 96-well plates in RPMI-1640 medium, supplemented with 10% fetal bovine serum (FBS), 100 units/mL penicillin G and 100 mg/mL streptomycin. Cell cultures were then incubated at 37°C in a humidified, 5% CO_2_ atmosphere, in the absence or presence of serial dilutions of test compounds. Cell viability was determined after 96 h at 37°C by the 3-(4,5-dimethylthiazol-2-yl)-2,5-diphenyl-tetrazolium bromide (MTT) method. MDBK and BHK cells were seeded in 96-well plates at an initial density of 6 × 10^5^ and 1 × 10^6^ cells/mL, respectively, in Minimum Essential Medium with Earle′s salts (MEM-E), L glutamine, 1 mM sodium pyruvate and 25 mg/L kanamycin, supplemented with 10% horse serum (MDBK) or 10% fetal bovine serum (FBS) (BHK). Cell cultures were then incubated at 37°C in a humidified, 5% CO_2_ atmosphere in the absence or presence of serial dilutions of test compounds. Cell viability was determined after 72 h at 37°C by the MTT method. Vero76 cells were seeded in 96-well plates at an initial density of 5 × 10^5^ cells/mL, in Dulbecco′s Modified Eagle Medium (D-MEM) with L-glutamine and 25 mg/L kanamycin, supplemented with 10% FBS. Cell cultures were then incubated at 37°C in a humidified, 5% CO_2_ atmosphere in the absence or presence of serial dilutions of test compounds. Cell viability was determined after 48–96 h at 37°C by the by the MTT method (Pauwels et al., 1988).

#### Antiviral Assays

Antiviral activity of compounds and reference inhibitors was evaluated by using the experimental protocol reported previously (Fioravanti et al., 2017). Protection of BHK-21 cells from YFV, DENV-2, WNV and Reo-1 -induced cytopathogenicity were determined by the MTT method (Pauwels et al., 1988). Similarly, protection of MDBK and MT-4 cells from induced cytopathogenicity by BVDV and HIV-1, respectively, were assessed by the MTT method (Pauwels et al., 1988). Antiviral activity against RSV, CVB-5, Sb-1, VSV, VV, and HSV-1 was evaluated by a plaque reduction assay in infected Vero 76 cell monolayers.

#### Time of Addition Assay

Time-of-addition experiments were performed on BHK-21 cells infected with YFV. The effects of compound **7e** (36 μM = 10 × EC_50_) and reference inhibitor 6-azauridine (90 μM = 2 × EC_50_) were evaluated by using the experimental protocol reported previously (Fioravanti et al., 2017).

#### Linear Regression Analysis

The extent of cell growth/viability and viral multiplication, at each drug concentration tested, were expressed as percentage of untreated controls. Concentrations resulting in 50% inhibition (CC_50_ or EC_50_) were determined by linear regression analysis.

## Author Contributions

ND, RF, AC, and RL designed and supervised the study. RF, LP, and EA performed chemical synthesis, purification, and characterization of compounds. ID and GC performed the biological assays. All the authors contributed to the analysis, interpretation, and discussion of the data. ND drafted the manuscript. All the authors discussed and revised the manuscript.

### Conflict of Interest Statement

The authors declare that the research was conducted in the absence of any commercial or financial relationships that could be construed as a potential conflict of interest.
