# Supplementary material for: Design, Synthesis, Antiviral Evaluation, and SAR Studies of New 1-(Phenylsulfonyl)-1H-Pyrazol−4-yl-Methylaniline Derivatives
Source: Front Chem. 2019 Apr 9;7:214. doi: 10.3389/fchem.2019.00214 (PMC6465675; doi:10.3389/fchem.2019.00214)
Supplement: Supplementary file 1 [file Data_Sheet_1.PDF]

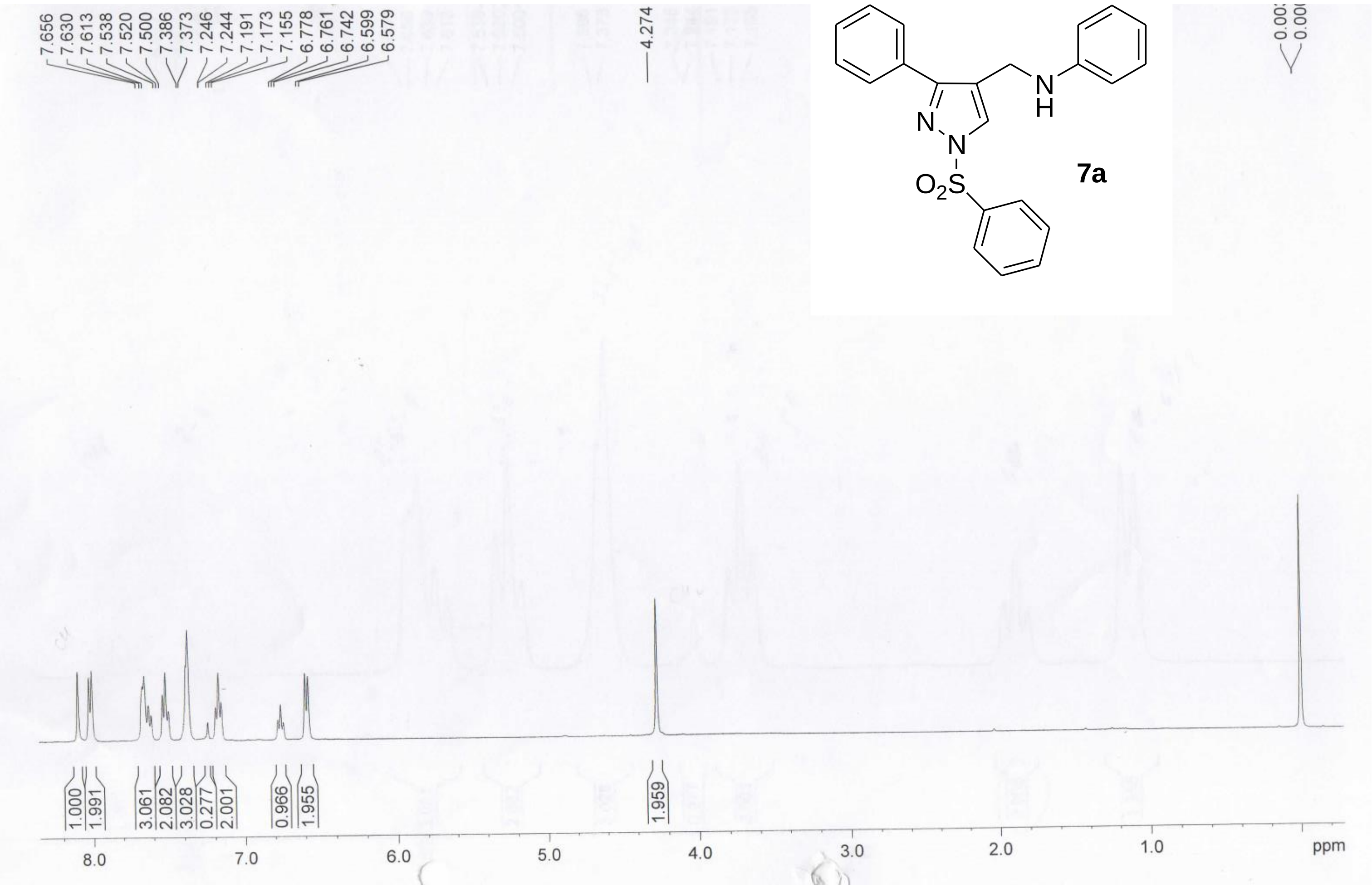

Fig S 1. ^{1}H NMR spectrum of compound **7a**

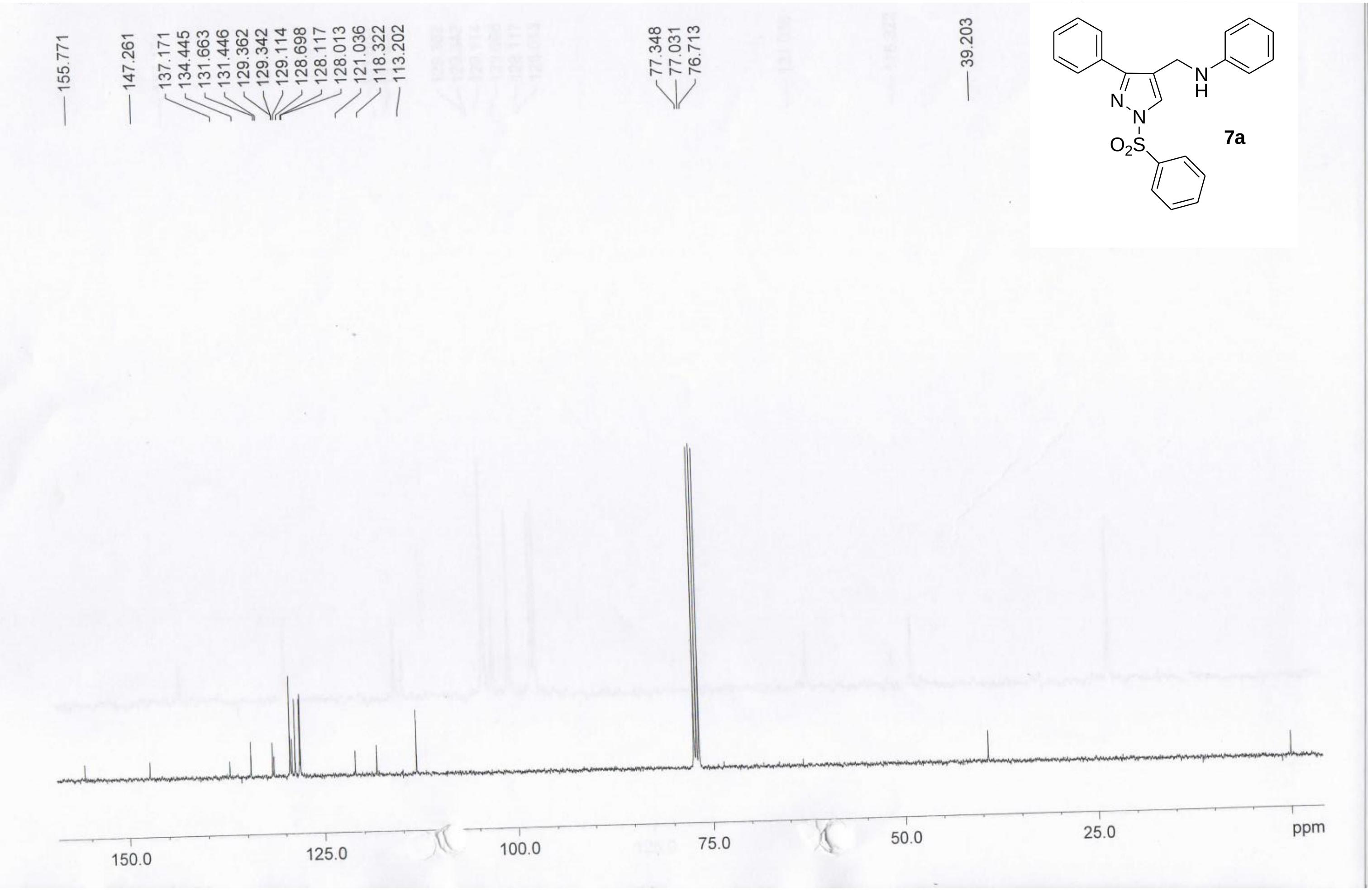

Fig S 1. ^{13}C NMR spectrum of compound **7a**

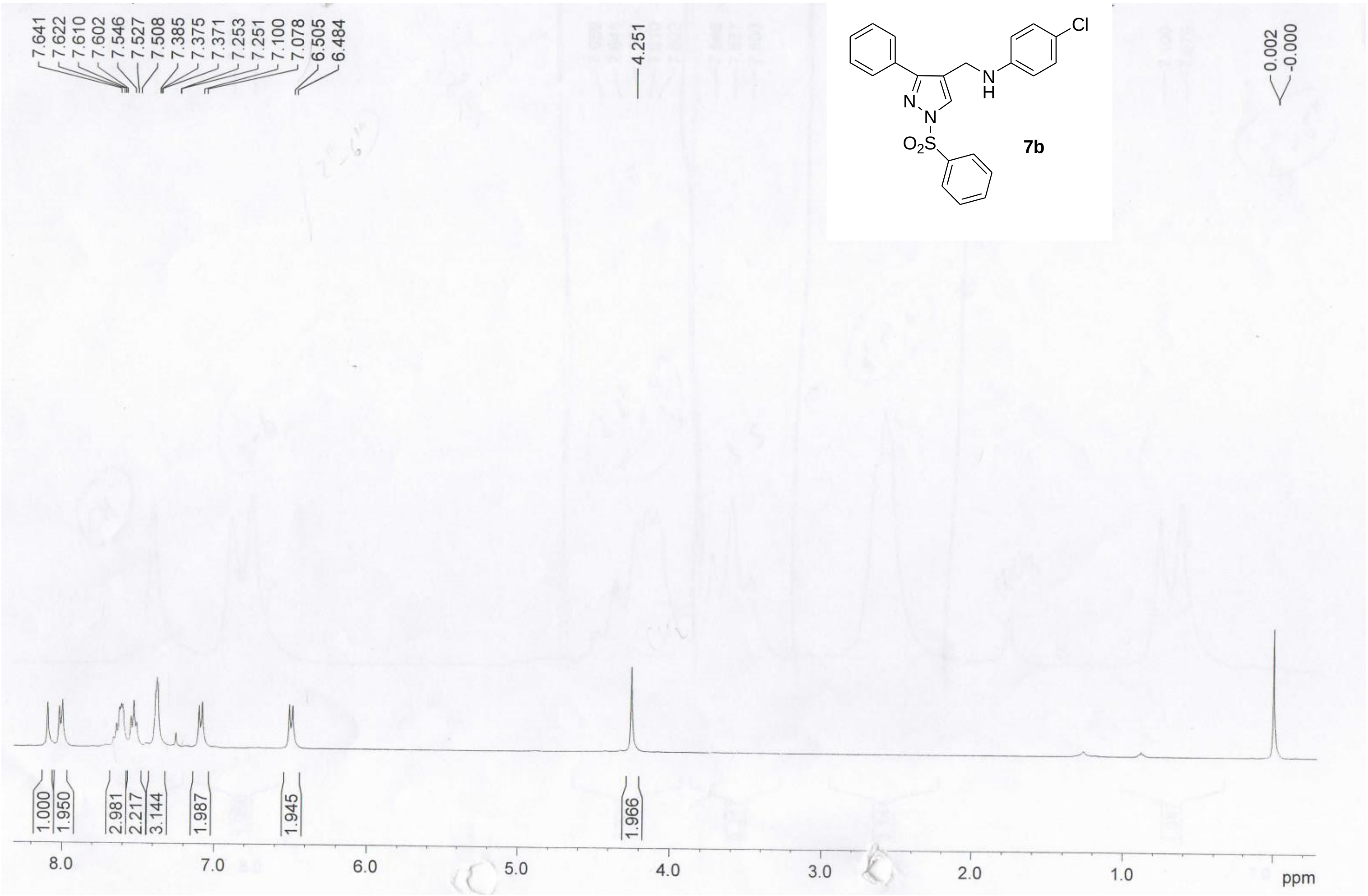

Fig. S 2. ^{1}H NMR spectrum of compound **7b**

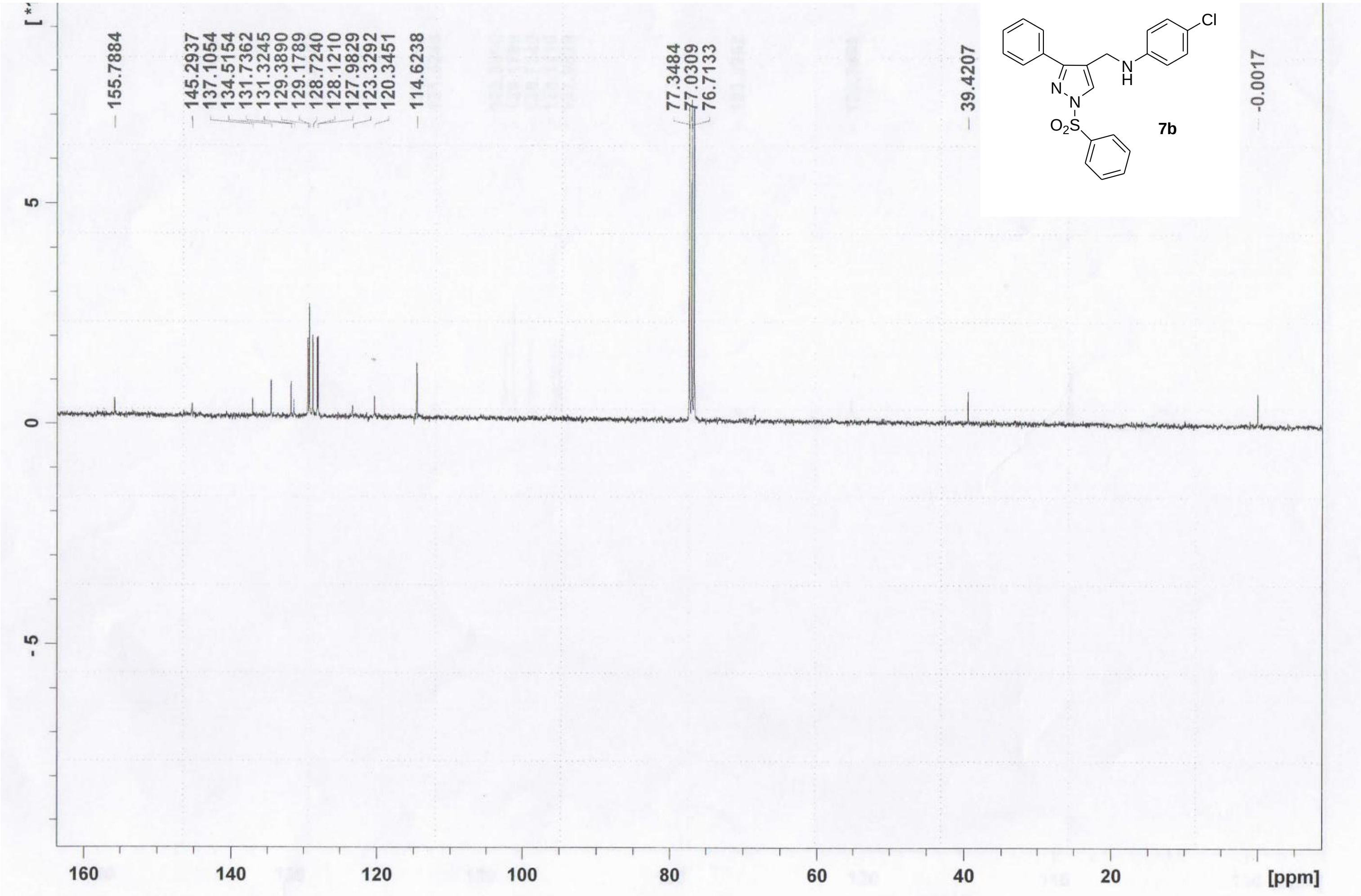

Fig. S 2. ^{13}C NMR spectrum of compound **7b**

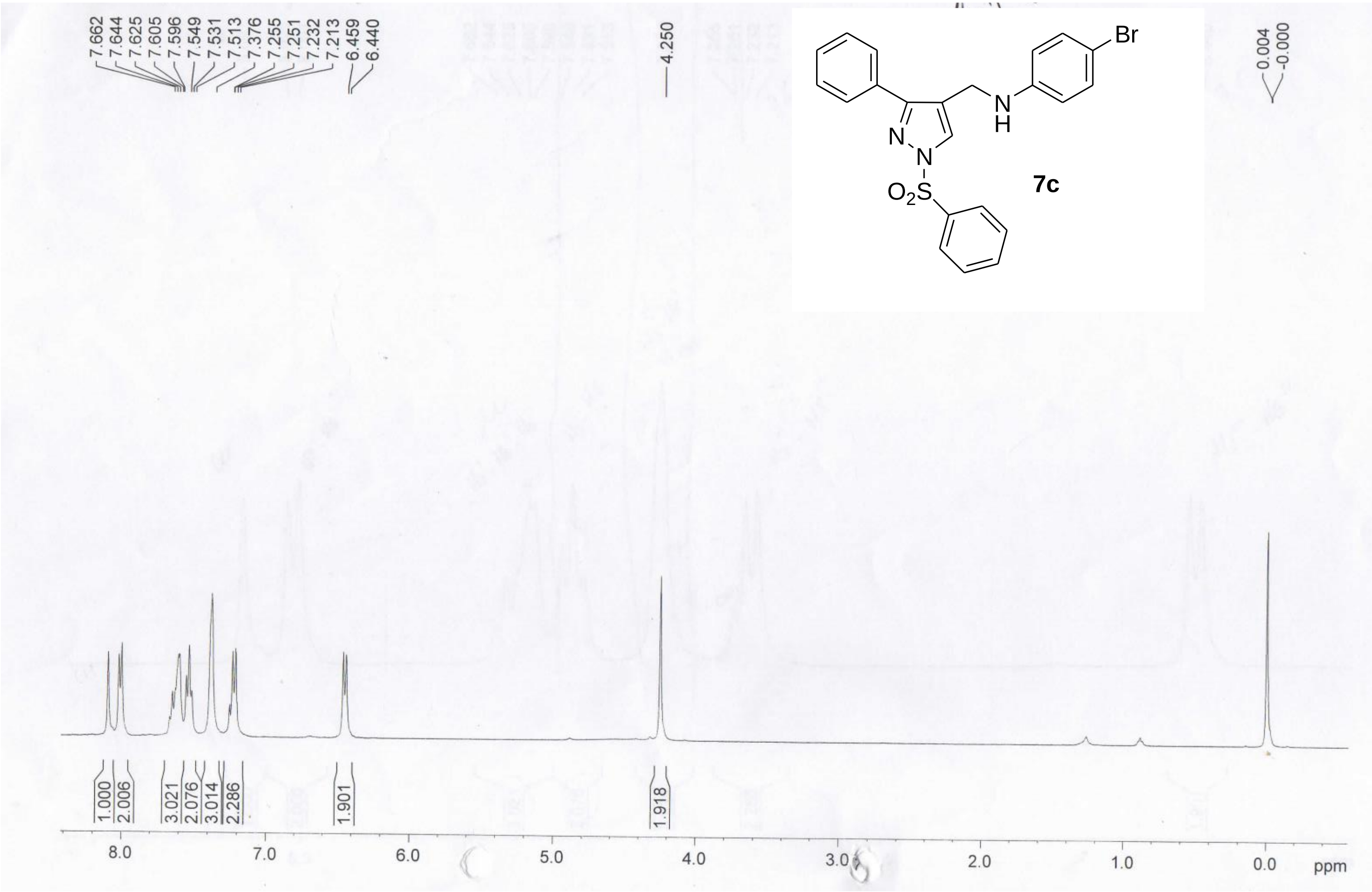

Fig. S 3. ^{1}H NMR spectrum of compound **7c**

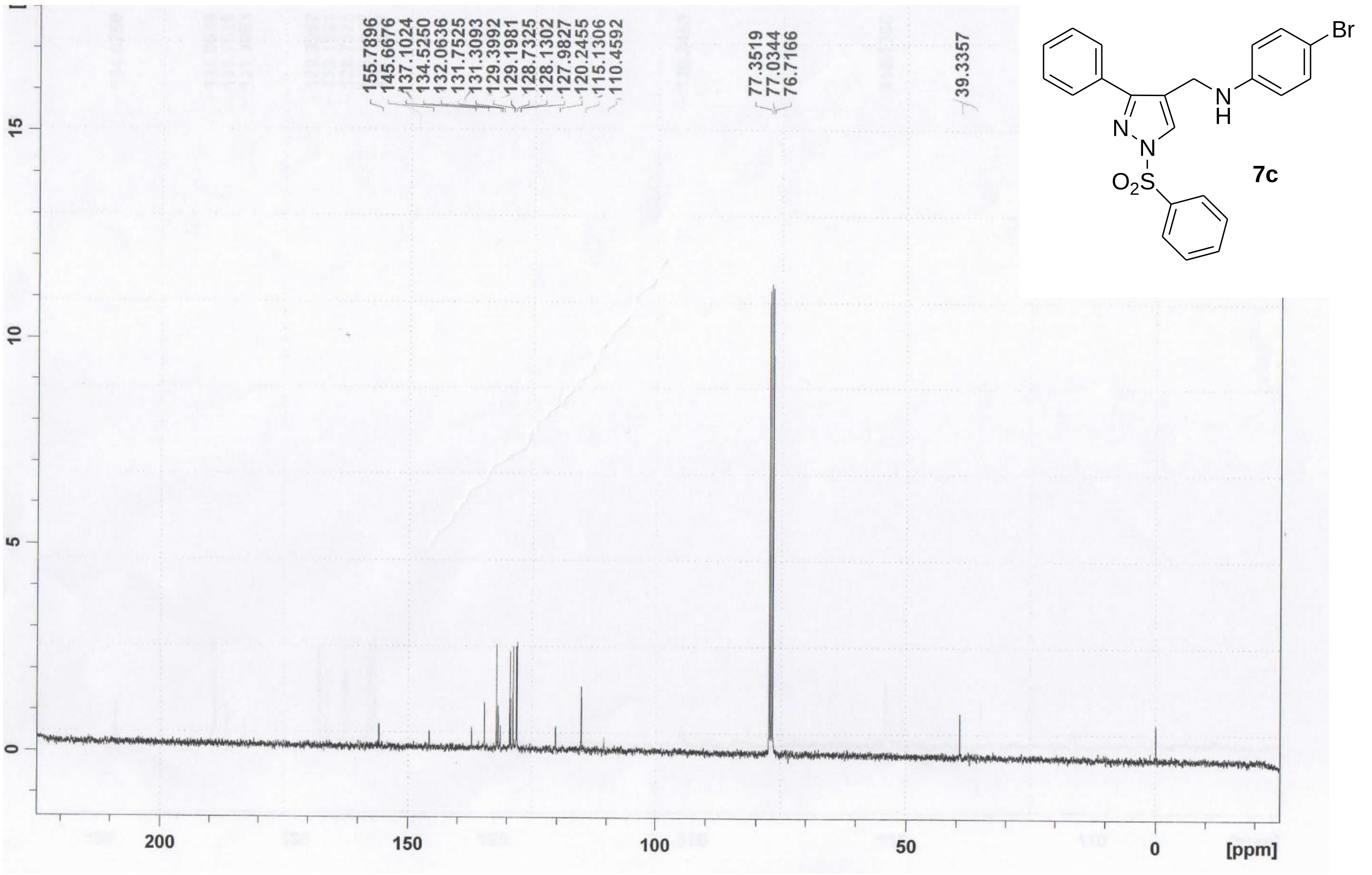

Fig. S 3. ^{13}C NMR spectrum of compound **7c**

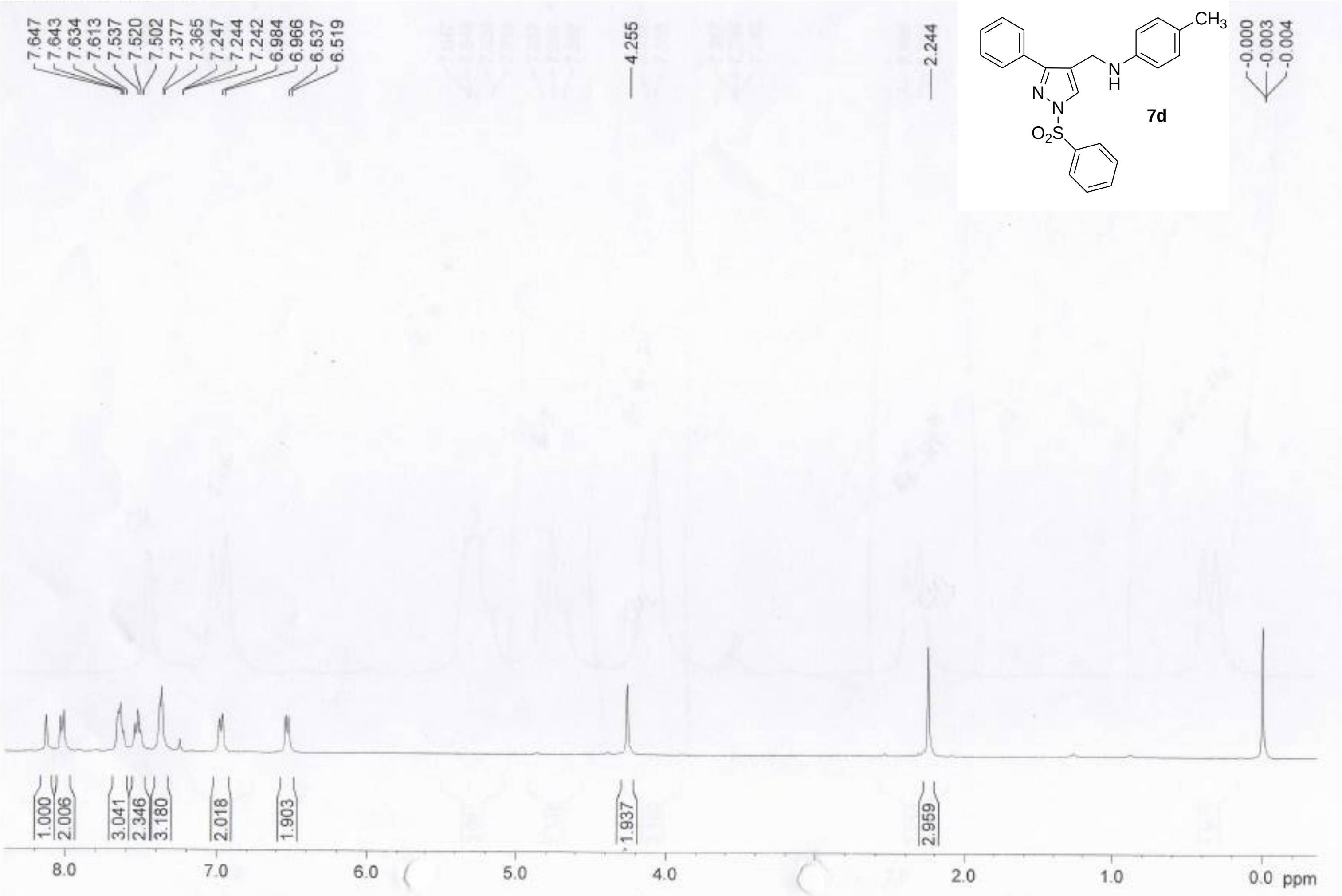

Fig. S 4. ^{1}H NMR of compound **7d**

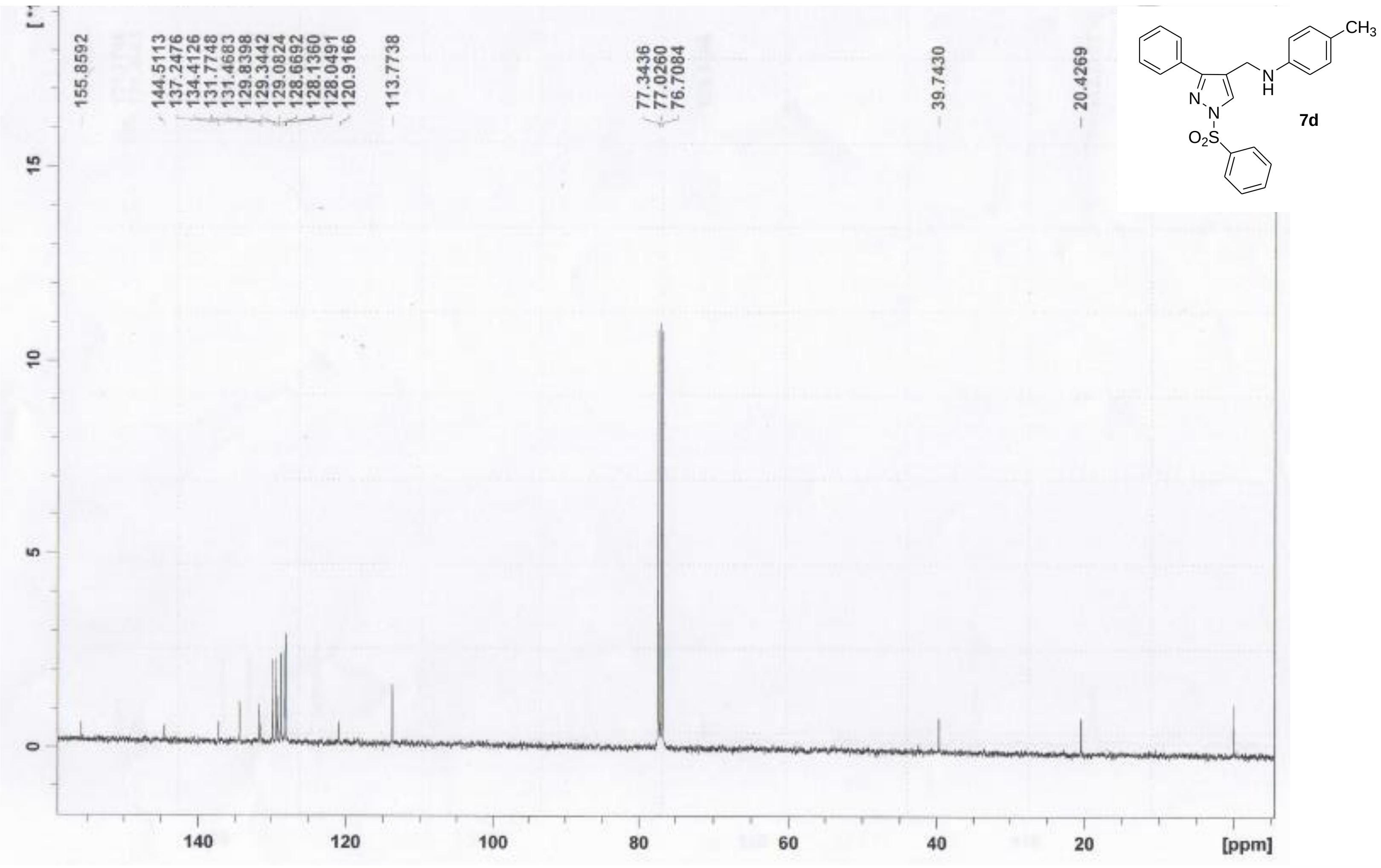

Fig. S 4. ^{13}C NMR of compound **7d**

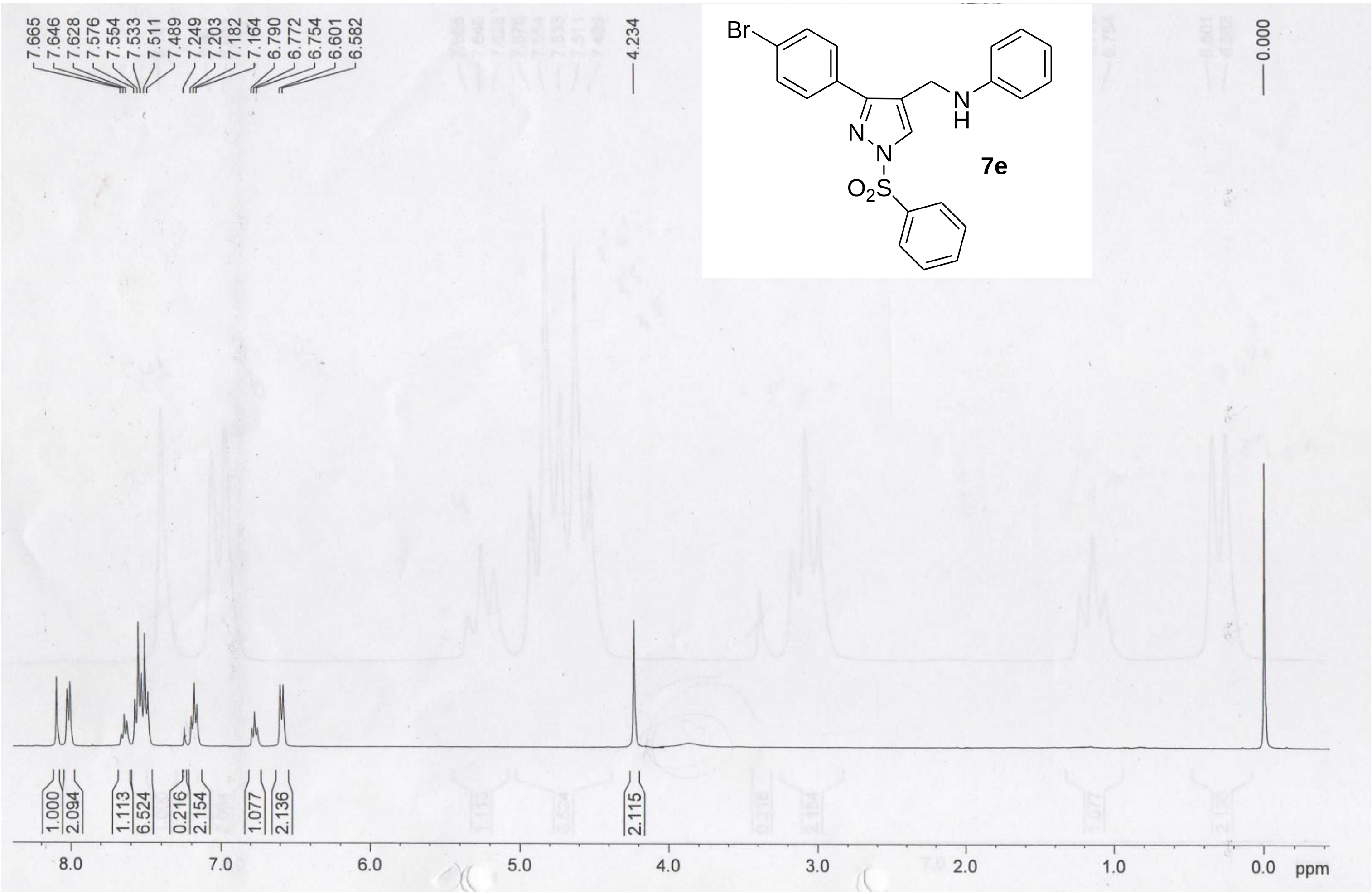

Fig. S5. ^{1}H NMR spectrum of compound **7e**

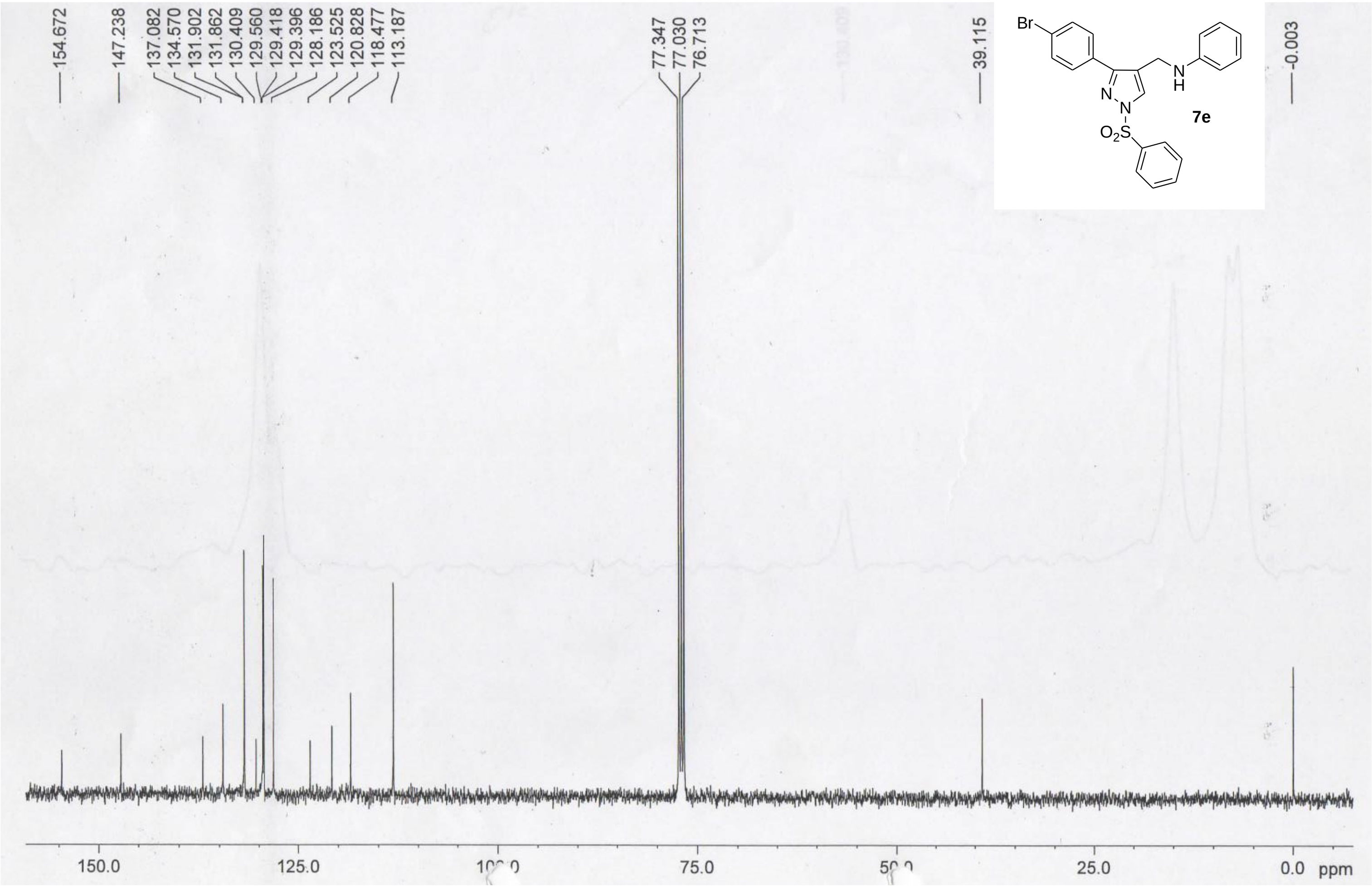

Fig. S5. ^{13}C NMR spectrum of compound **7e**

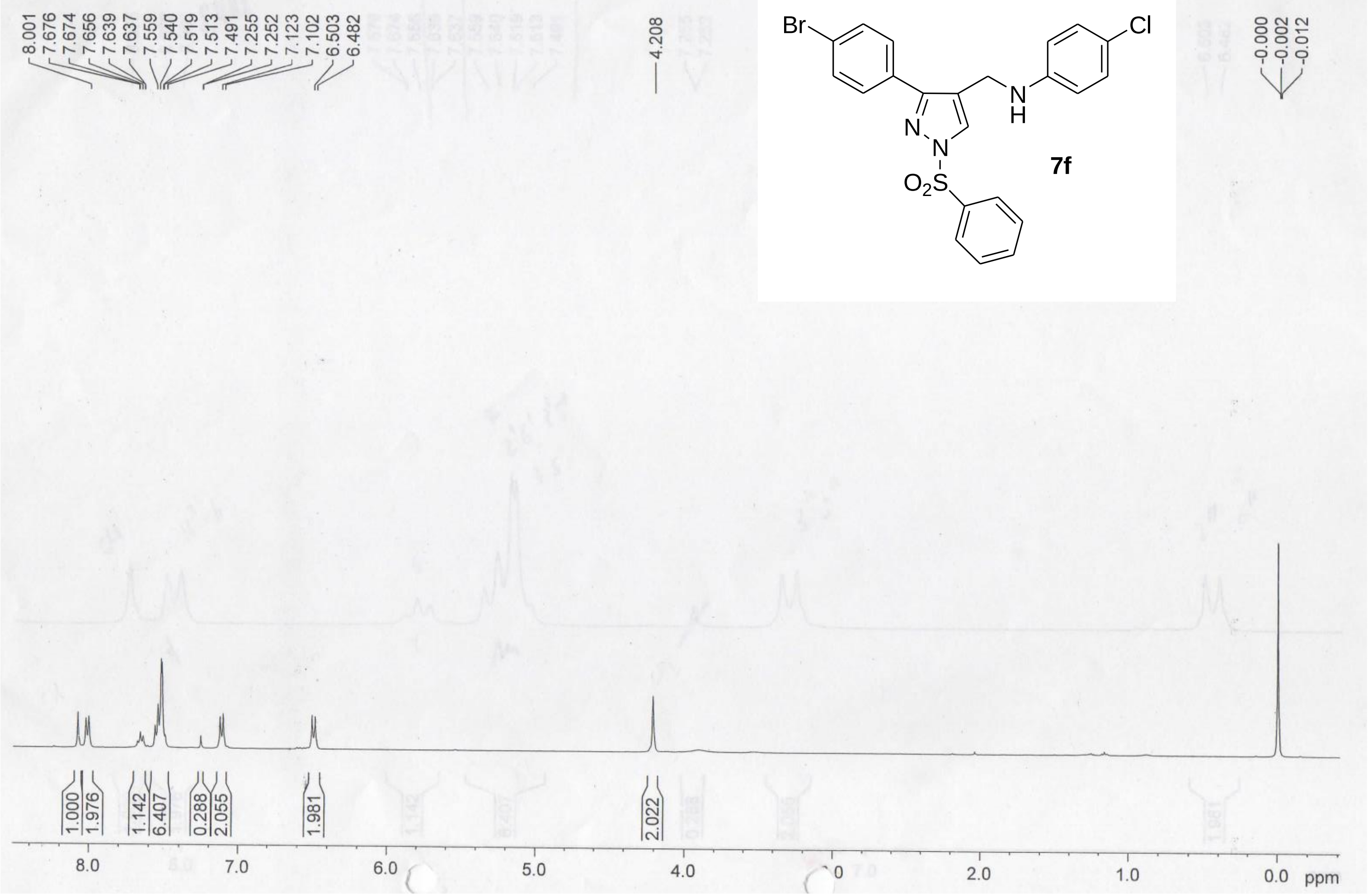

Fig. S6. ^{1}H NMR spectrum of compound **7f**

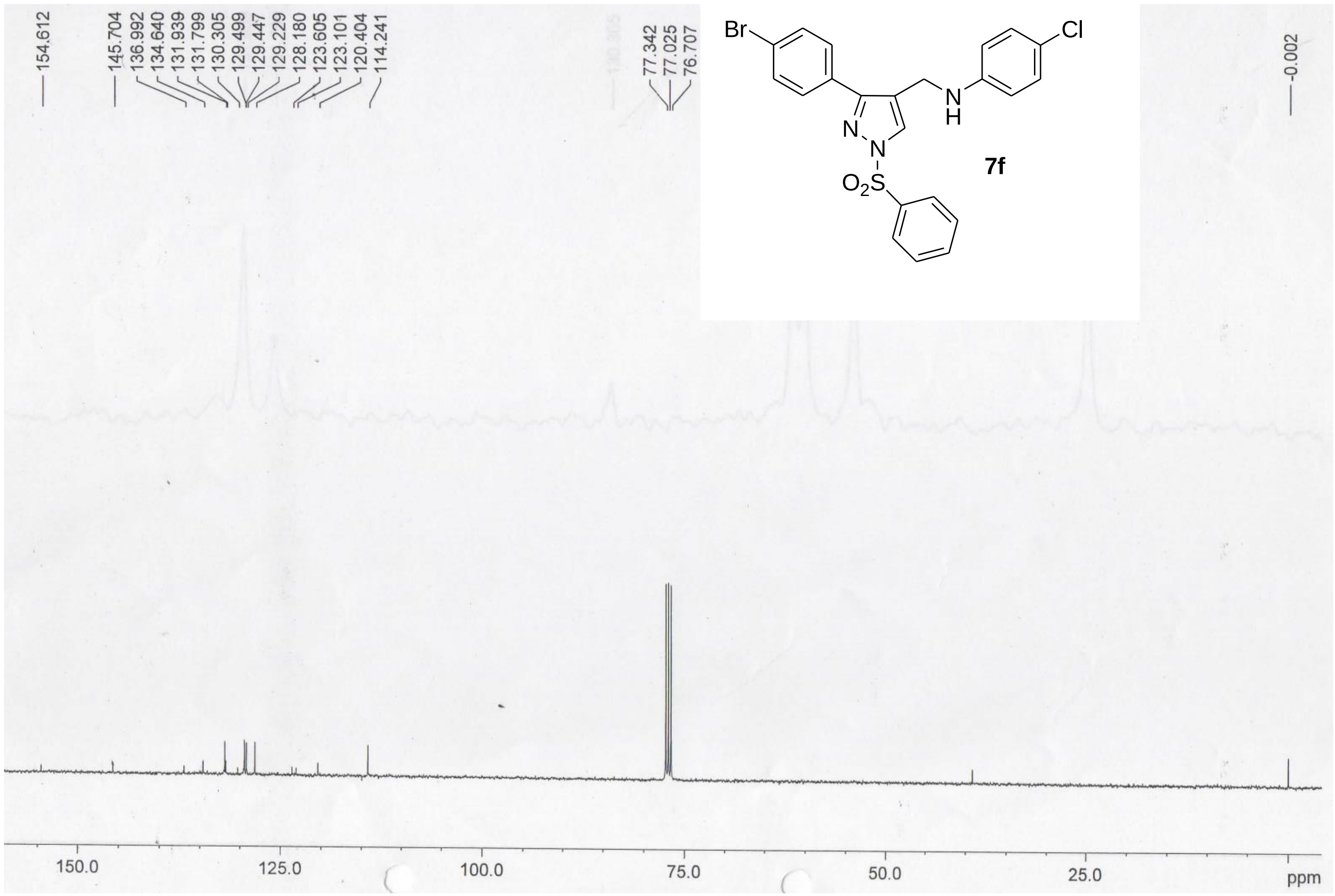

Fig. S6. ^{13}C NMR spectrum of compound **7f**

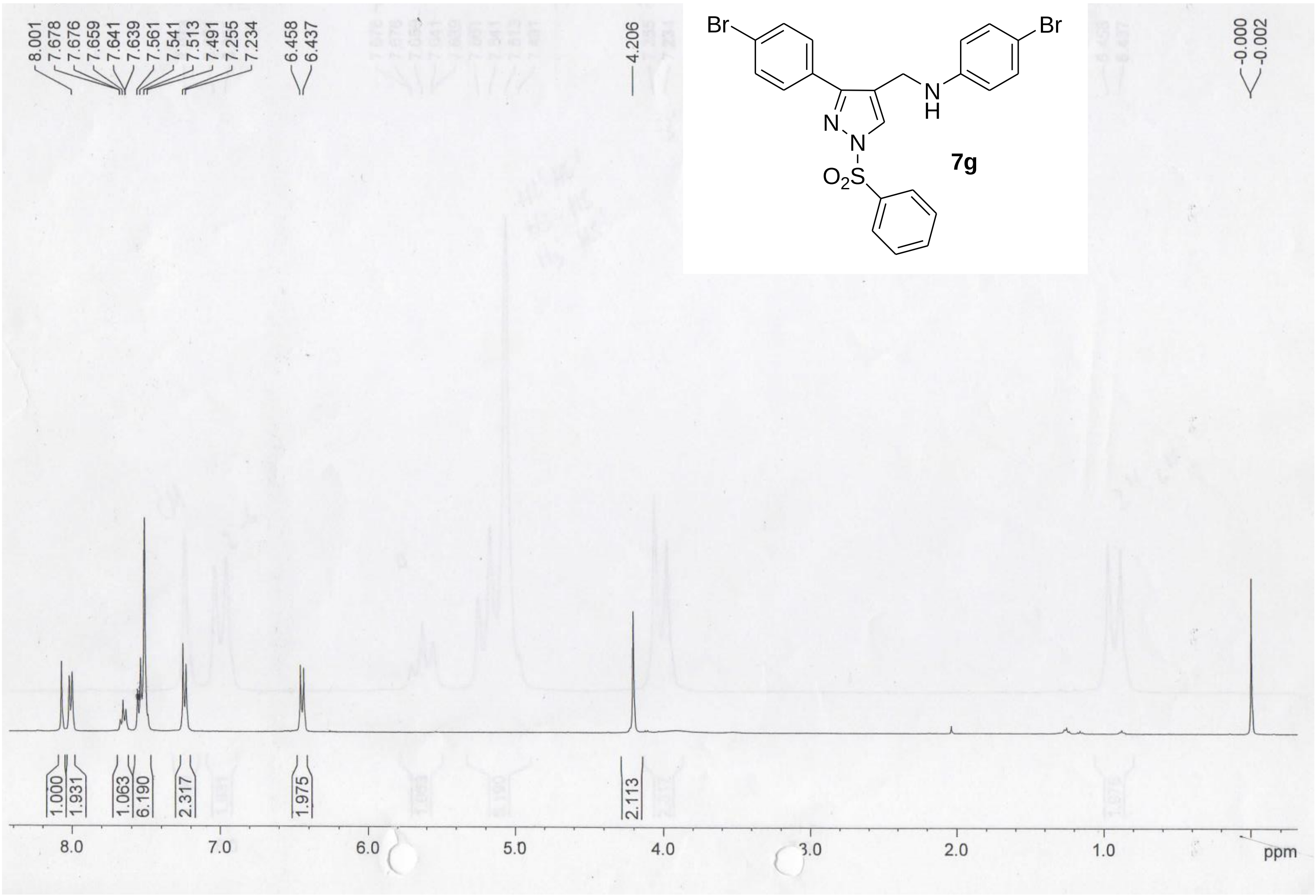

Fig. S7. ^{1}H NMR spectrum of compound **7g**

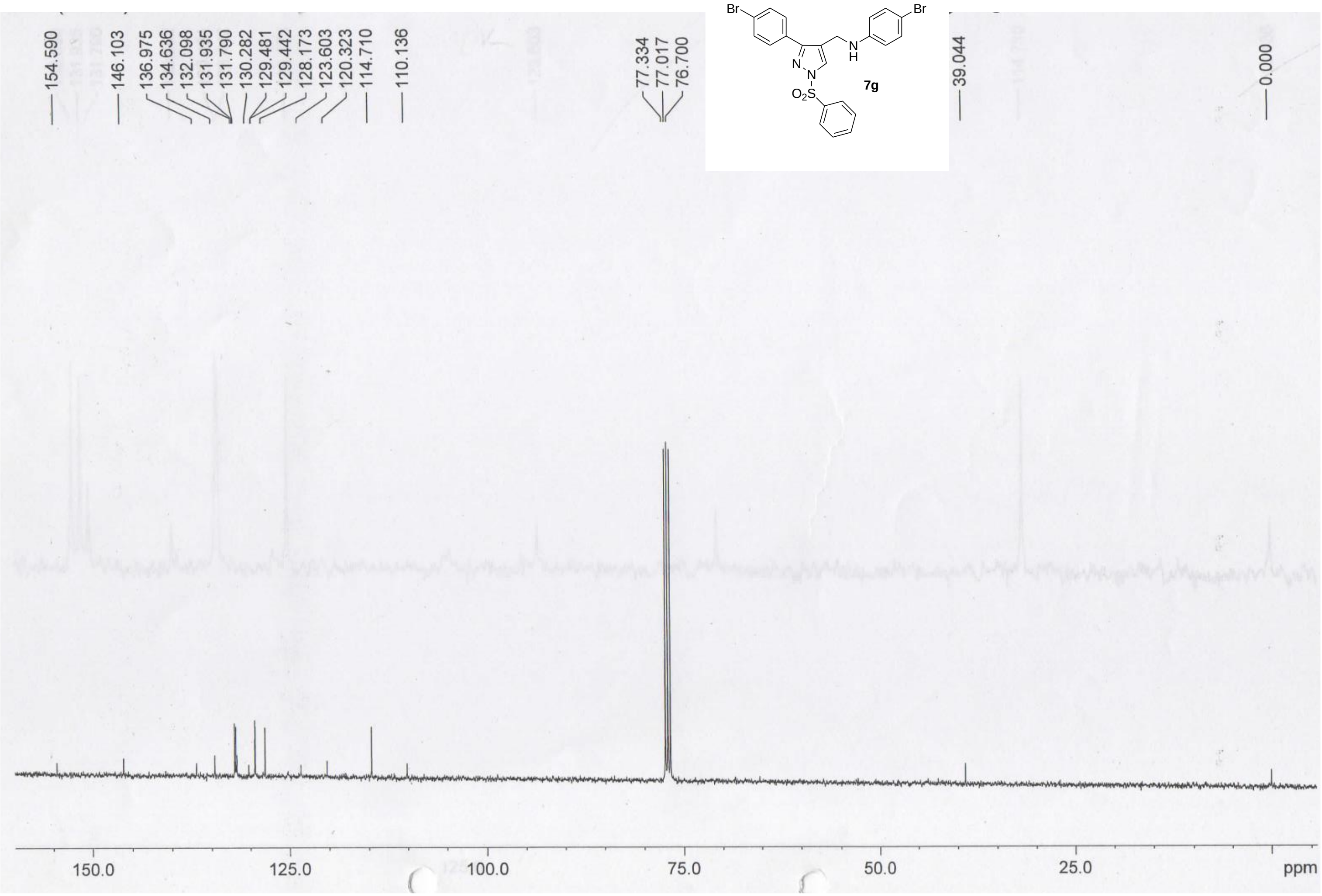

Fig. S7. ^{13}C NMR spectrum of compound **7g**

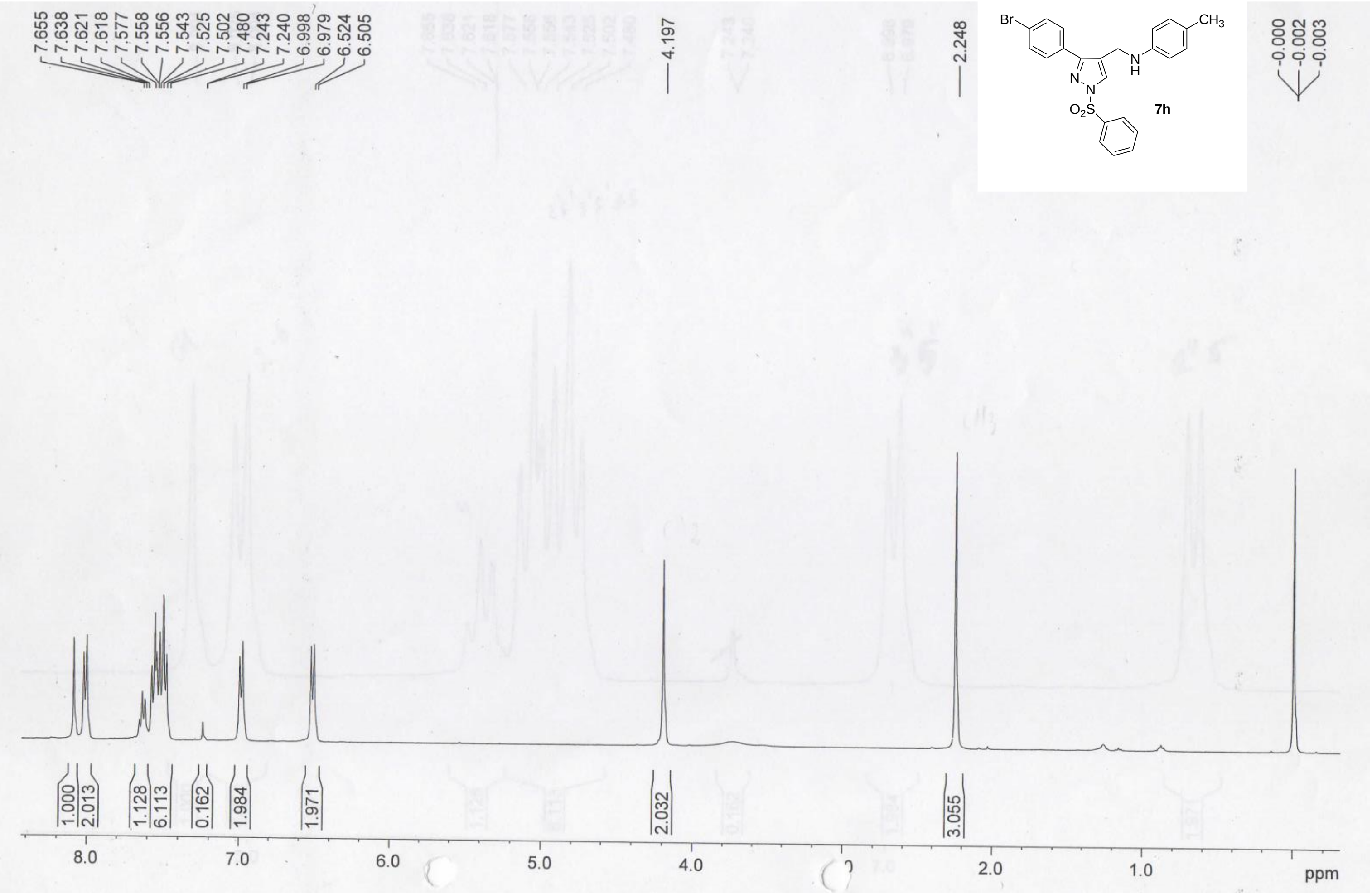

Fig. S8. ^{1}H NMR spectrum of compound **7h**

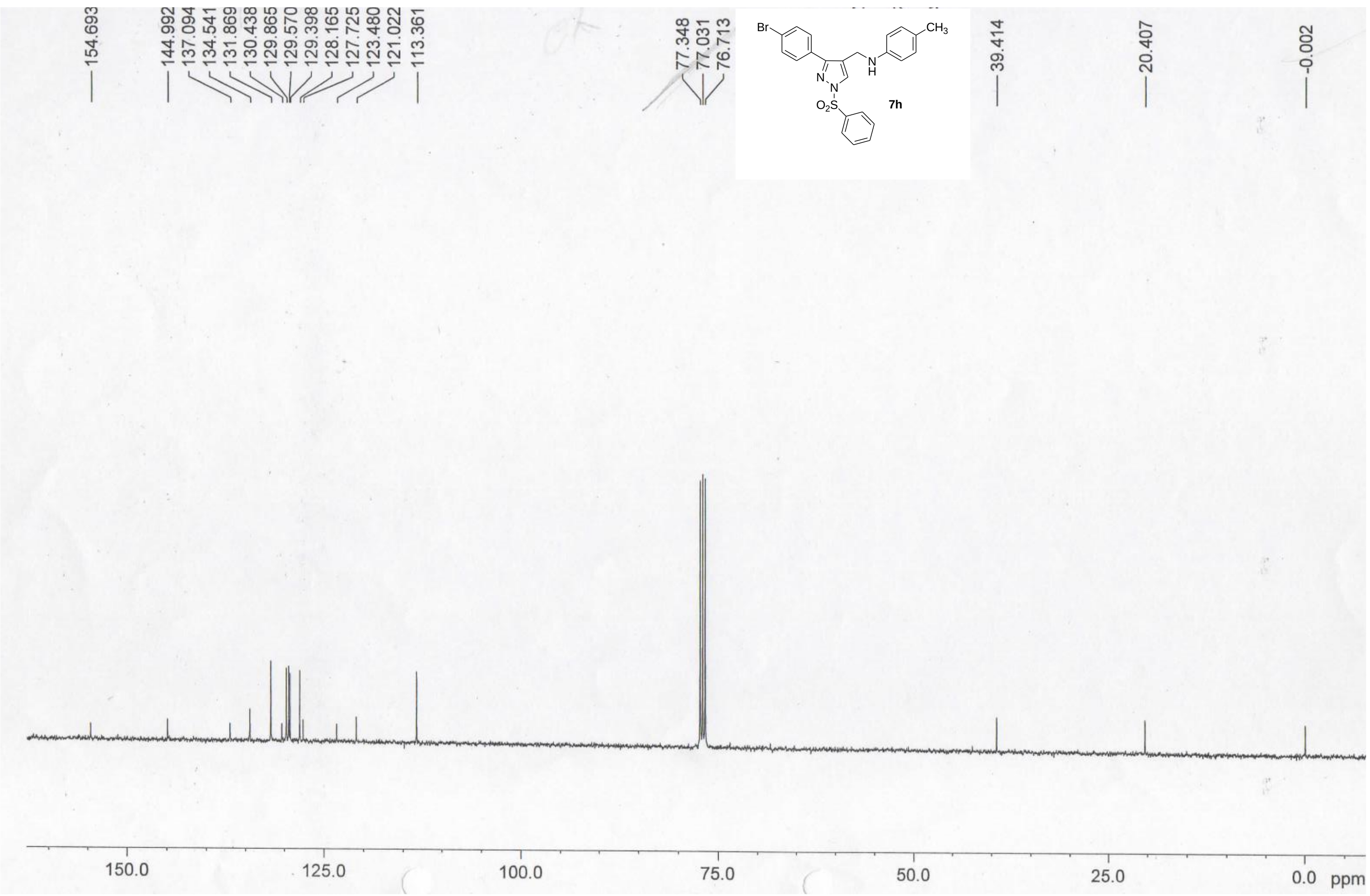

Fig. S8. ^{13}C NMR spectrum of compound **7h**

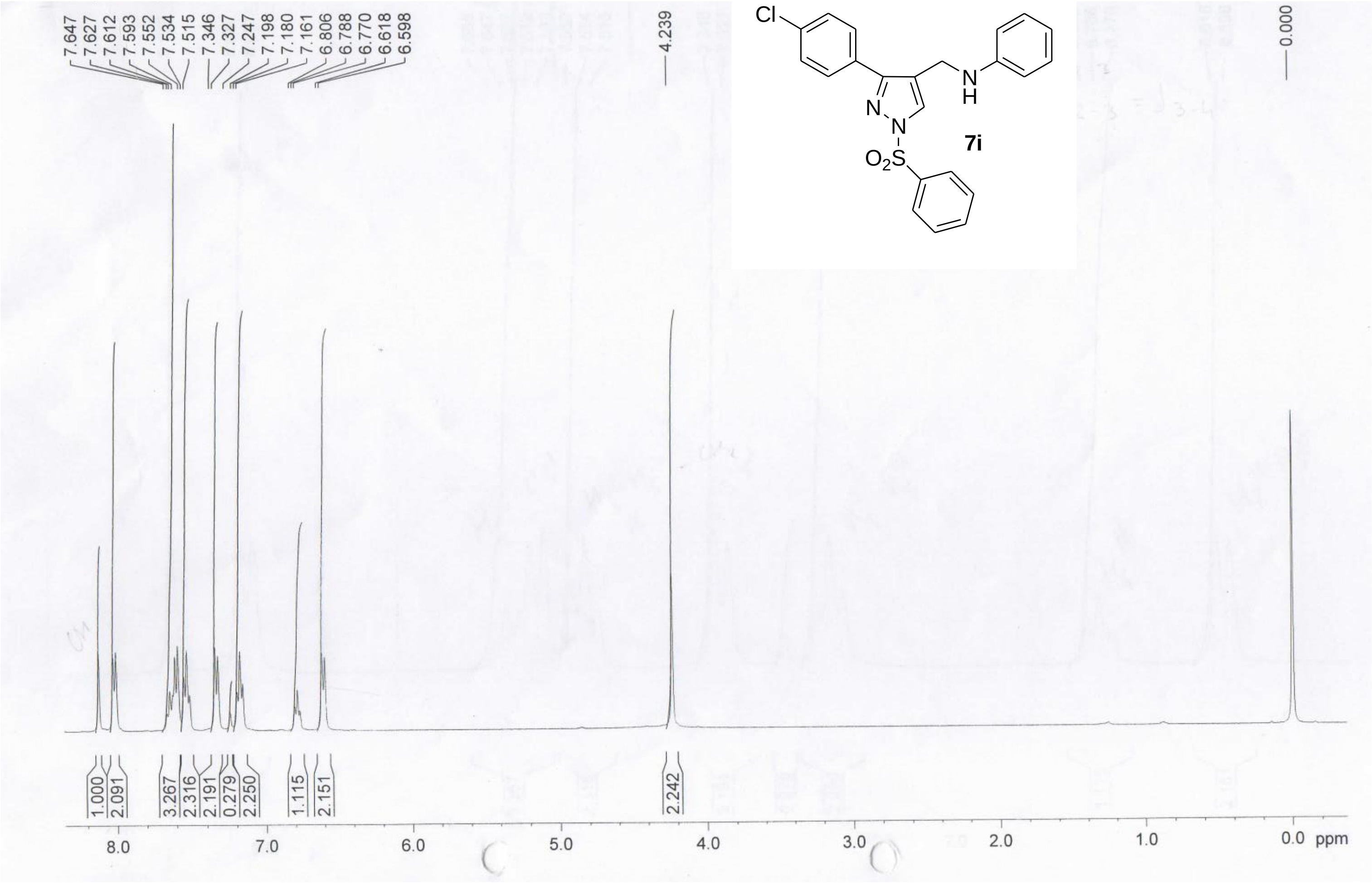

Fig. S9. ^{1}H NMR spectrum of compound **7i**

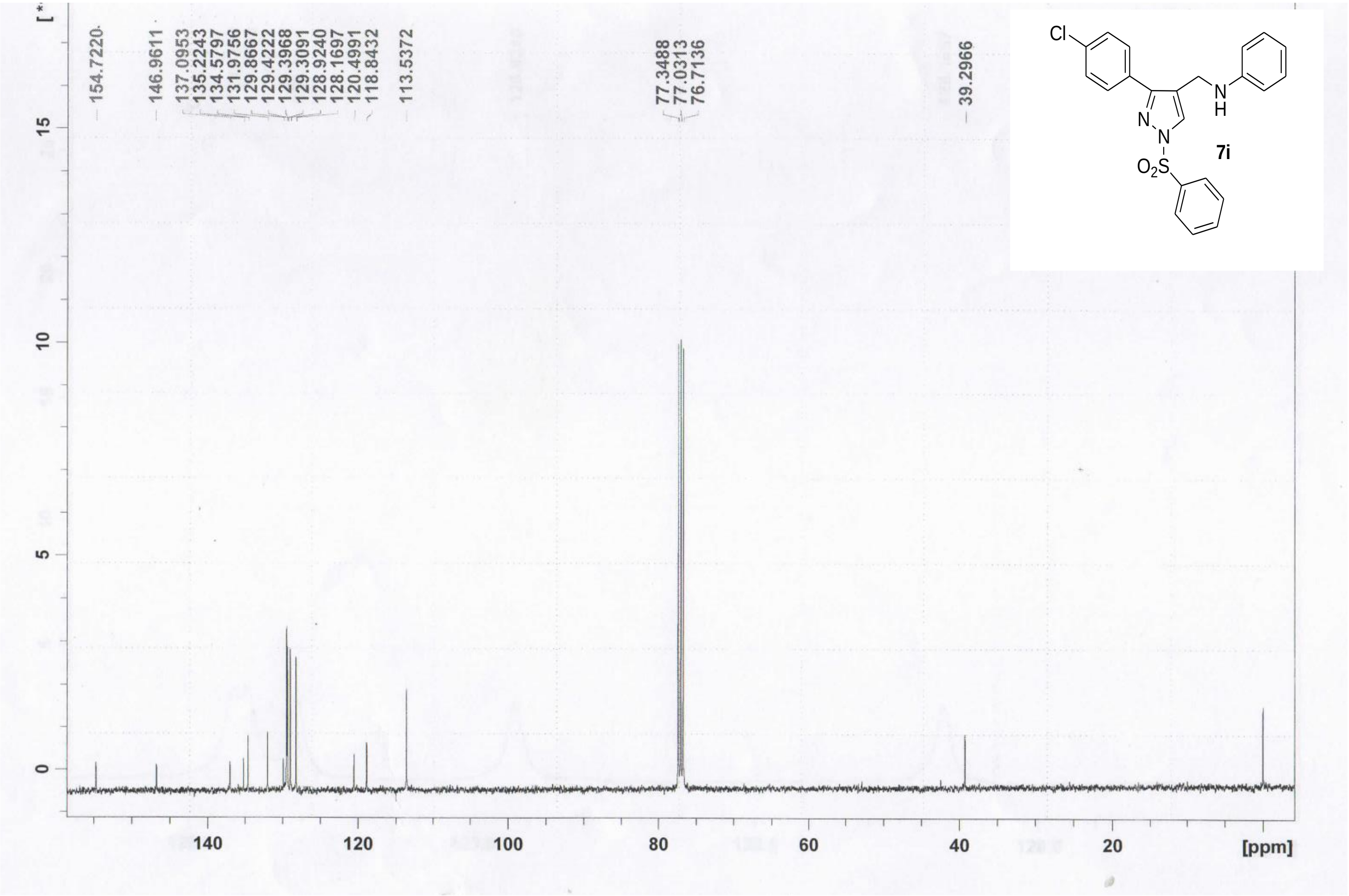

Fig. S9. ^{13}C NMR spectrum of compound **7i**

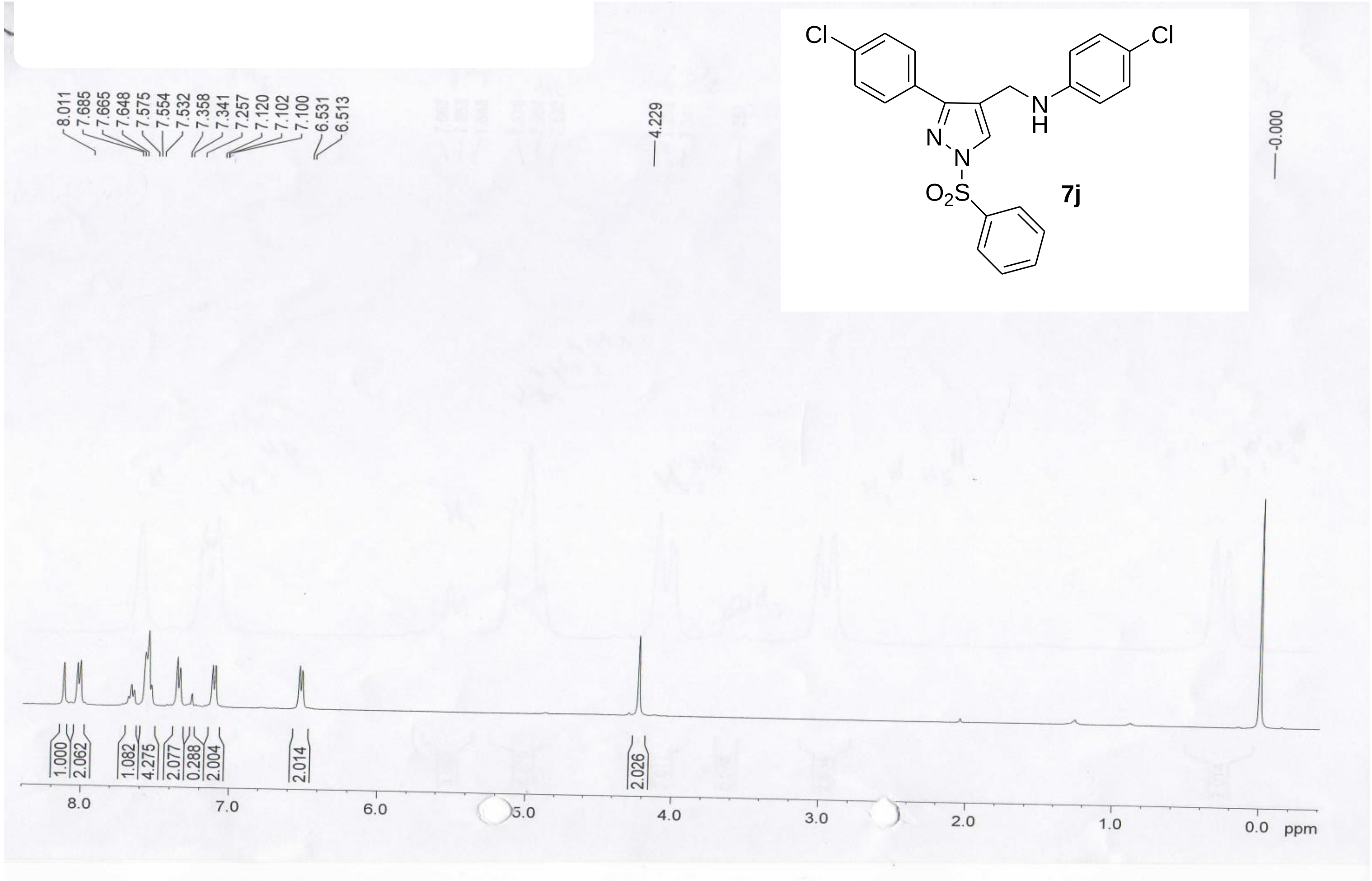

Fig S 10. ^{1}H NMR spectrum of compound **7j**

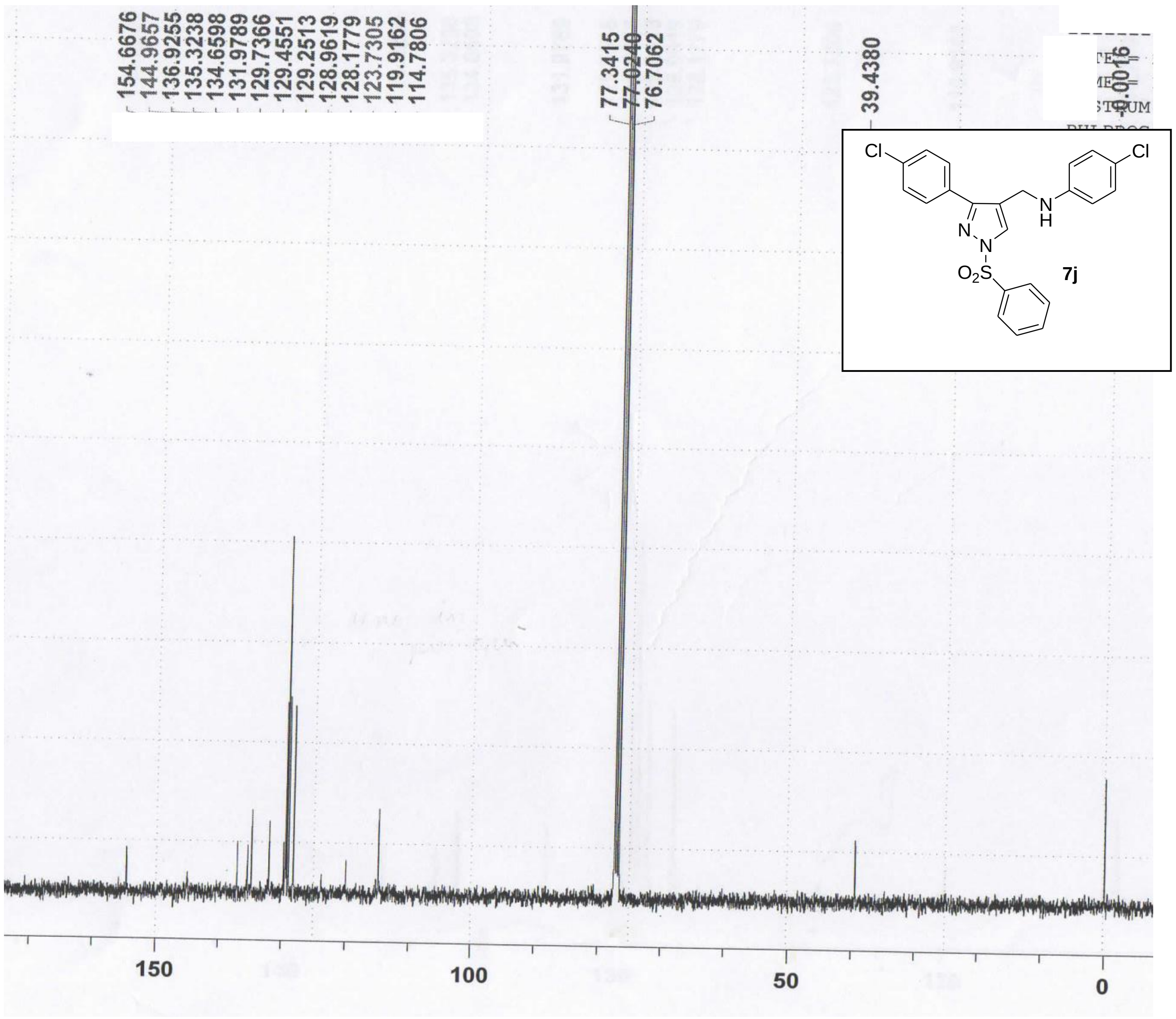

Fig S 10. ^{13}C NMR spectrum of compound **7j**

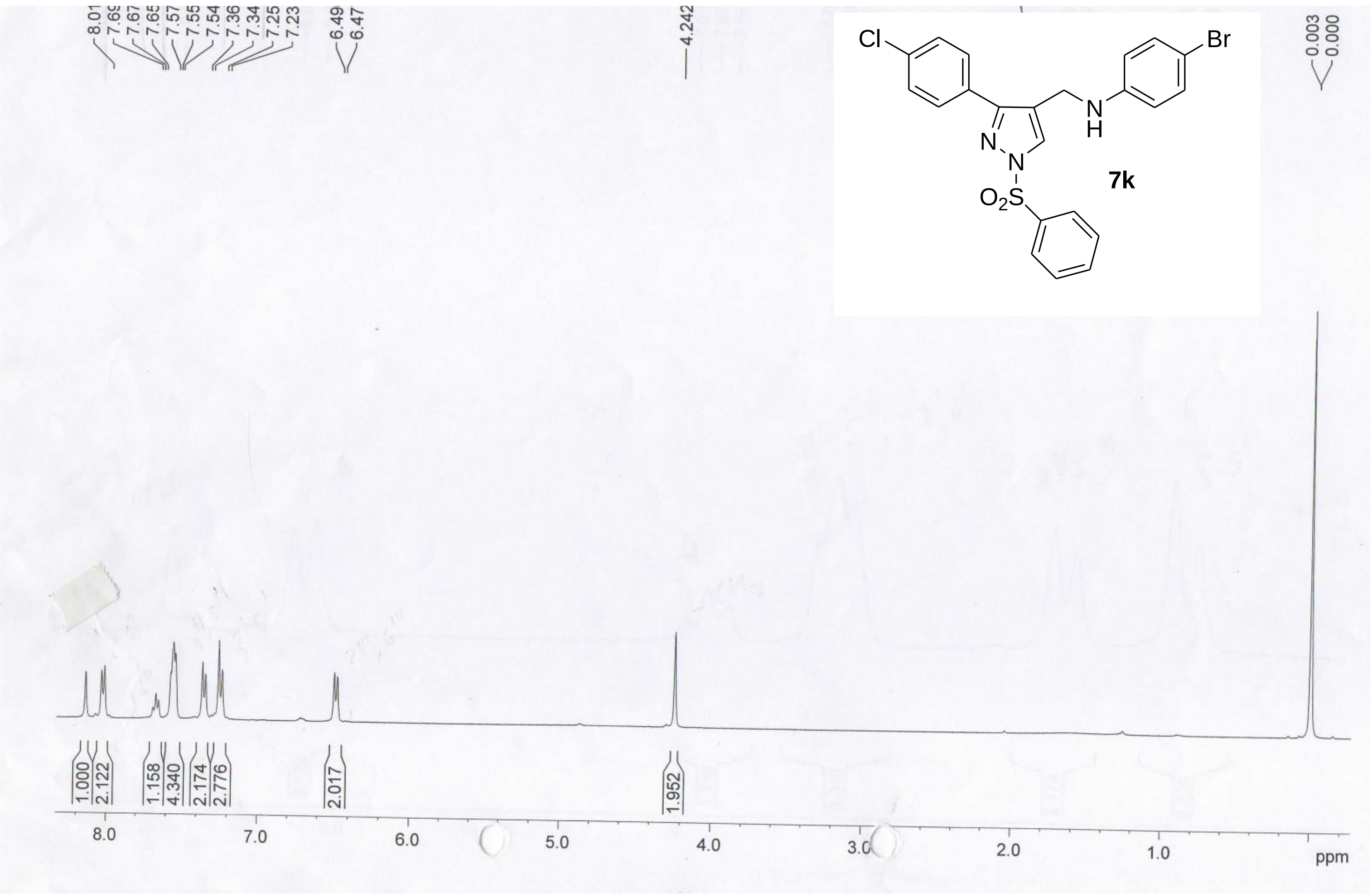

Fig. S 11. ^{1}H NMR spectrum of compound **7k**

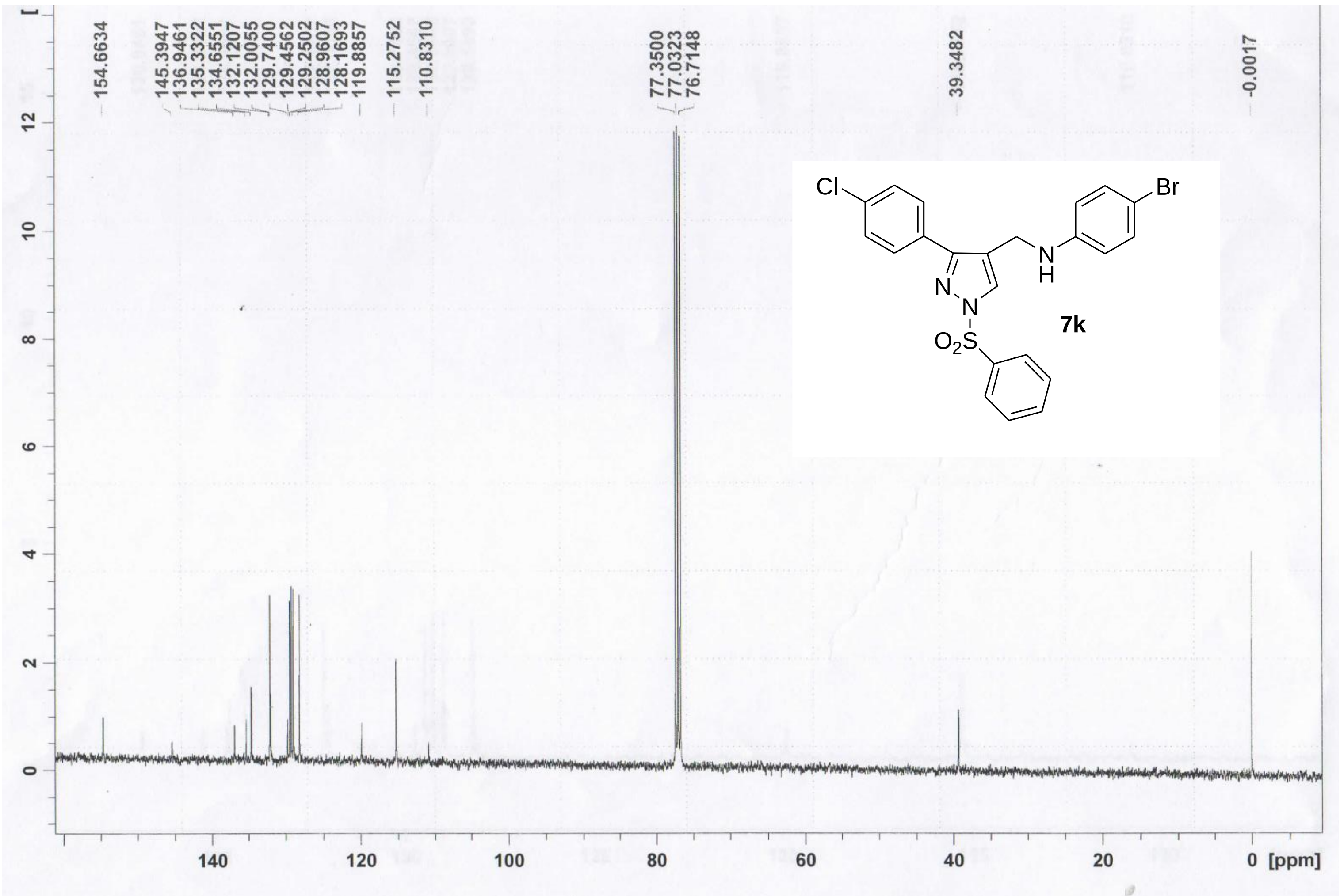

Fig. S 11. ^{13}C NMR spectrum of compound **7k**

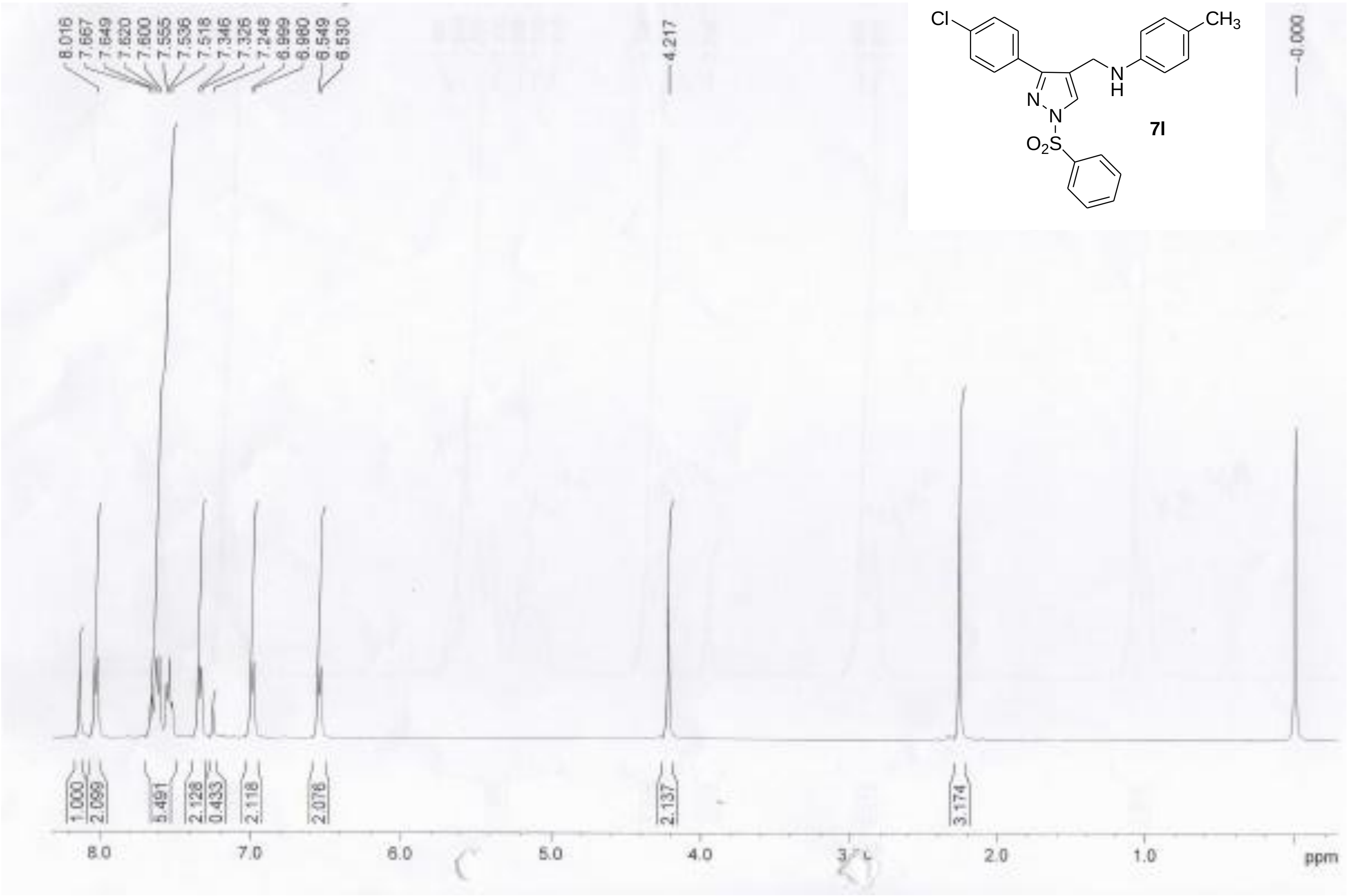

Fig. S 12. ^{1}H NMR spectrum of compound **7l**

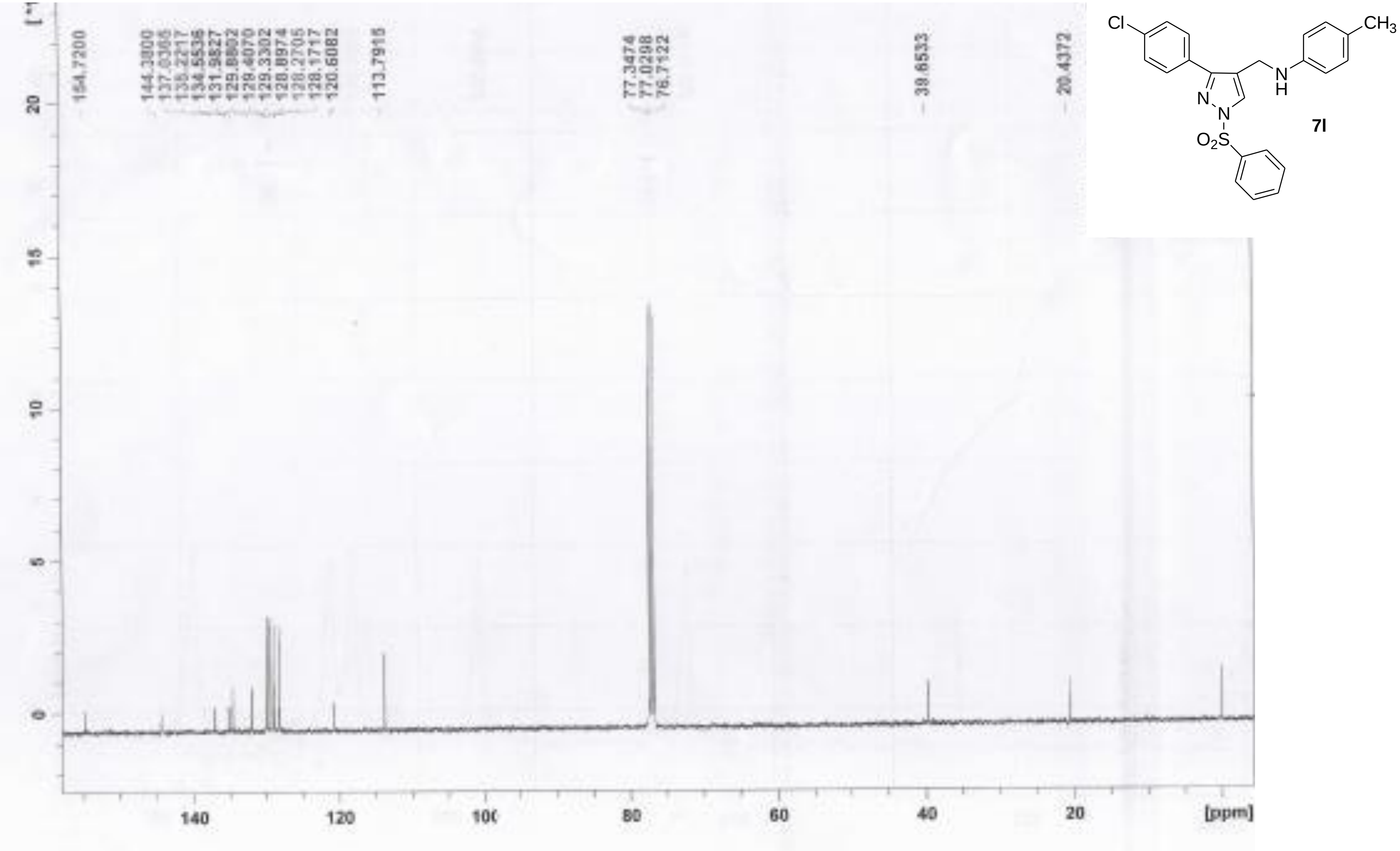

Fig. S 12. ^{13}C NMR spectrum of compound **7l**

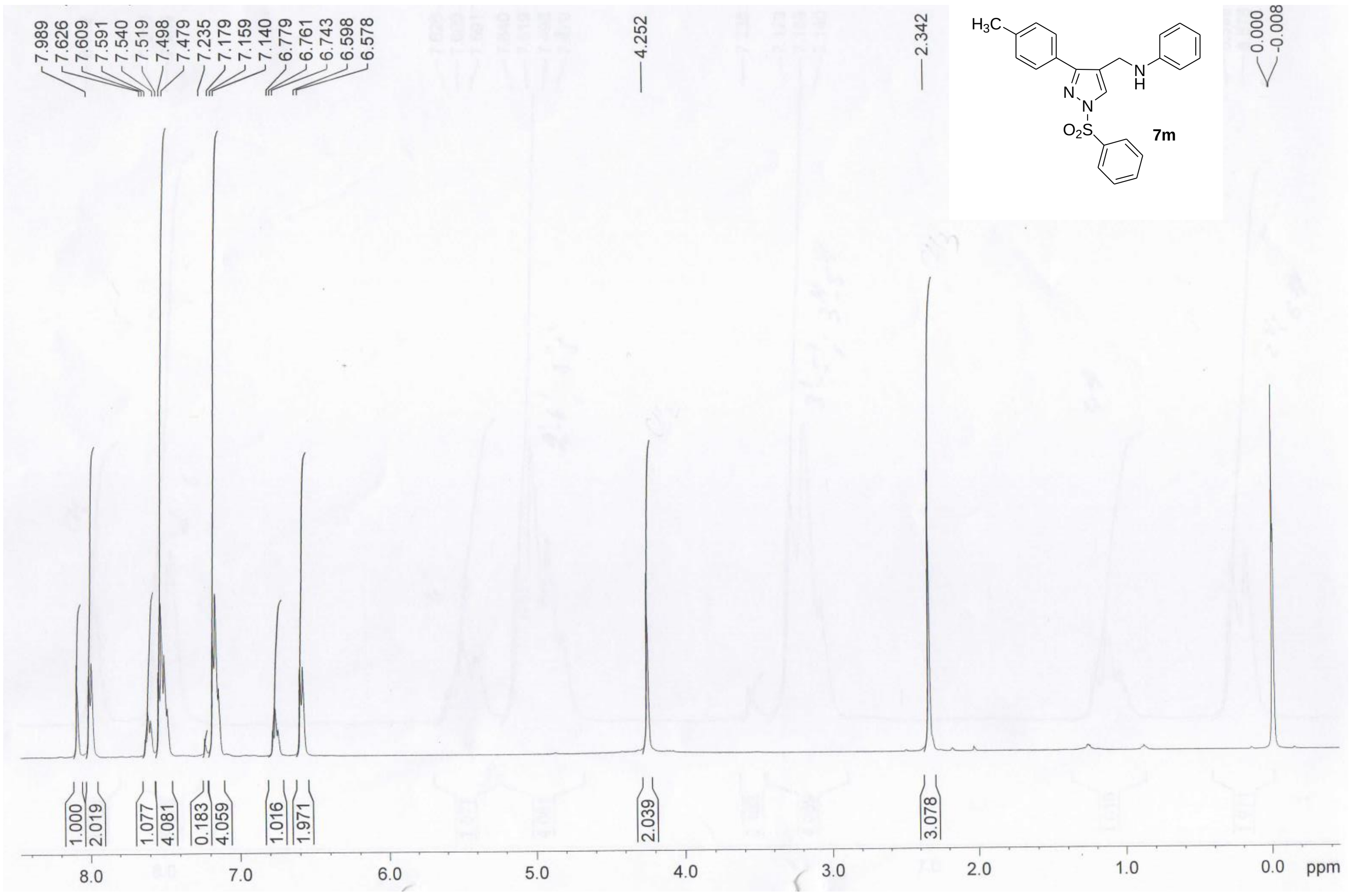

Fig. S 13. ^{1}H NMR spectrum of compound **7m**

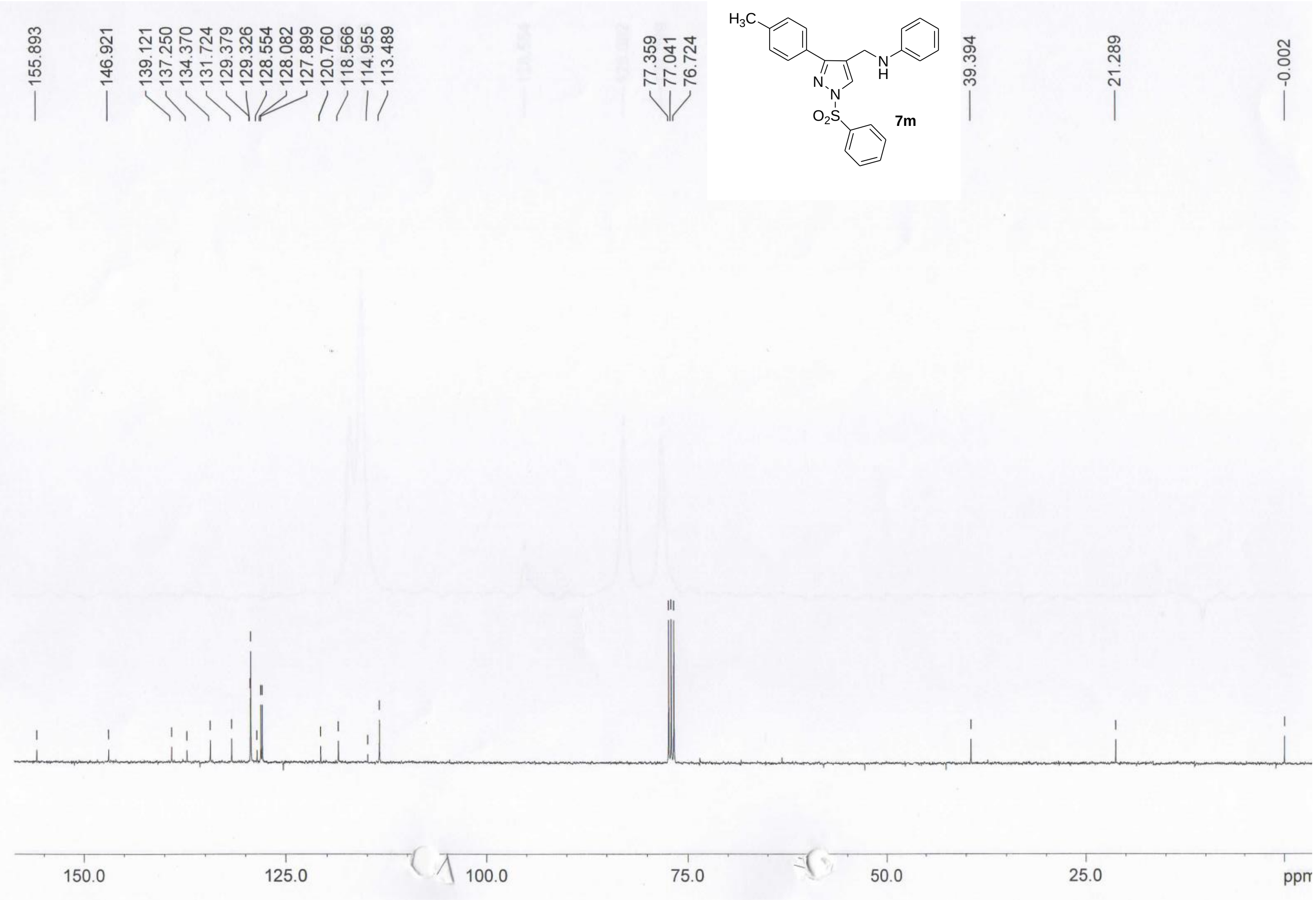

Fig. S 13. ^{13}C NMR spectrum of compound **7m**

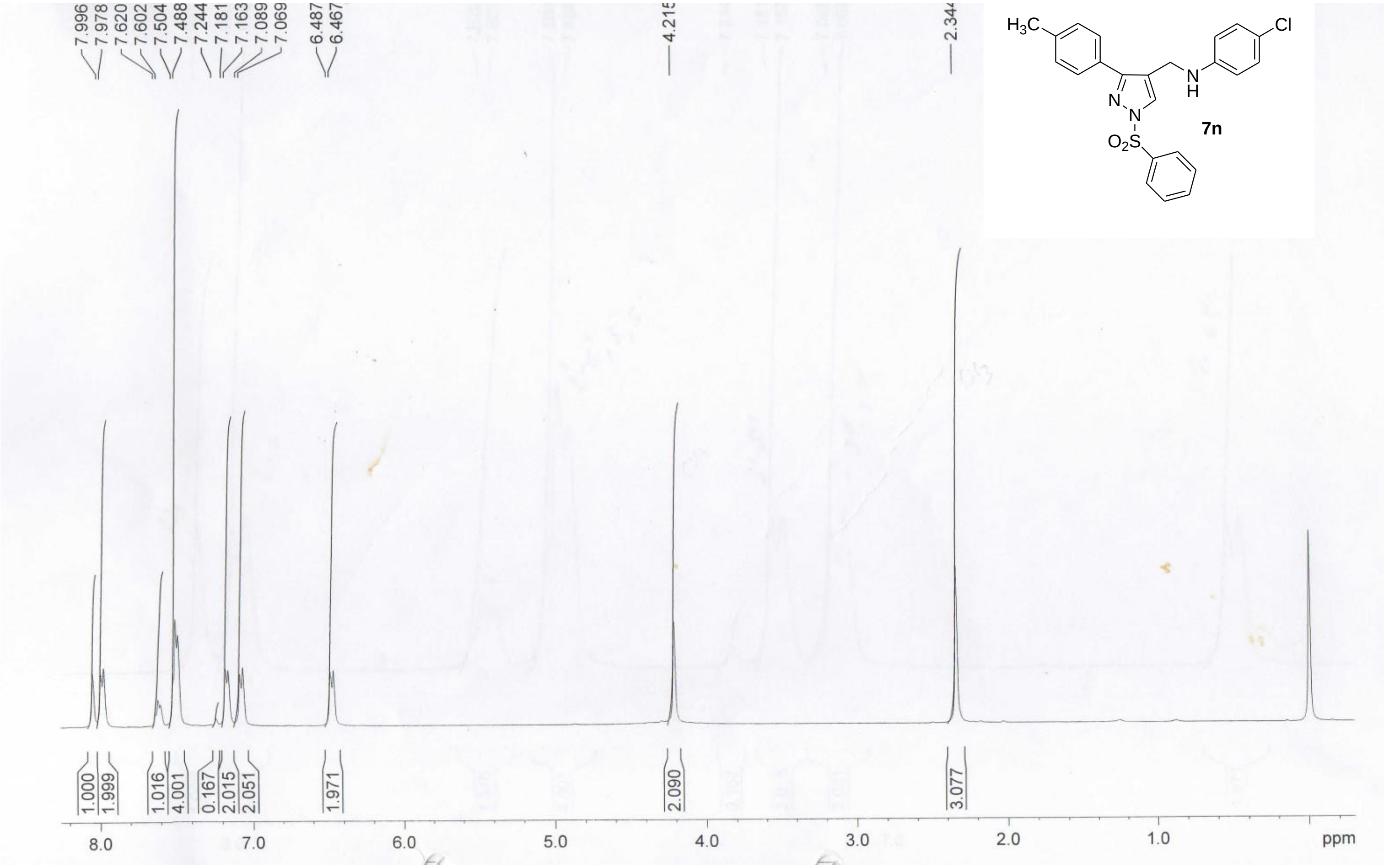

Fig. S 14. ^{1}H NMR spectrum of compound **7n**

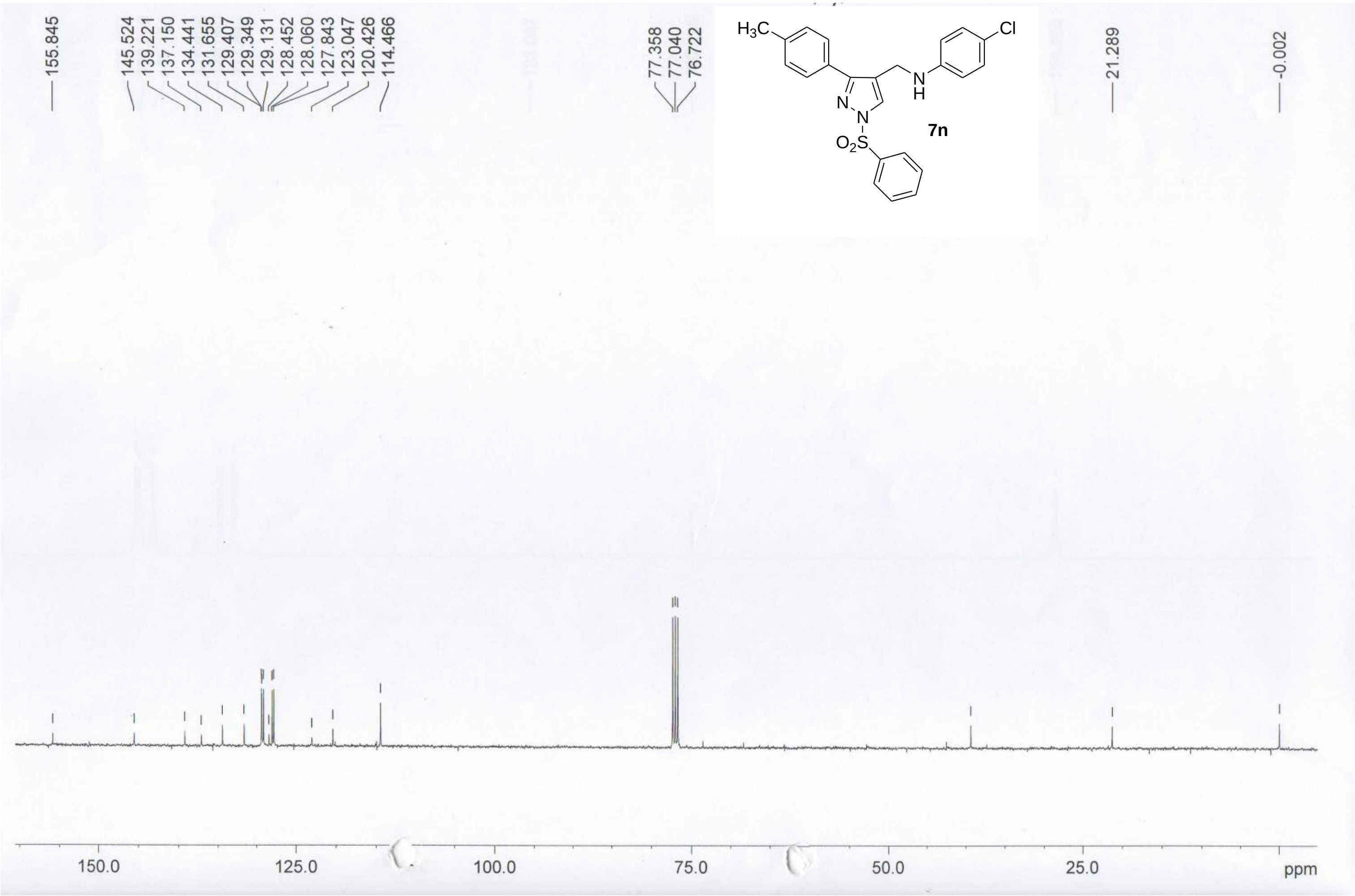

Fig. S 14. ^{13}C NMR spectrum of compound **7n**

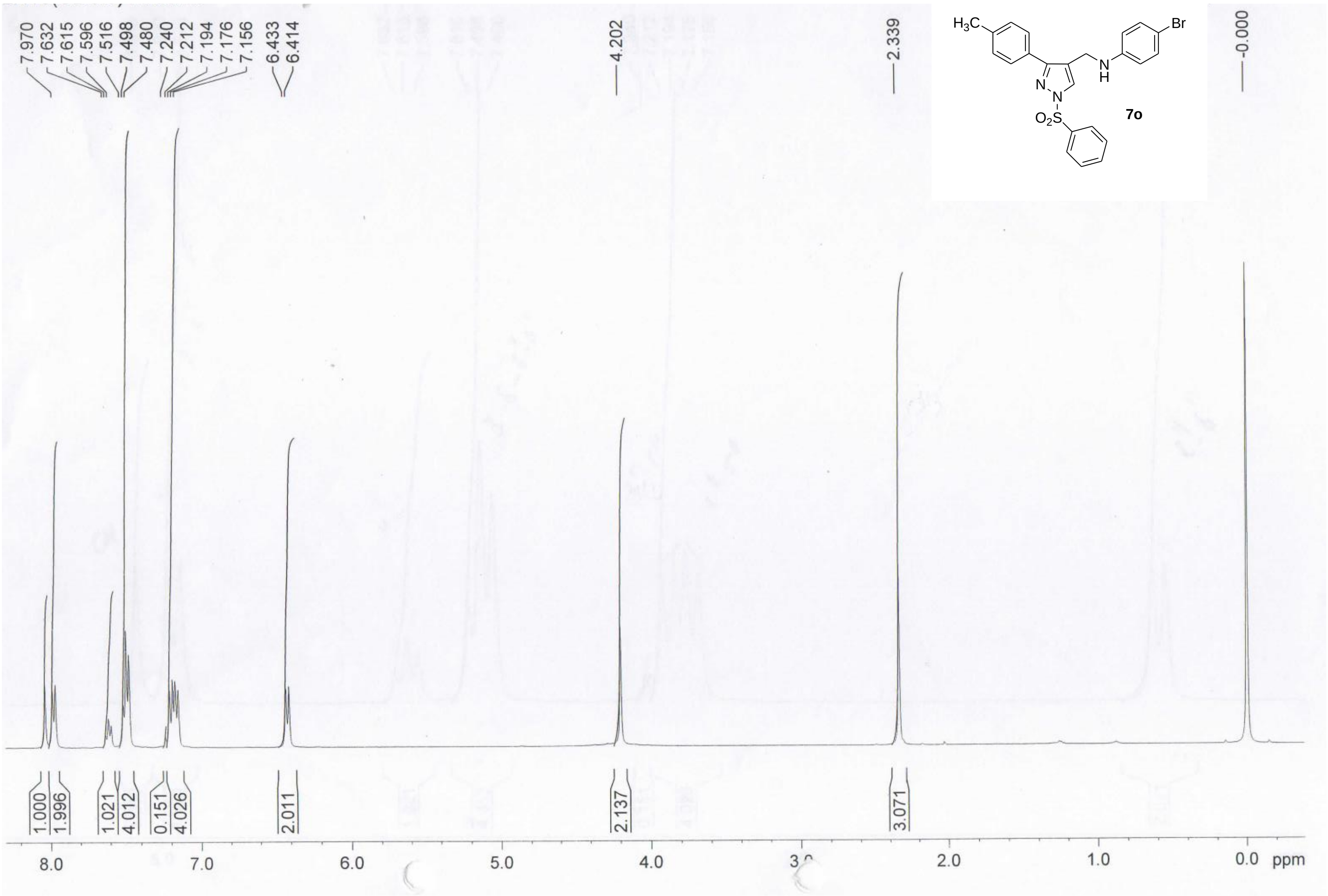

Fig. S 15. ^{1}H NMR spectrum of compound **7o**

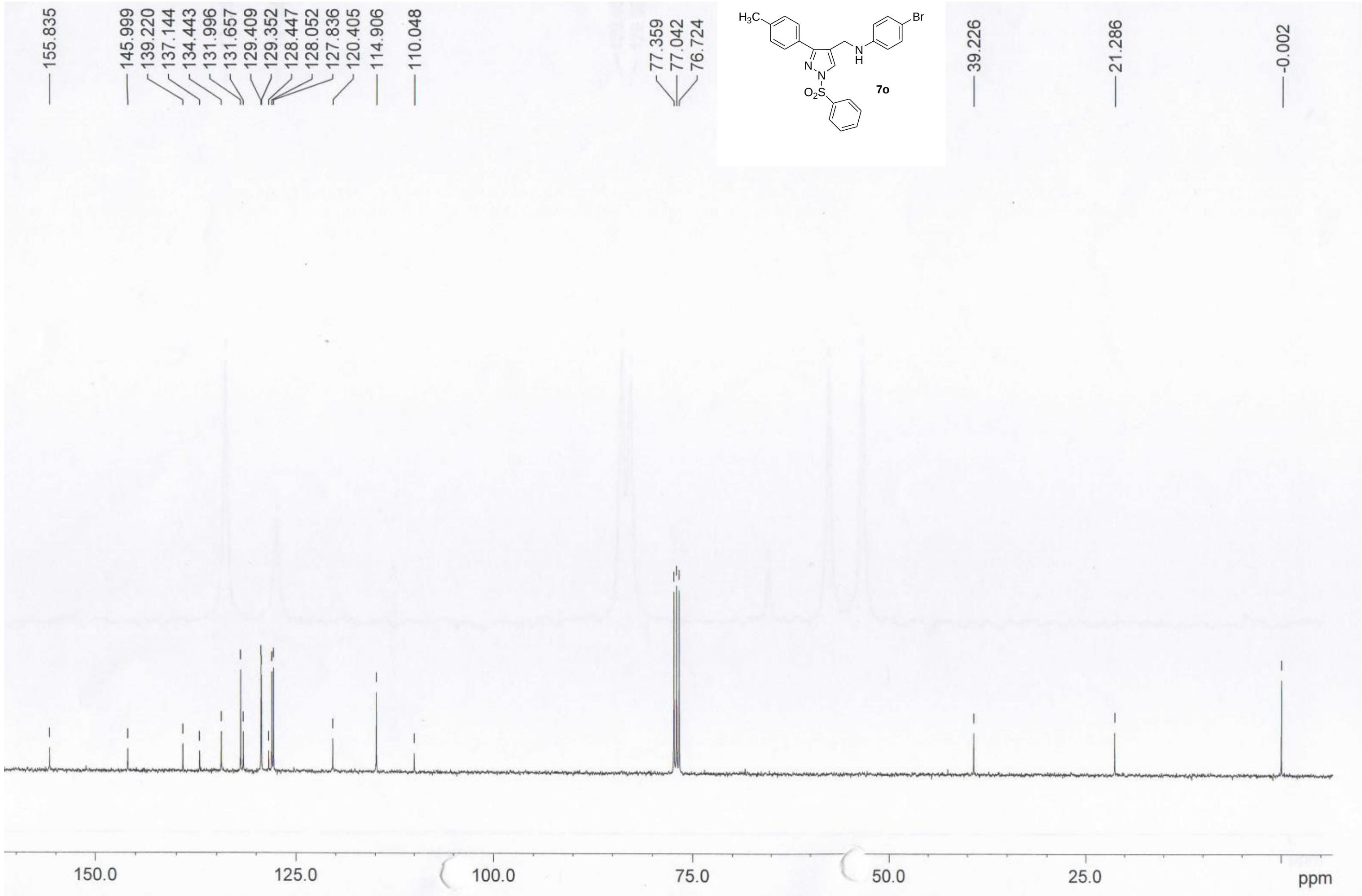

Fig. S 15. ^{13}C NMR spectrum of compound **7o**

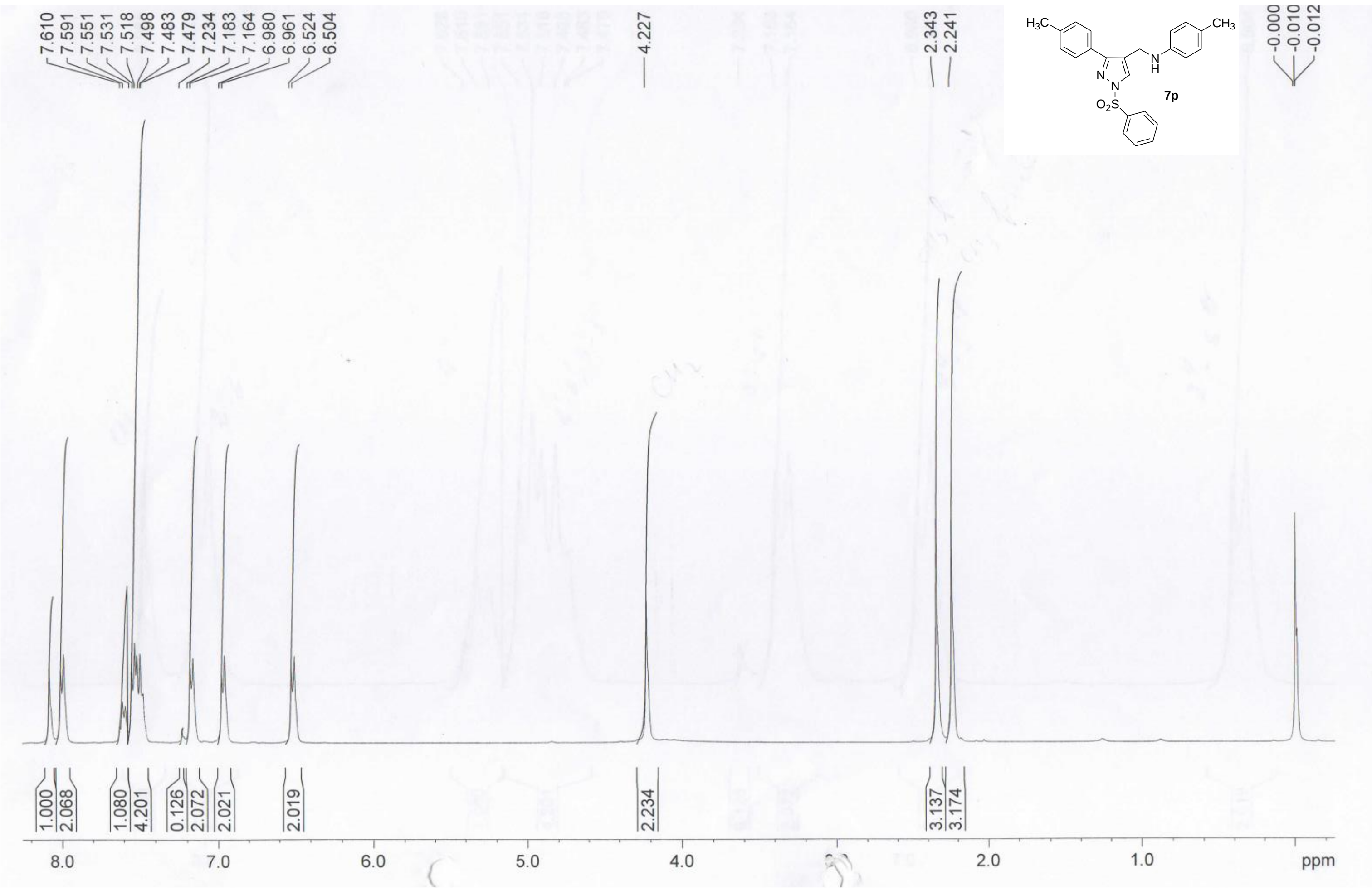

Fig. S 16. ^{1}H NMR spectrum of compound **7p**

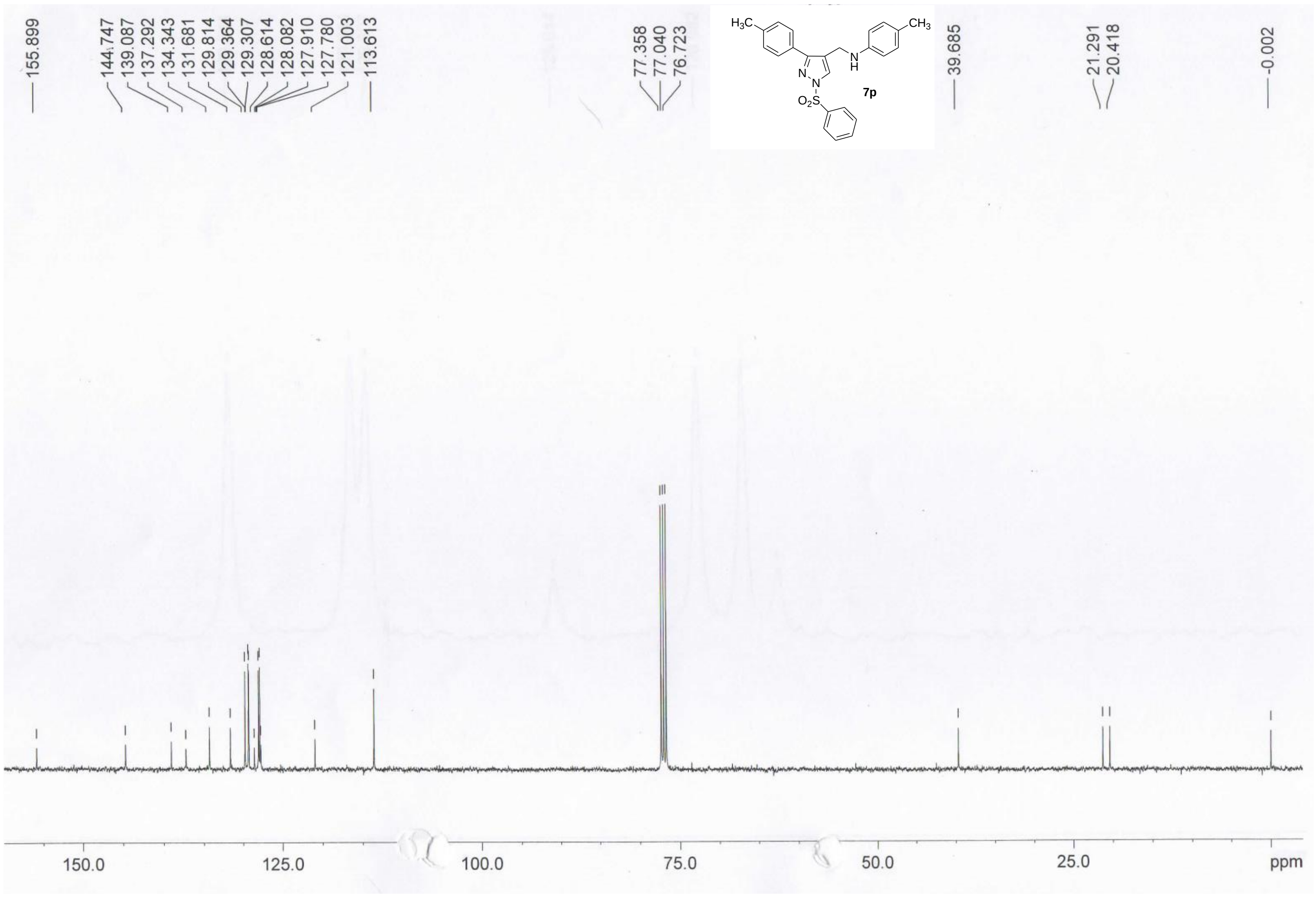

Fig. S 16. ^{13}C NMR spectrum of compound **7p**
