# Supplementary material for: Design, Synthesis, Antiviral Evaluation, and SAR Studies of New 1-(Phenylsulfonyl)-1H-Pyrazol−4-yl-Methylaniline Derivatives
Source: Front Chem. 2019 Apr 9;7:214. doi: 10.3389/fchem.2019.00214 (PMC6465675; doi:10.3389/fchem.2019.00214)
Supplement: Supplementary file 2 [file Data_Sheet_2.PDF]

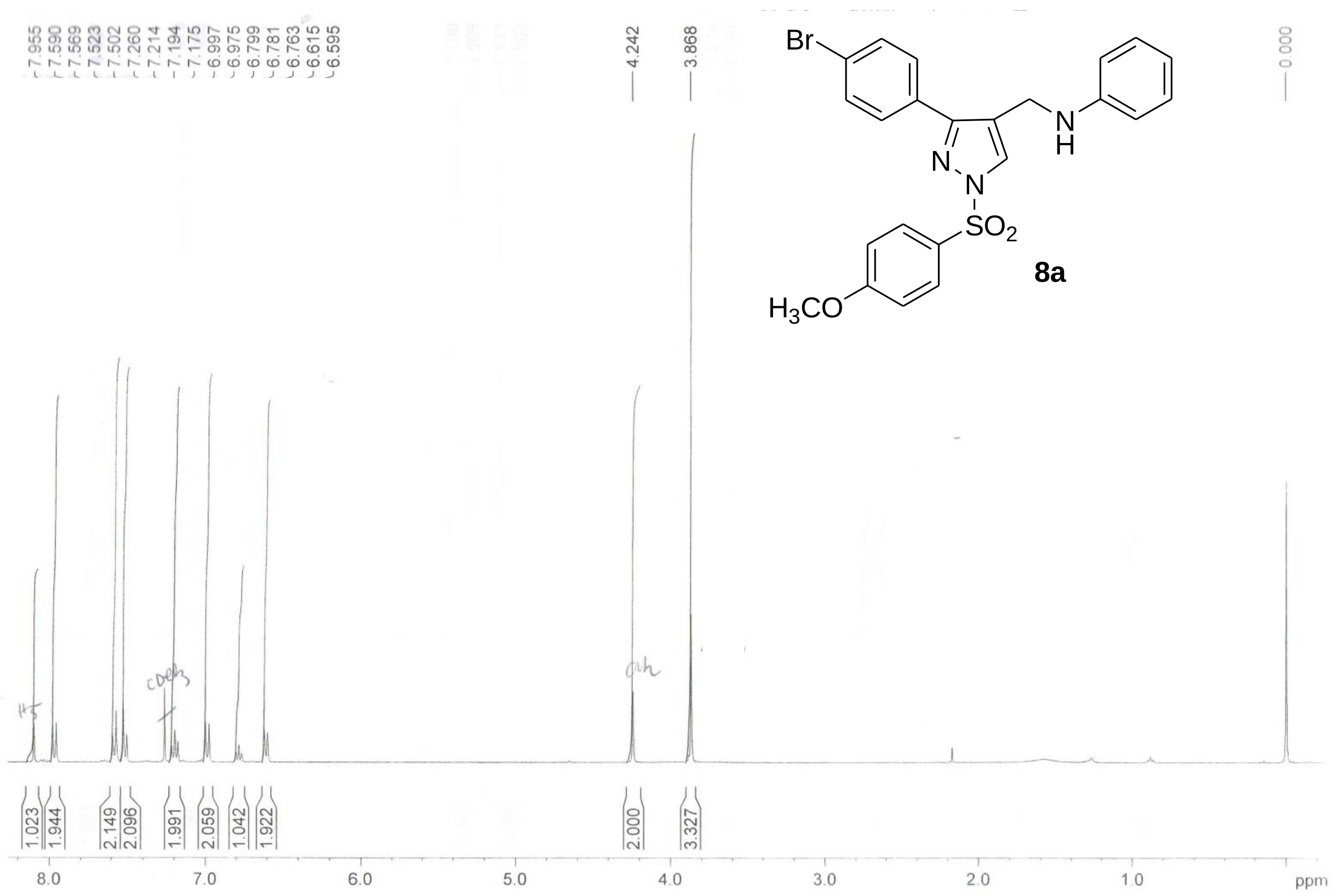

Fig. S 17 ^{1}H NMR spectrum of compound **8a**

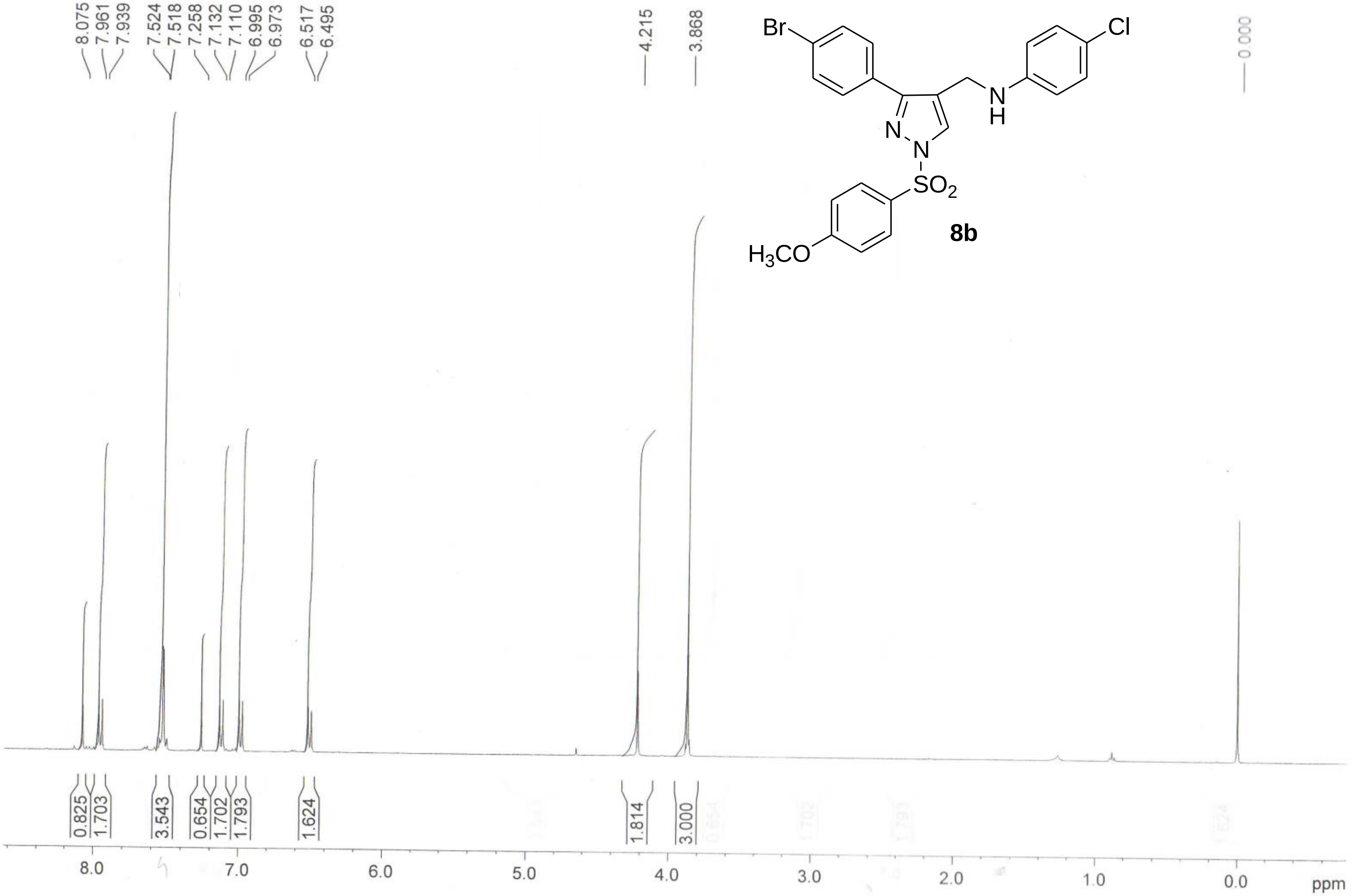

Fig. S18. ^{1}H NMR spectrum of compound **8b**

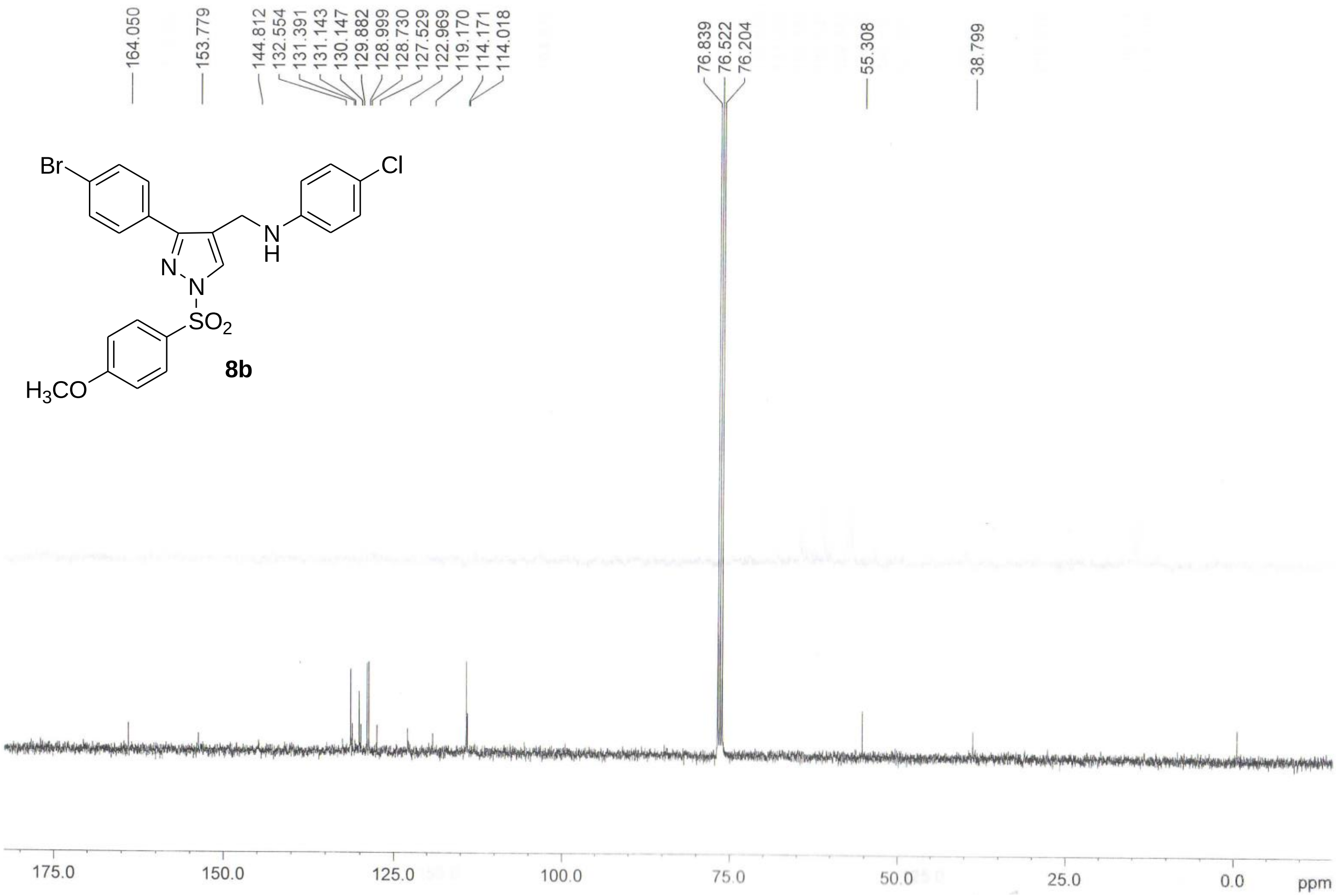

Fig. S18. ^{13}C NMR spectrum of compound **8b**

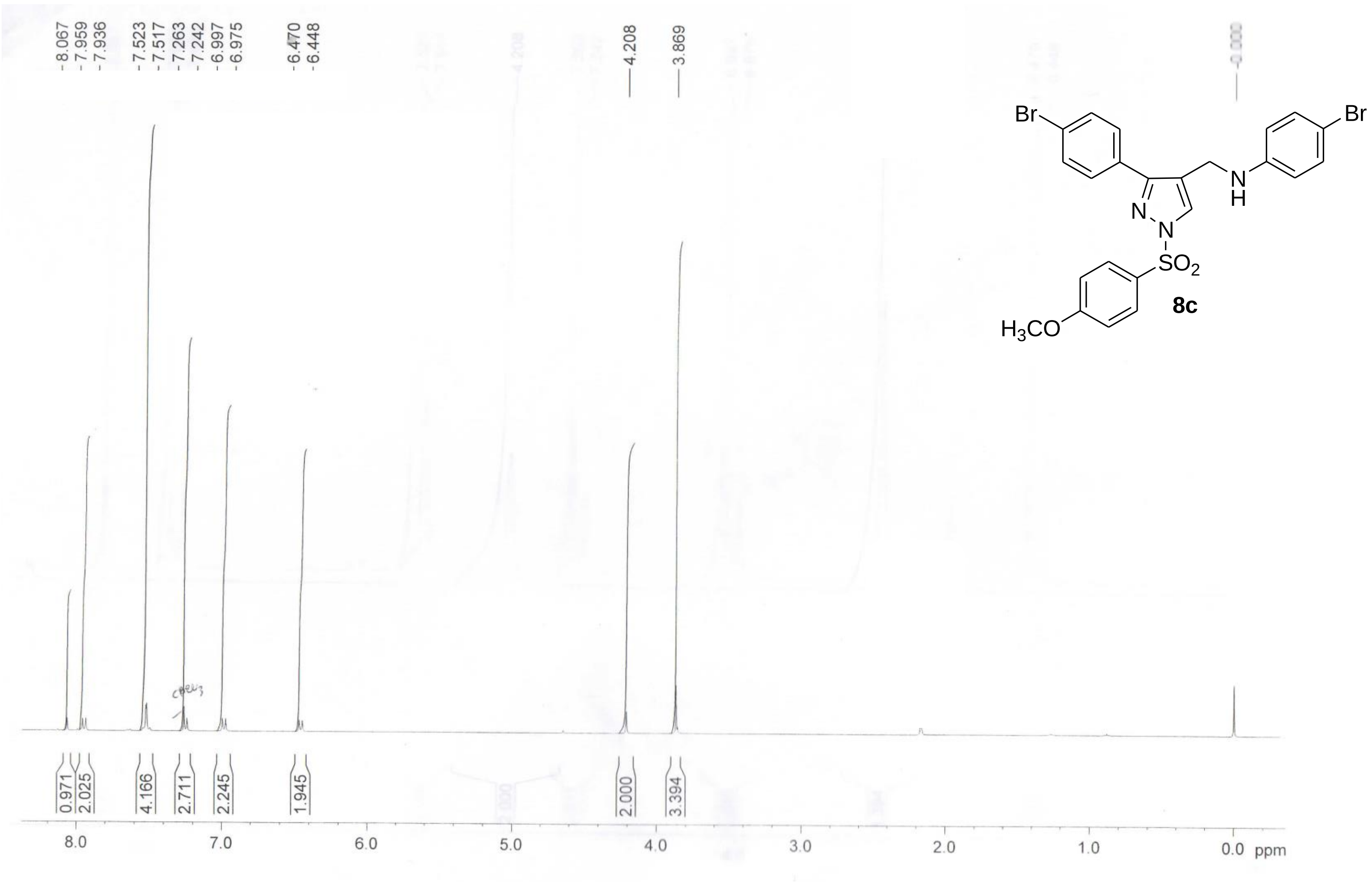

Fig. S 19. ^{1}H NMR spectrum of compound **8c**

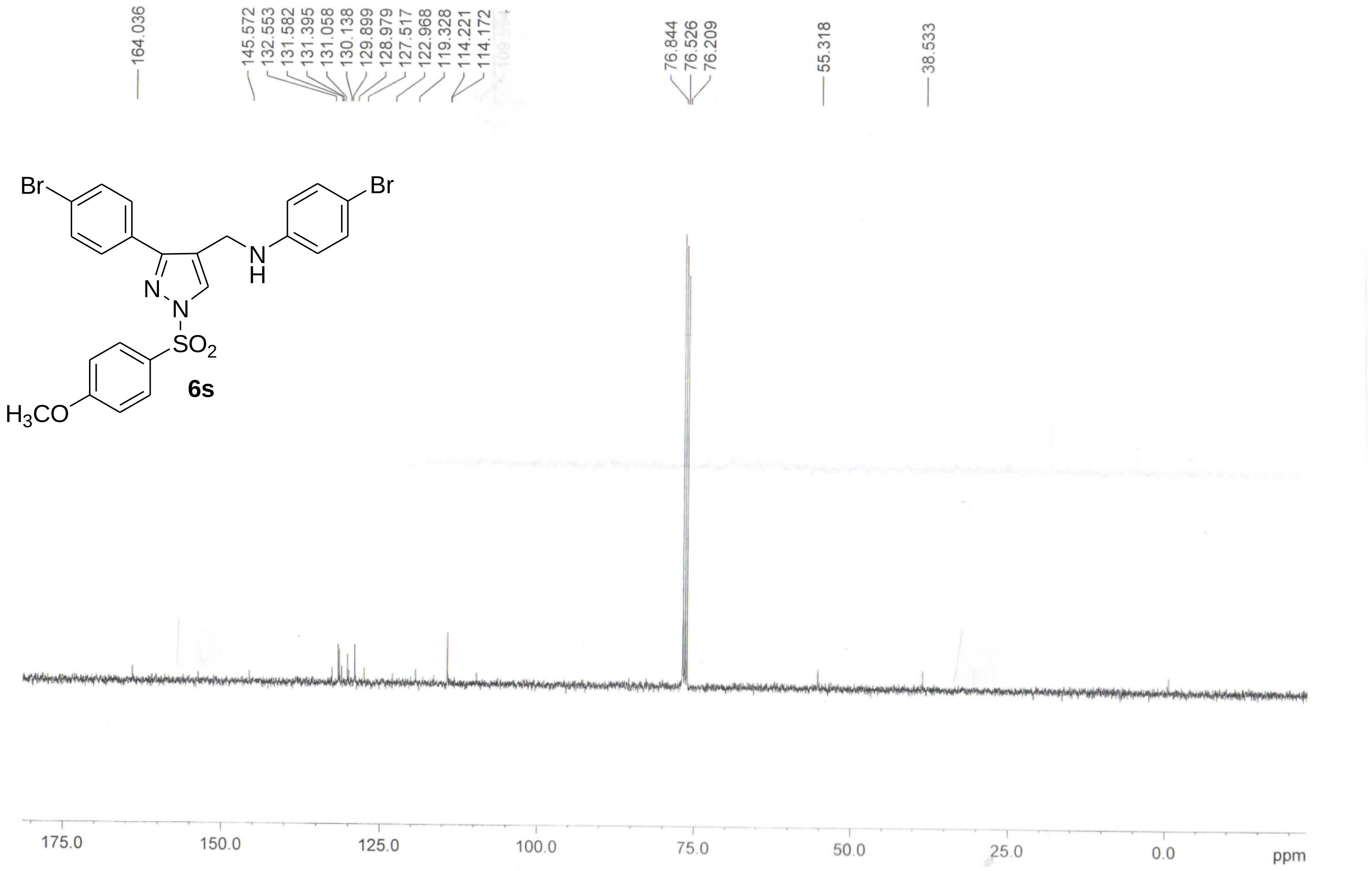

Fig. S 19. ^{13}C NMR spectrum of compound **8c**

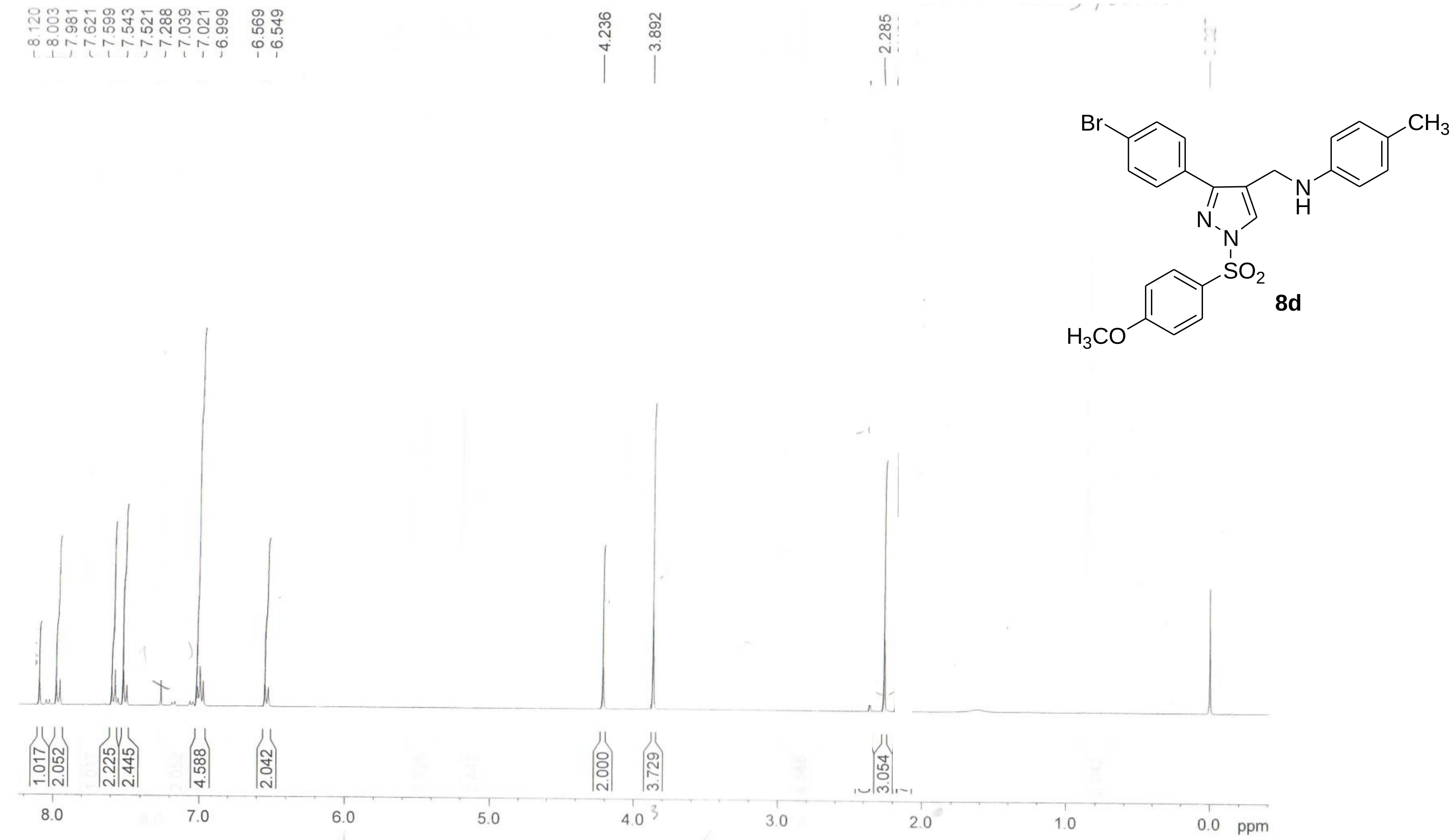

Fig. S 20 ^{1}H NMR spectrum of compound **8d**

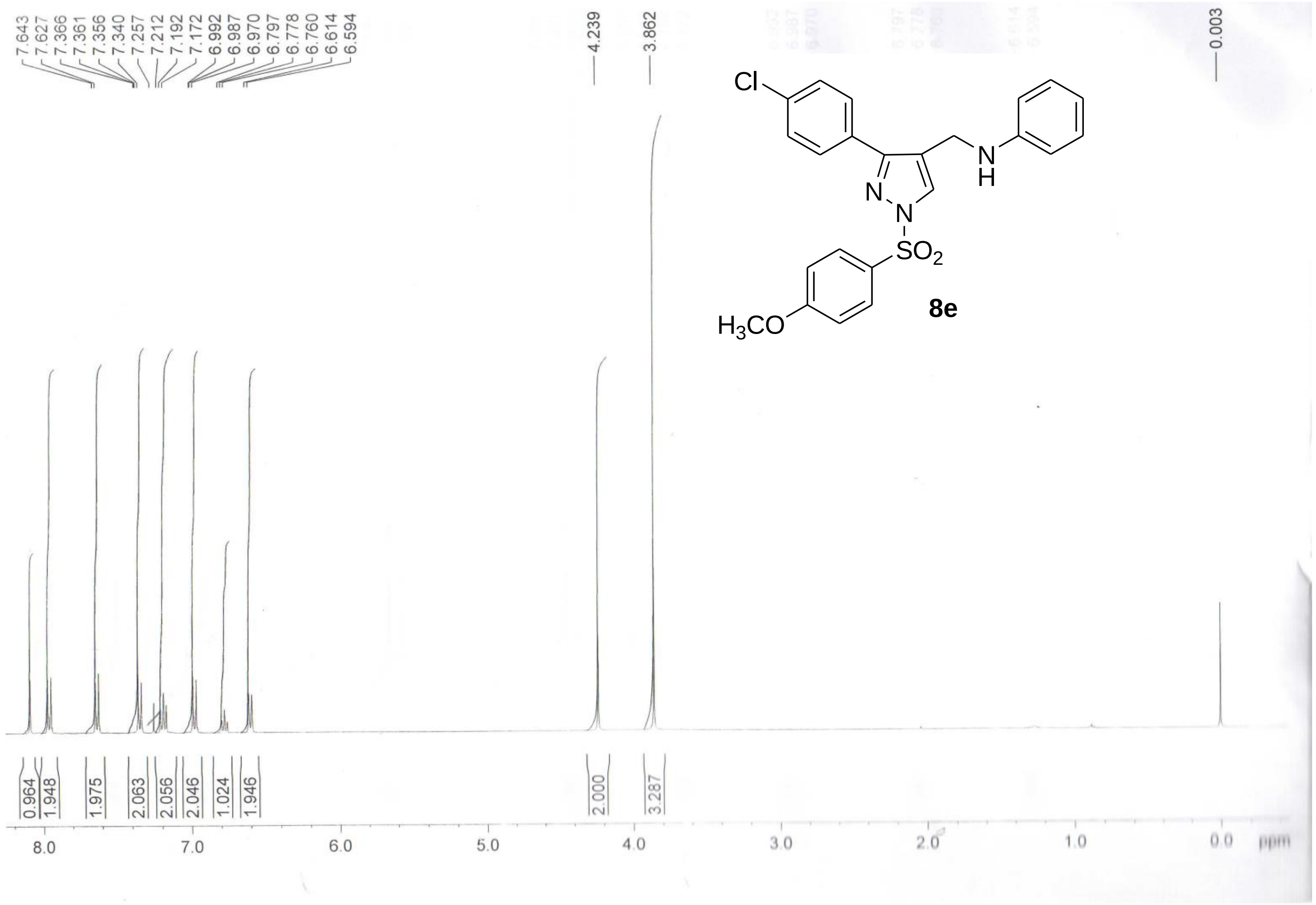

Fig. S21. ^{1}H NMR spectrum of compound **8e**

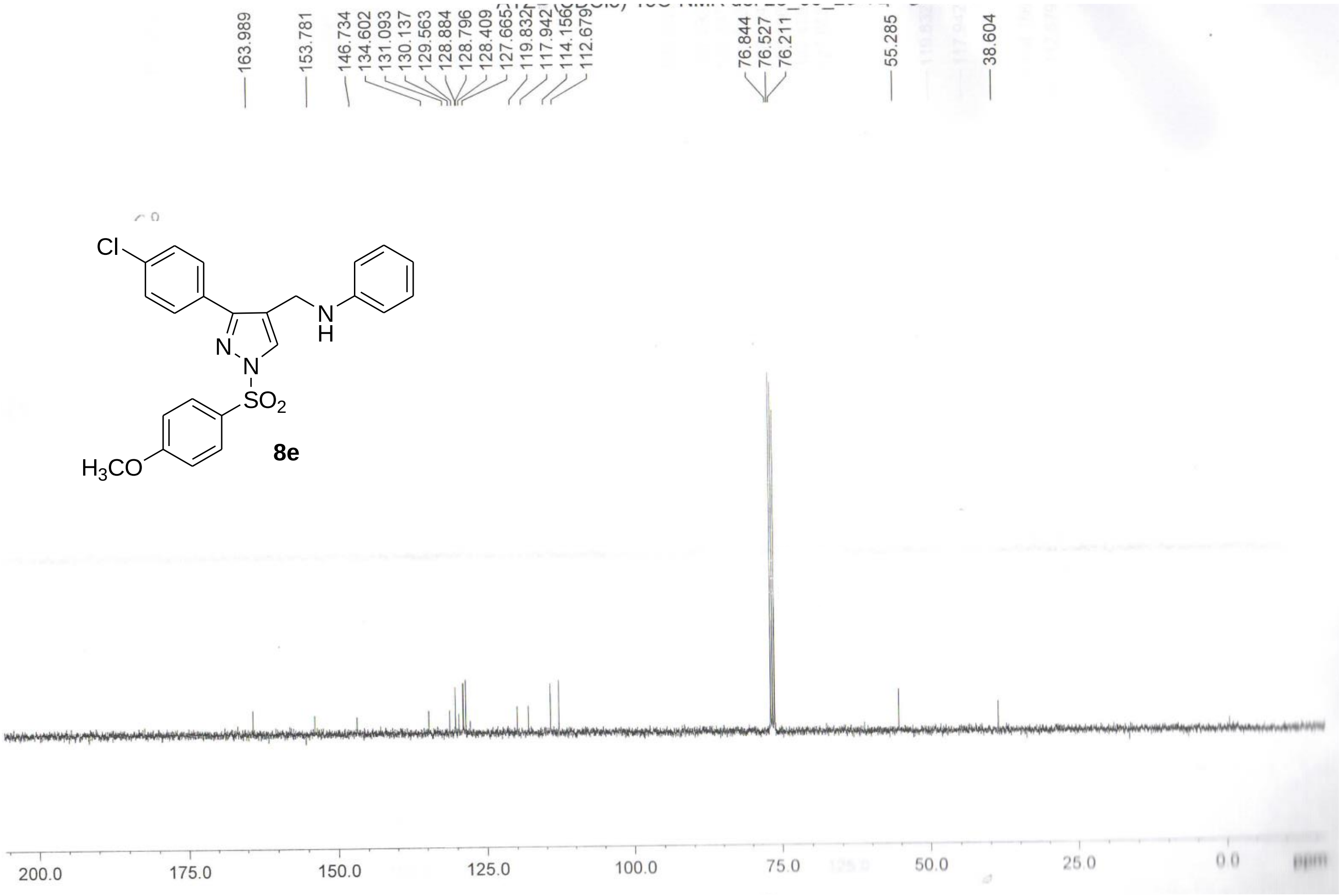

Fig. S21. ^{13}C NMR spectrum of compound **8e**

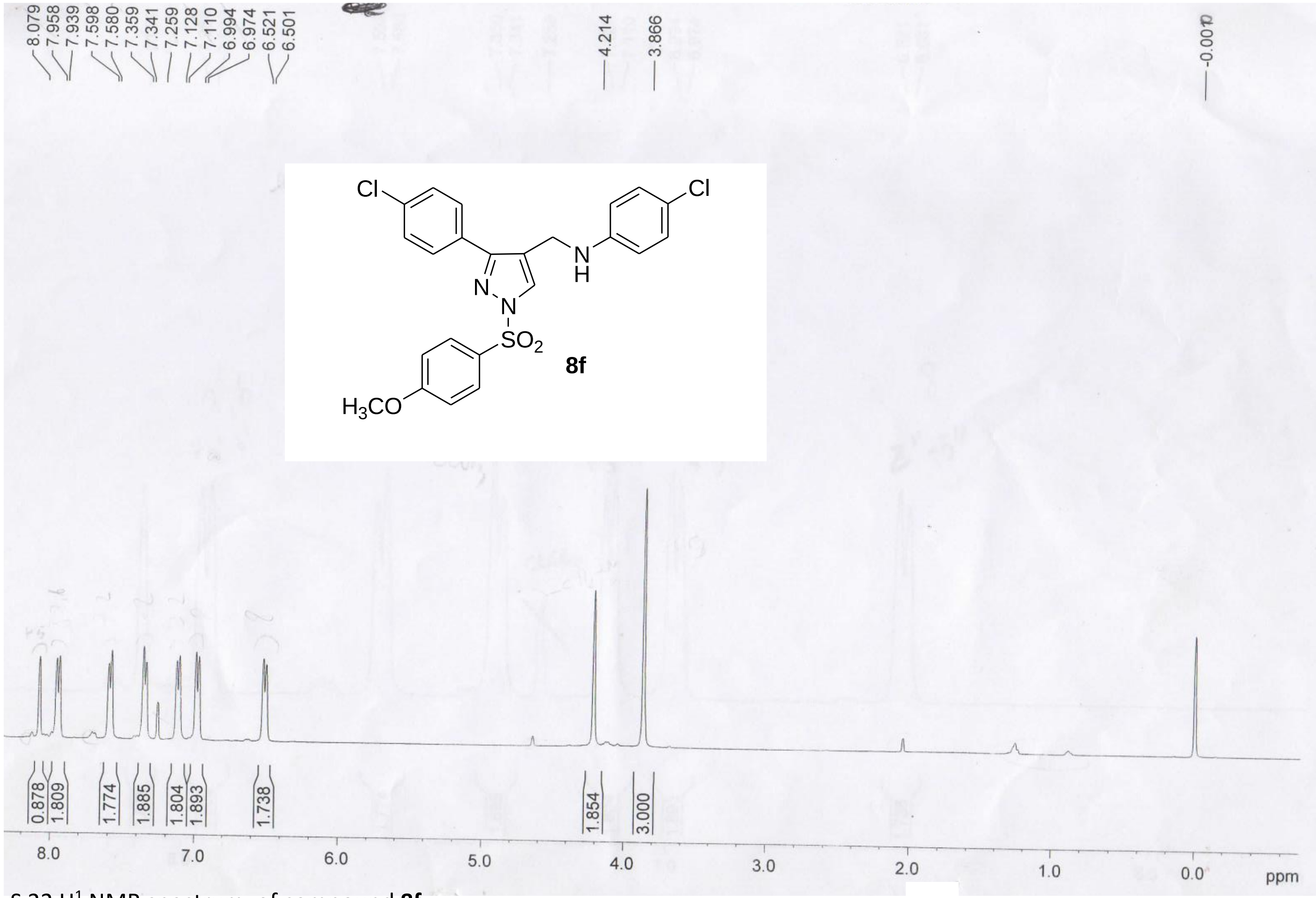

Fig. S 22 H[1] NMR spectrum of compound **8f**

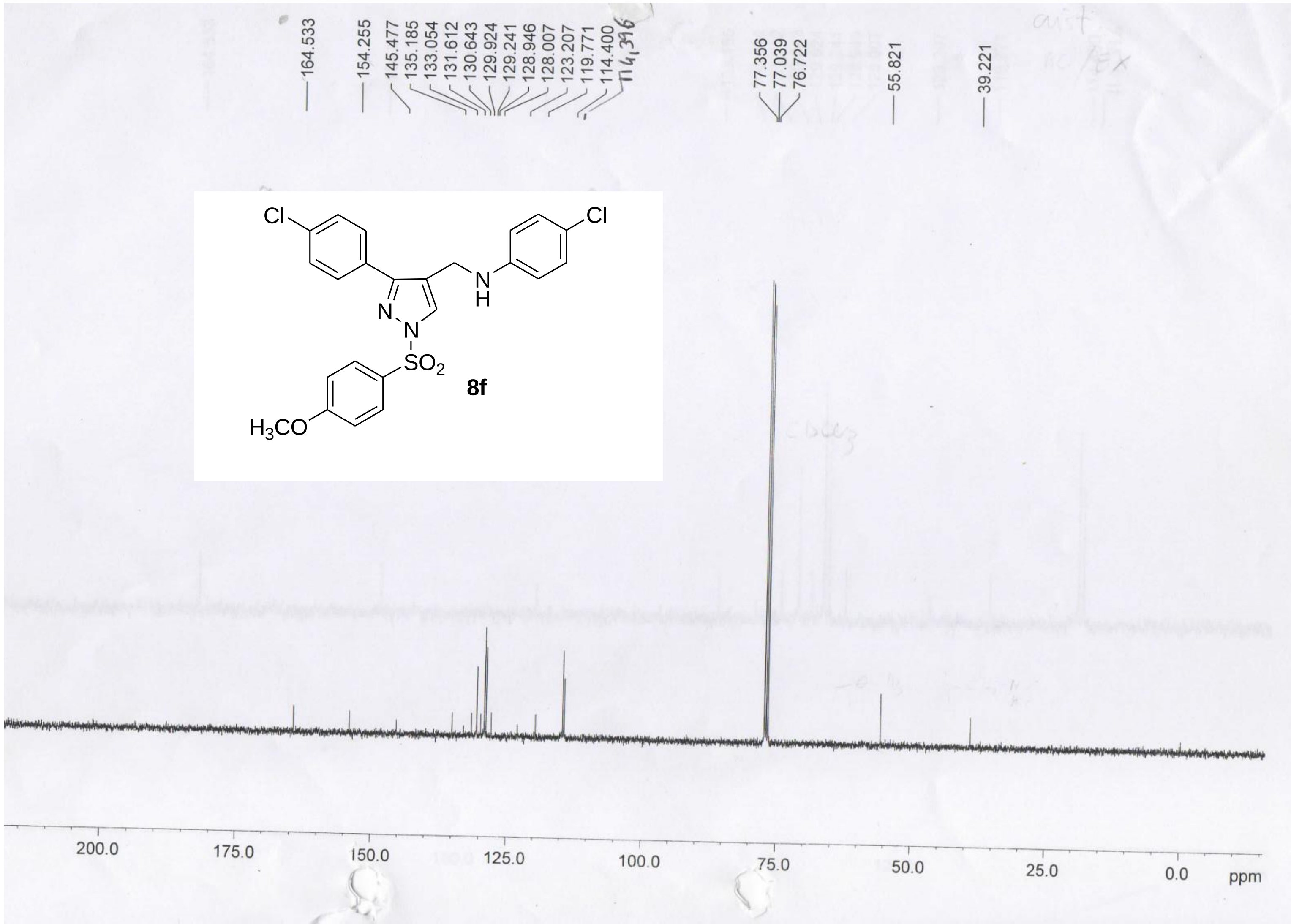

Fig. S 22 . ^{13}C NMR spectrum of compound **8f**

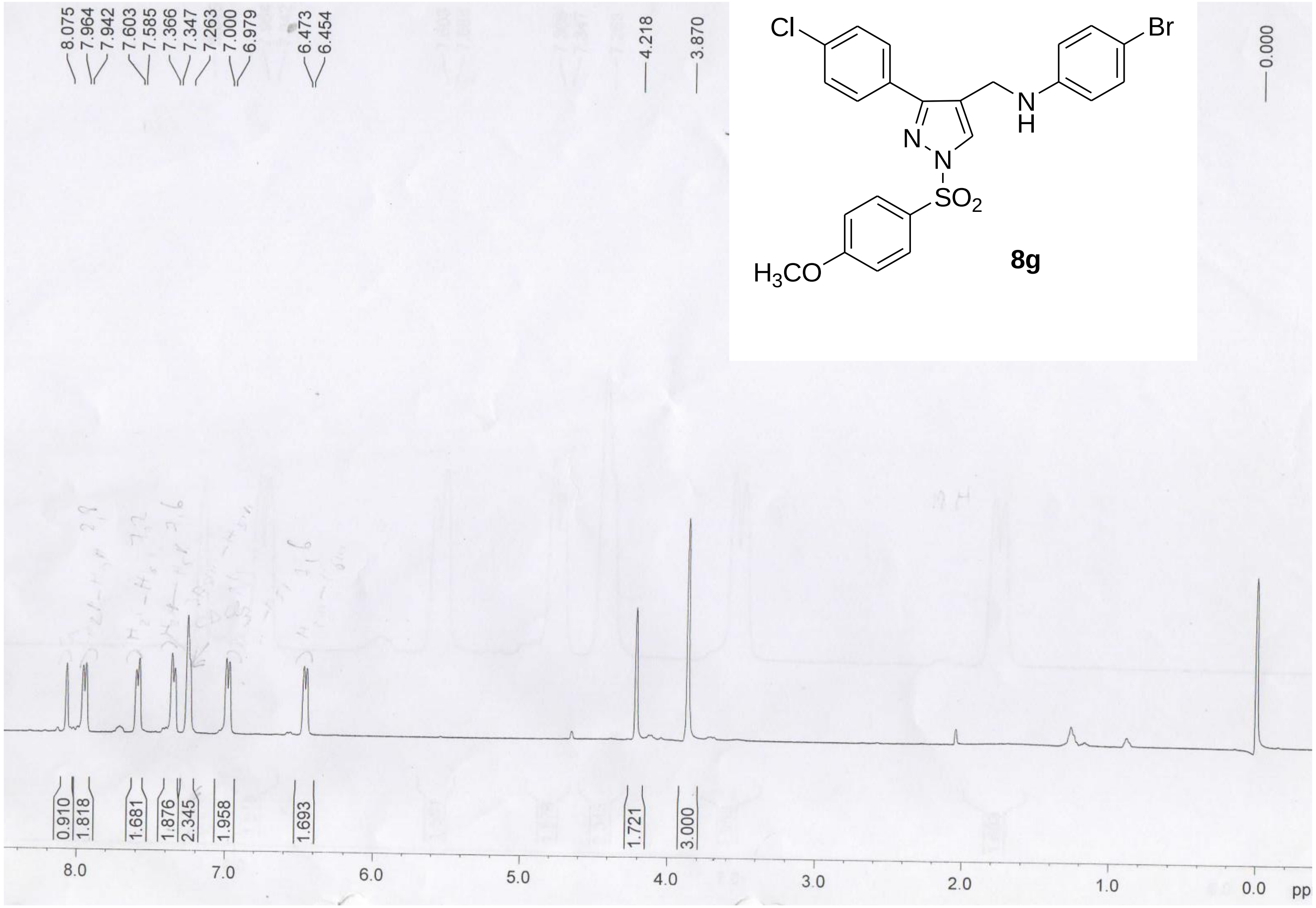

Fig. S 23. ^{1}H NMR spectrum of compound **8g**

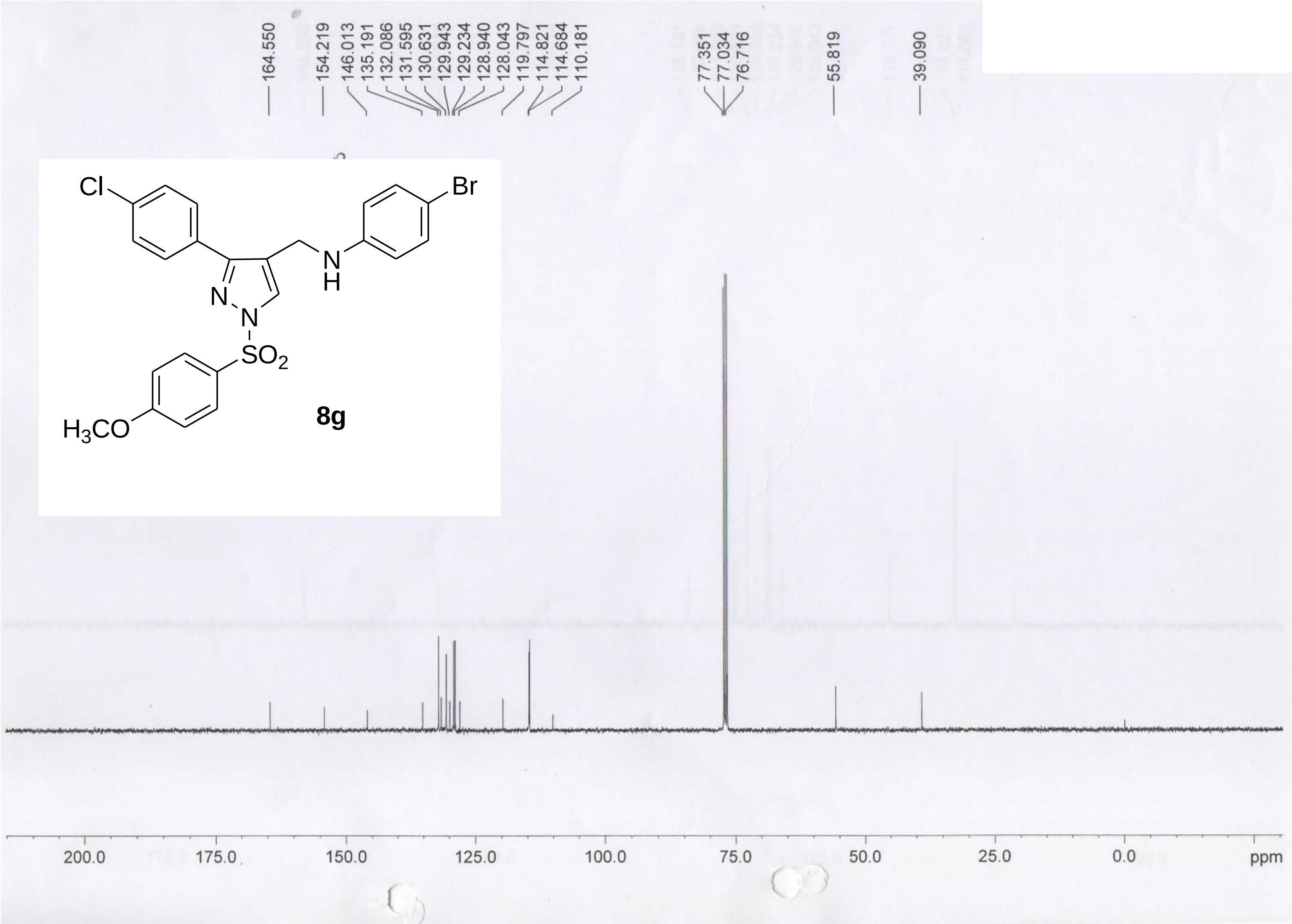

Fig. S 23. ^{13}C NMR spectrum of compound **8g**

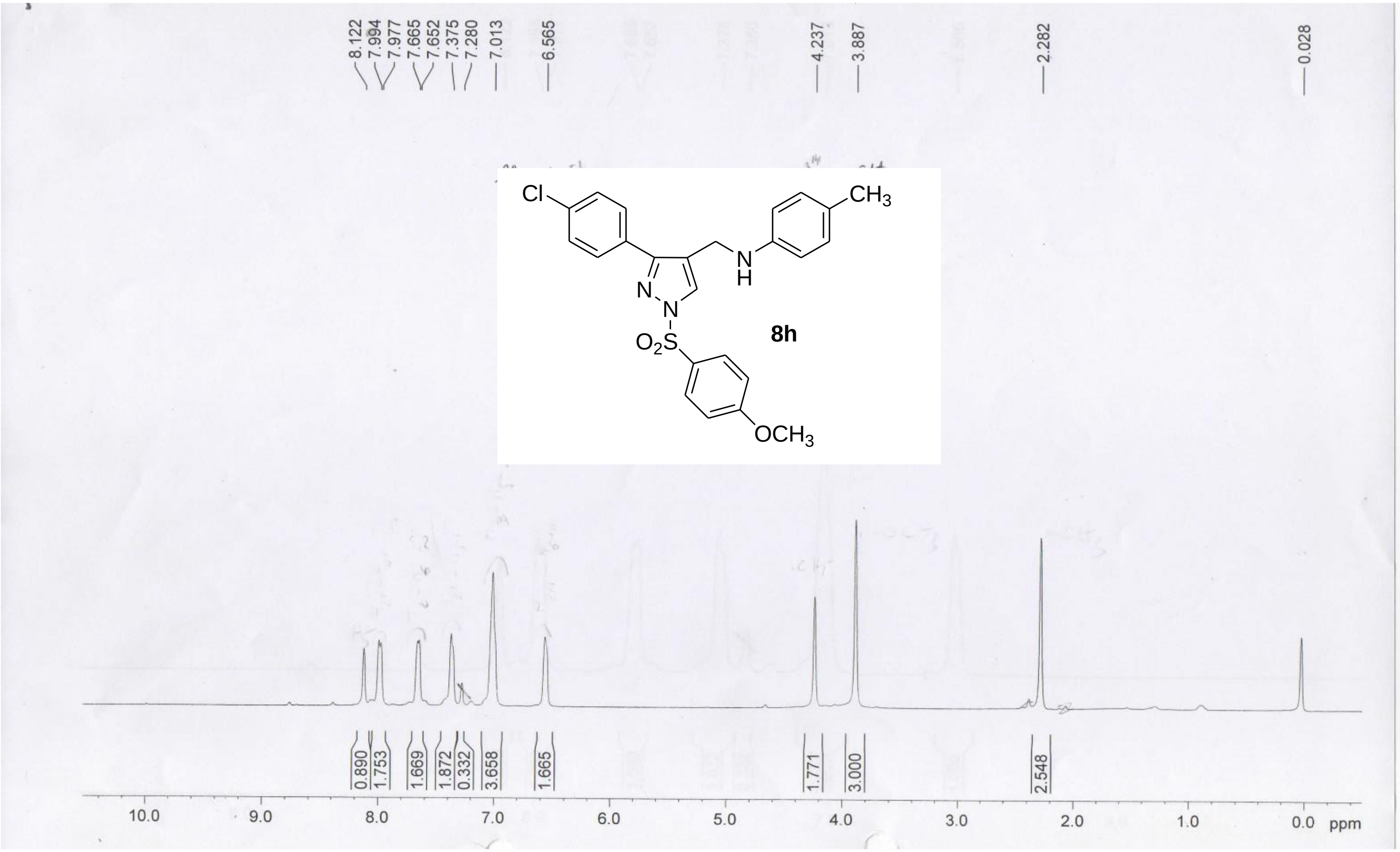

Fig. S 24 ^{1}H NMR spectrum of compound **8h**

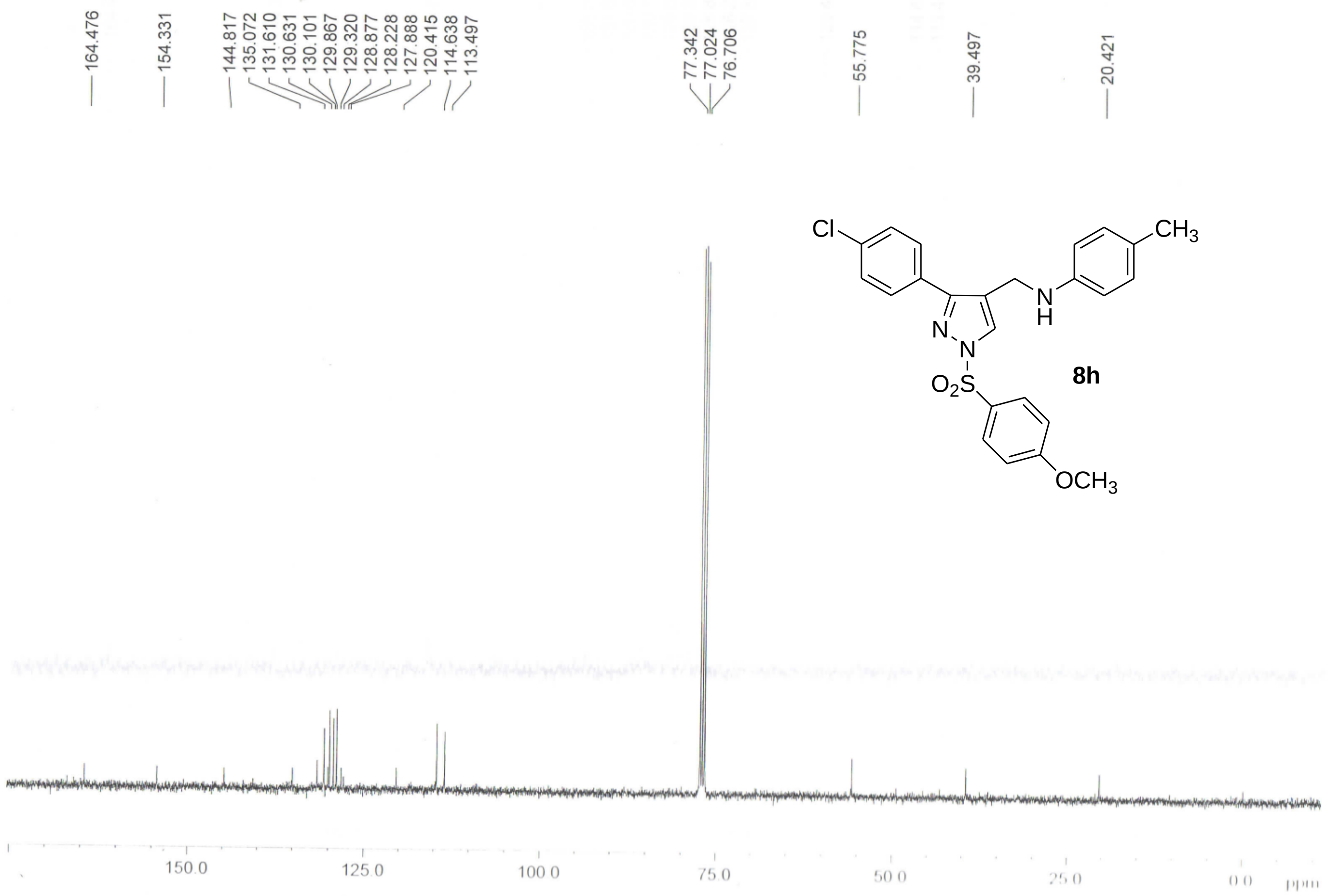

Fig. S 24 ^{13}C NMR spectrum of compound **8h**

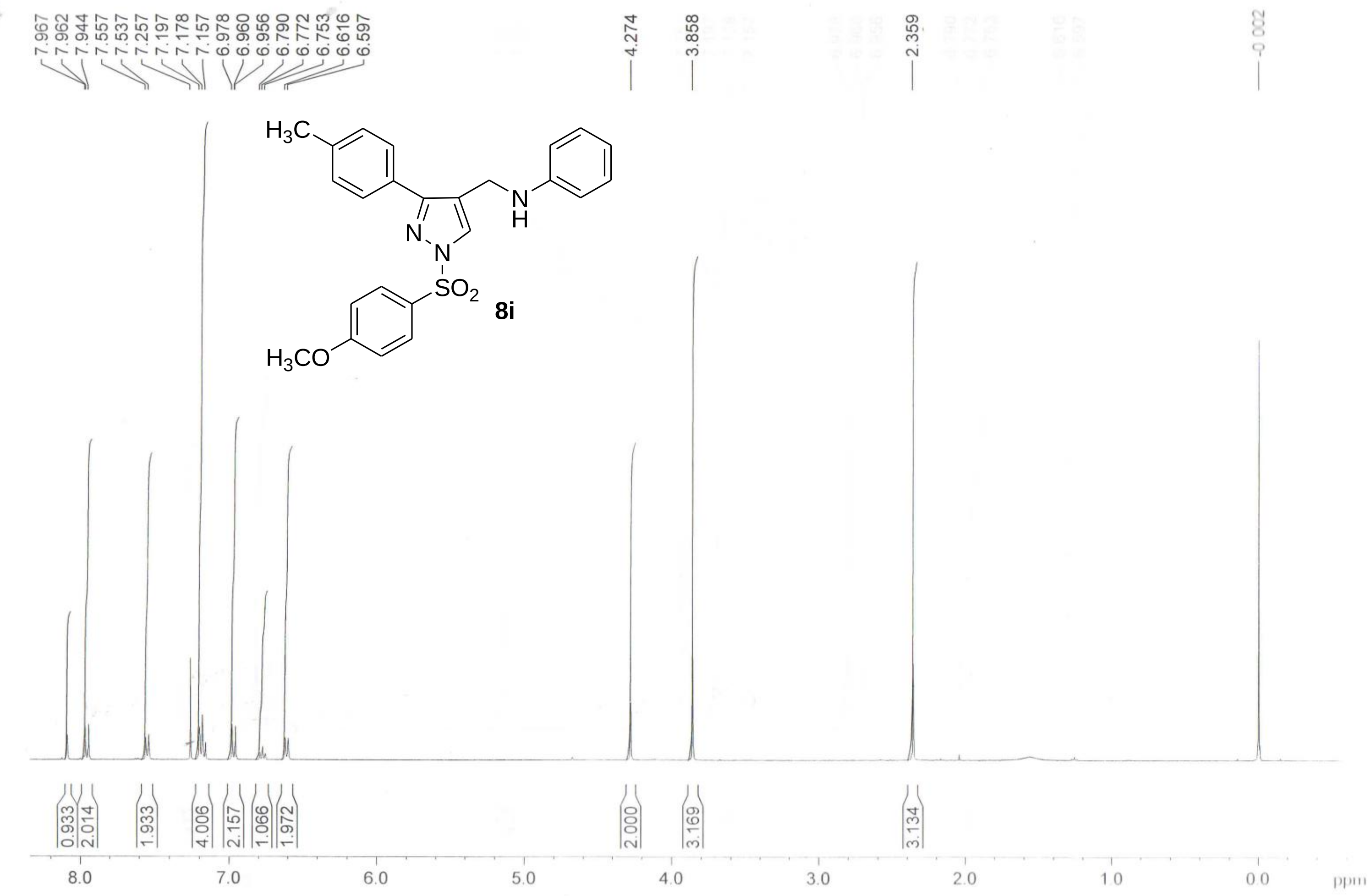

Fig. S 25 ^{1}H NMR spectrum of compound **8i**

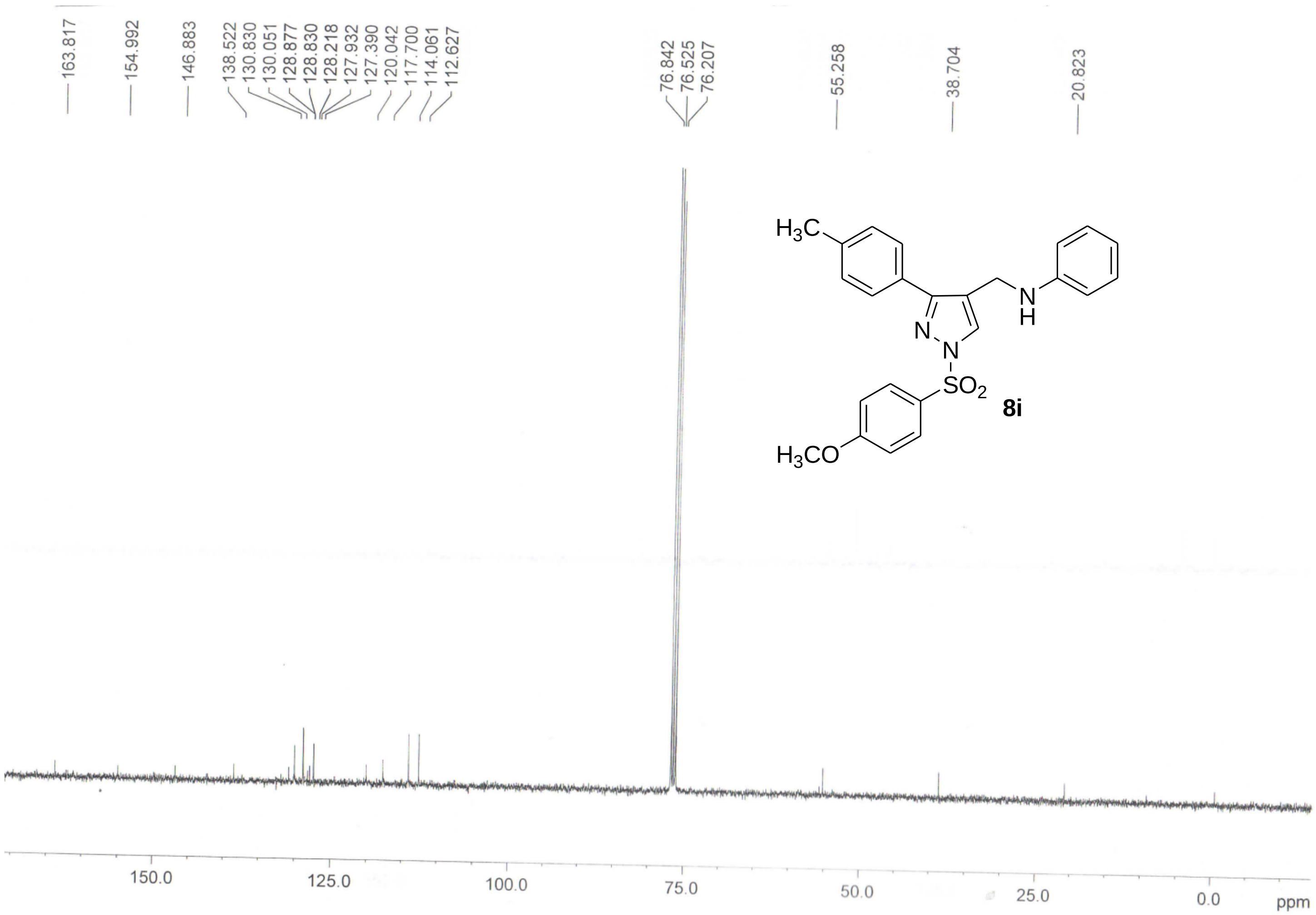

Fig. S 25 ^{13}C NMR spectrum of compound **8i**

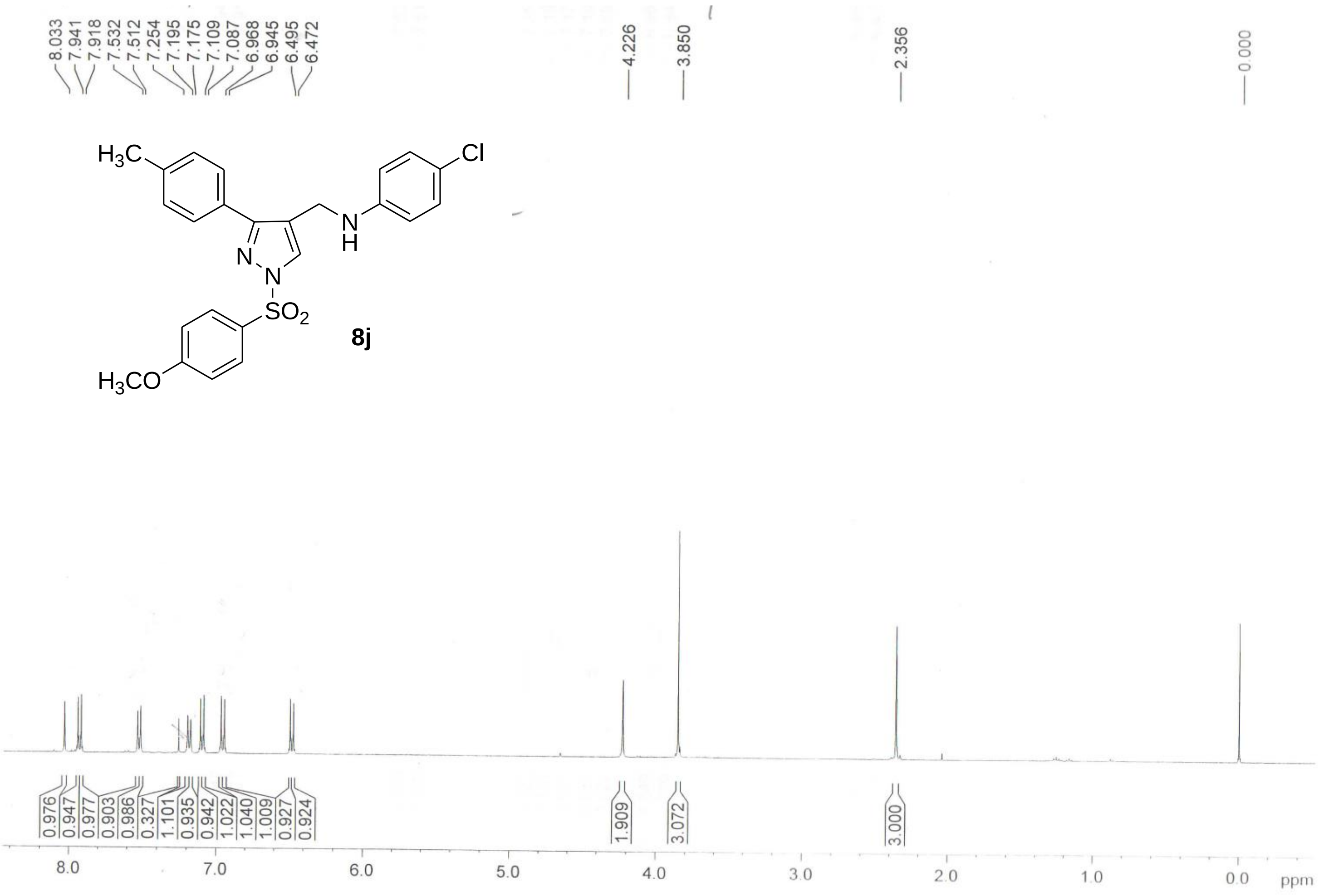

Fig. S26 ^{1}H NMR Spectrum of compound **8j**

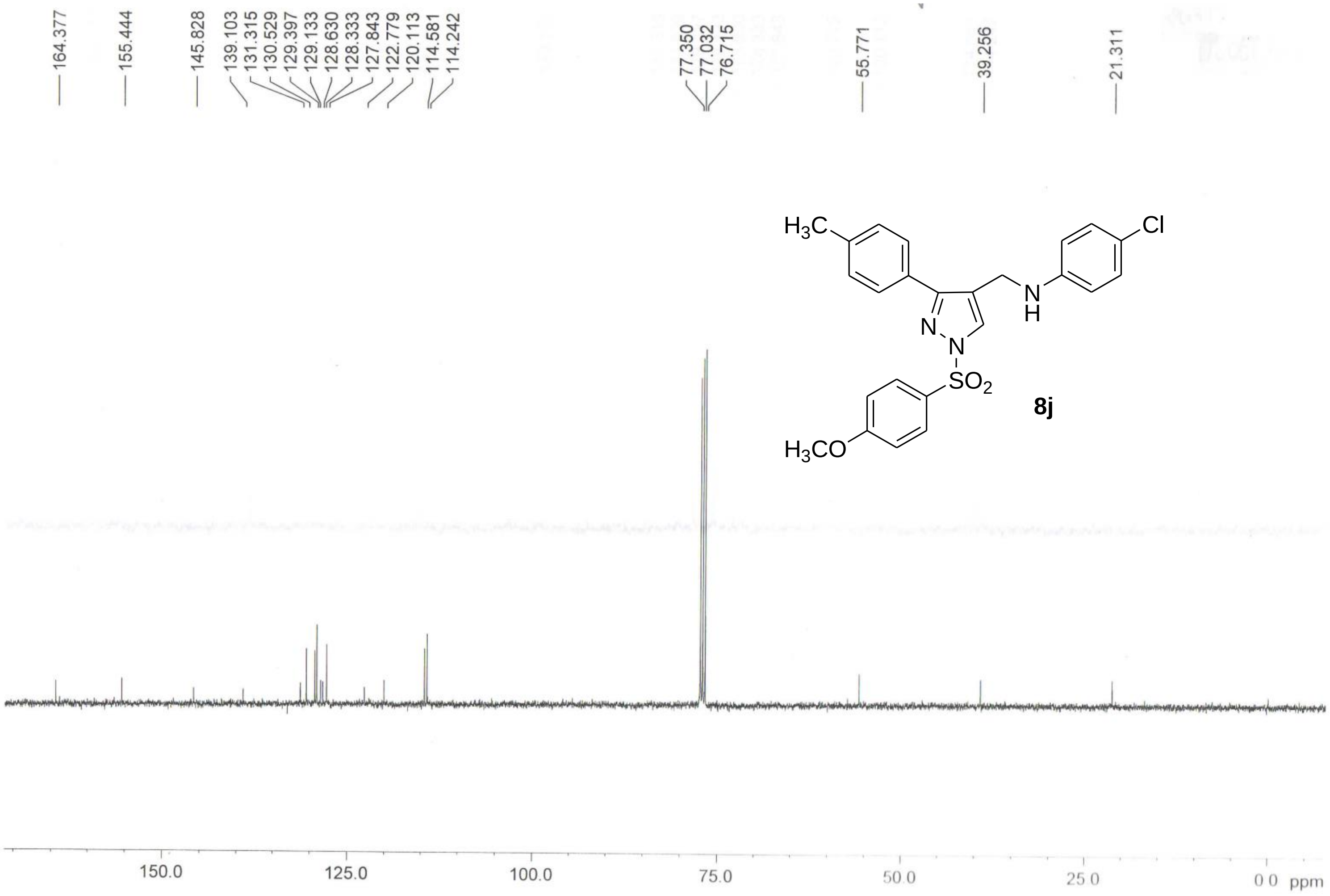

Fig. S26 ^{13}C NMR Spectrum of compound **8j**

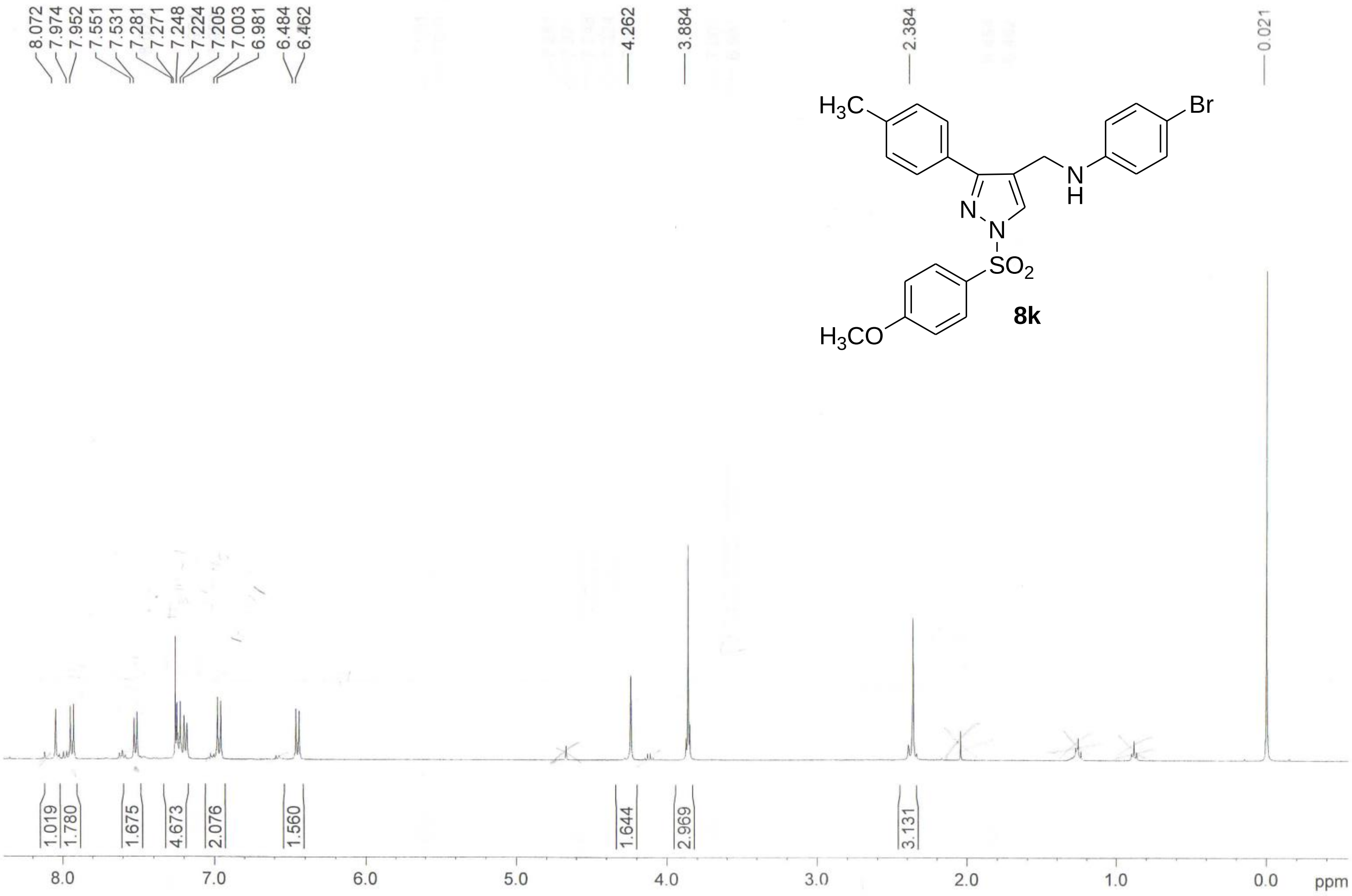

Fig. S 27 ^{1}H NMR spectrum of compound **8k**

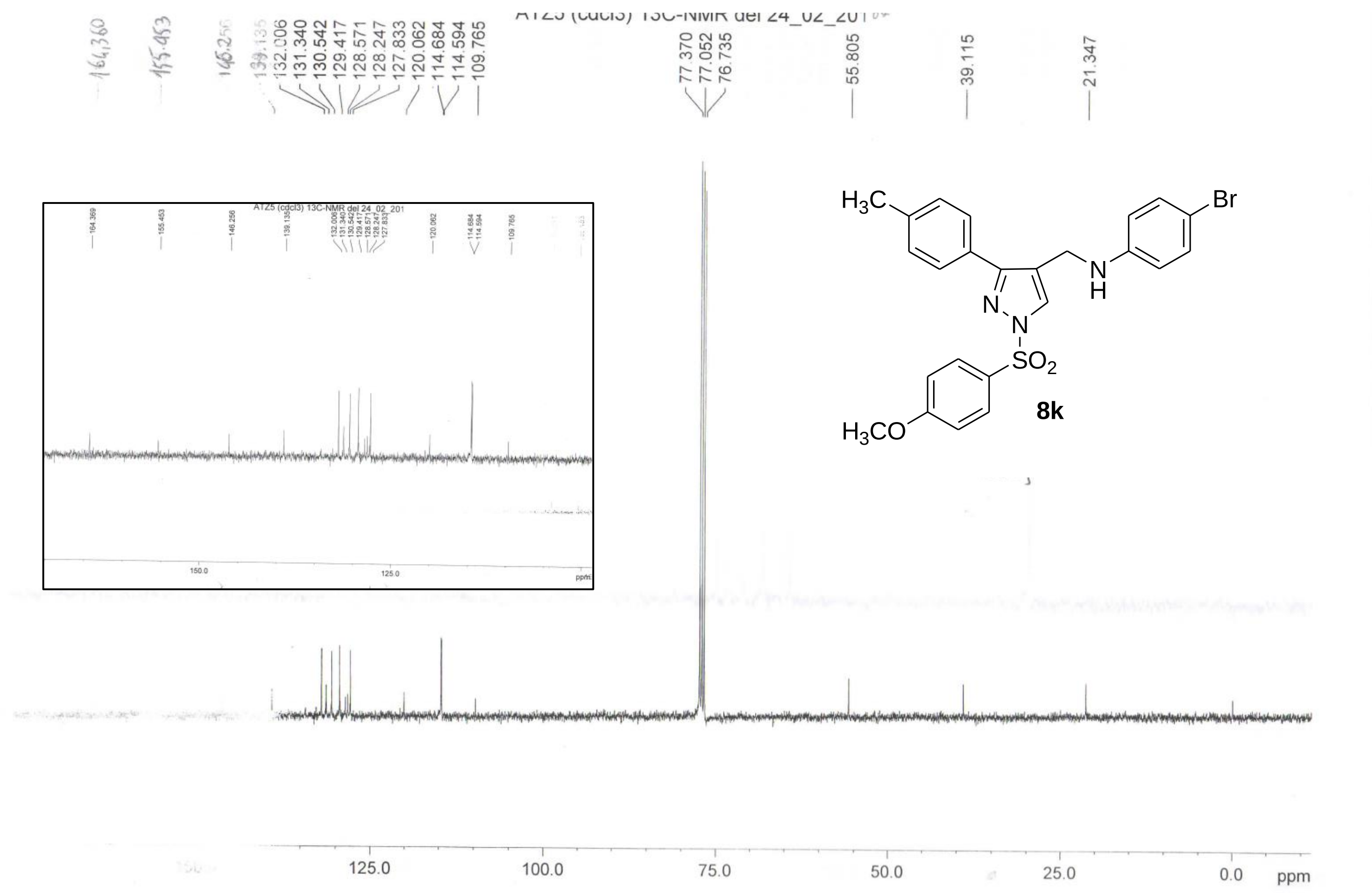

Fig. S 27 ^{13}C NMR spectrum of compound **8k**

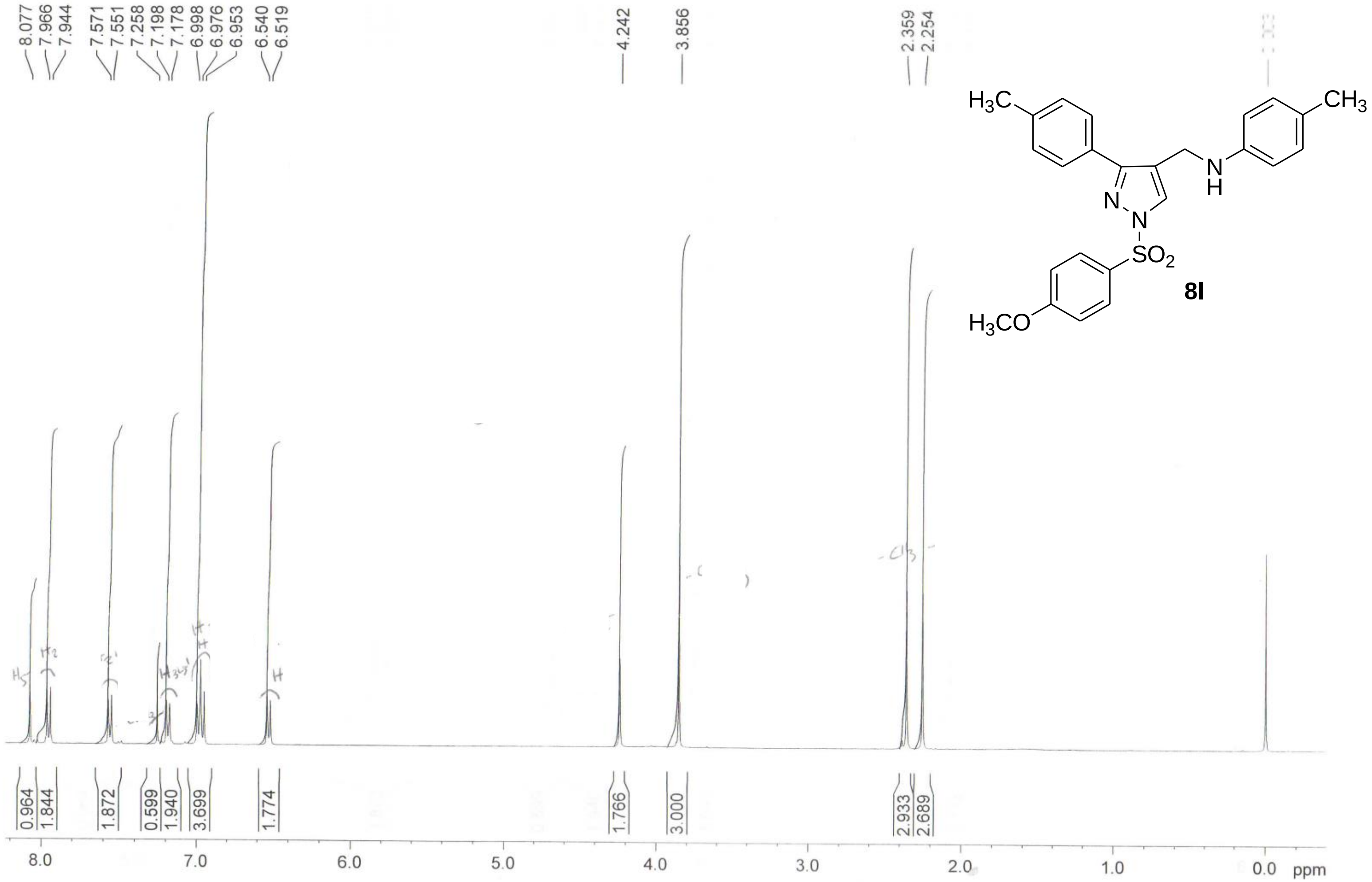

Fig. S28. ¹H NMR spectrum of compound **8l**

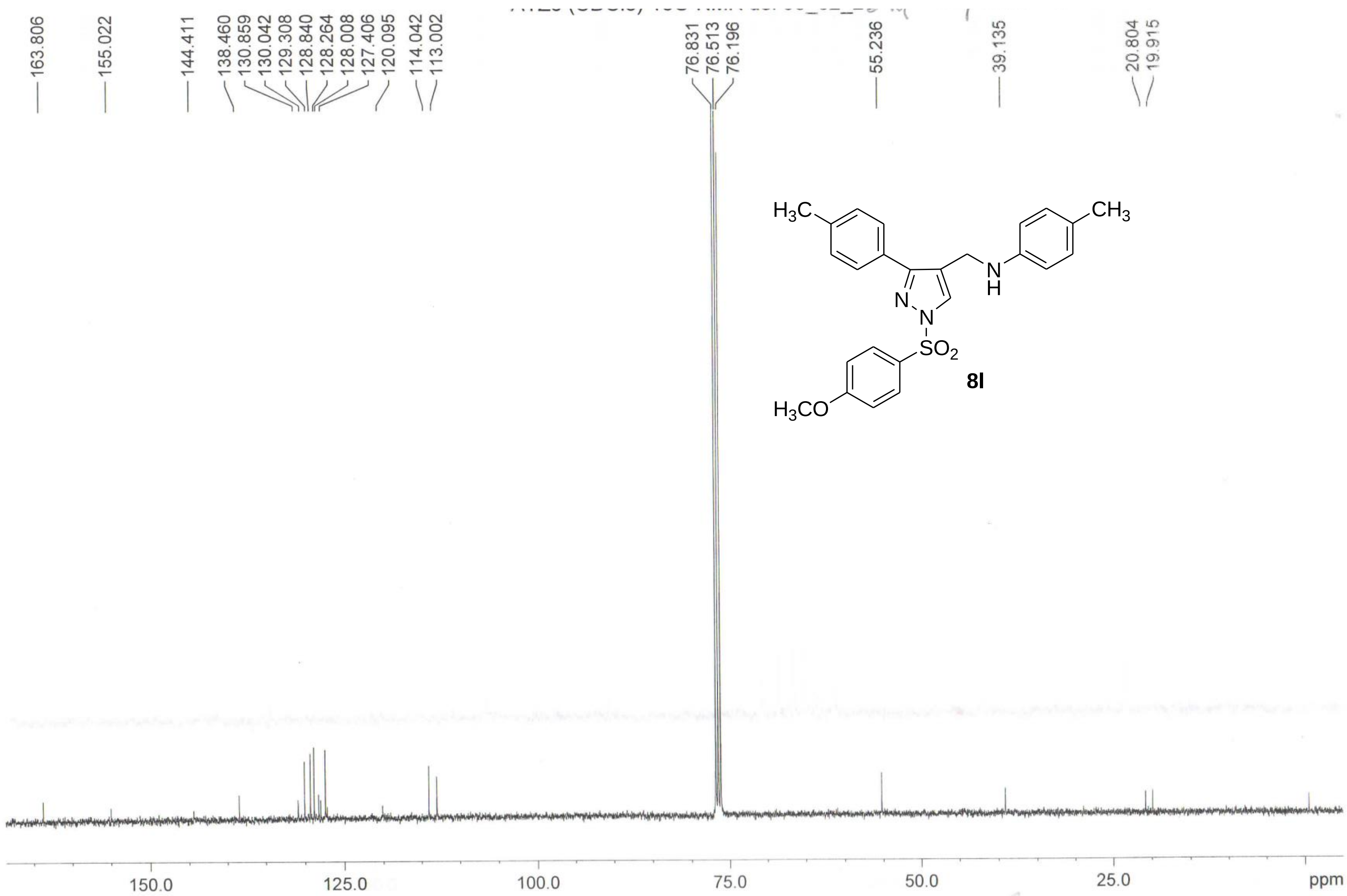

Fig. S28. ^{13}C NMR spectrum of compound **8l**
