# Supplementary material for: Design, Synthesis, Antiviral Evaluation, and SAR Studies of New 1-(Phenylsulfonyl)-1H-Pyrazol−4-yl-Methylaniline Derivatives
Source: Front Chem. 2019 Apr 9;7:214. doi: 10.3389/fchem.2019.00214 (PMC6465675; doi:10.3389/fchem.2019.00214)
Supplement: Supplementary file 3 [file Data_Sheet_3.PDF]

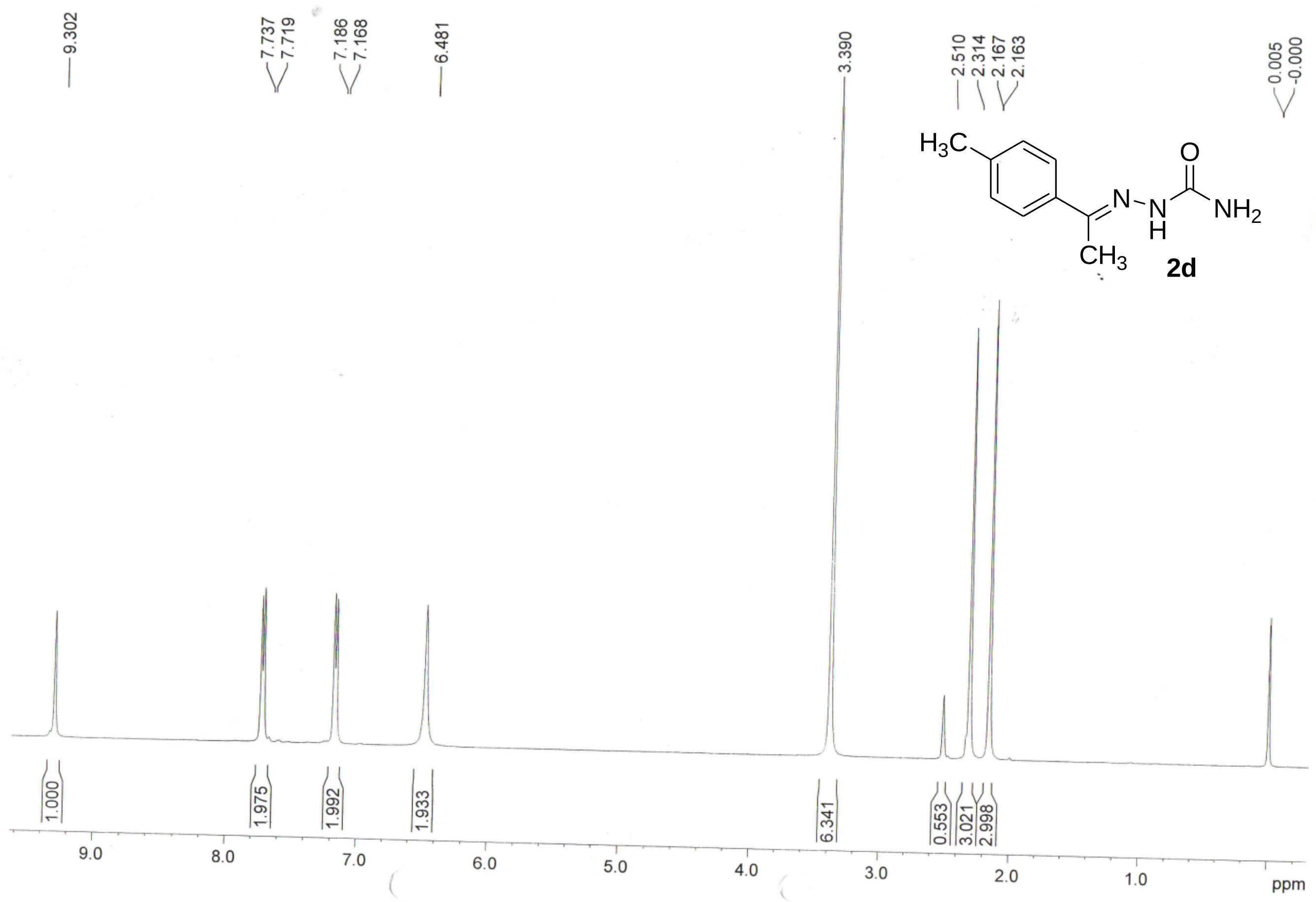

Fig. S29. ^{1}H NMR spectrum of compound **2d**

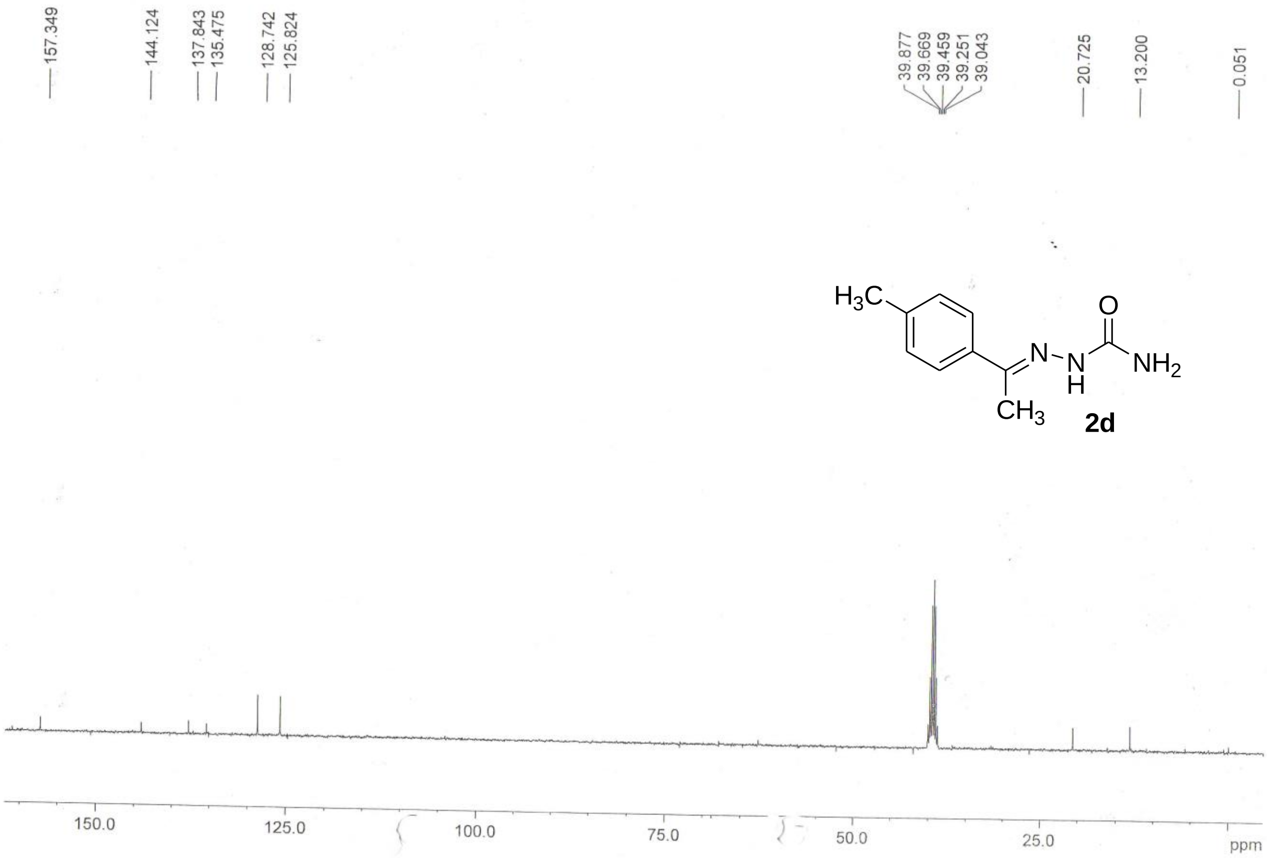

Fig. S29. ^{13}C NMR spectrum of compound **2d**

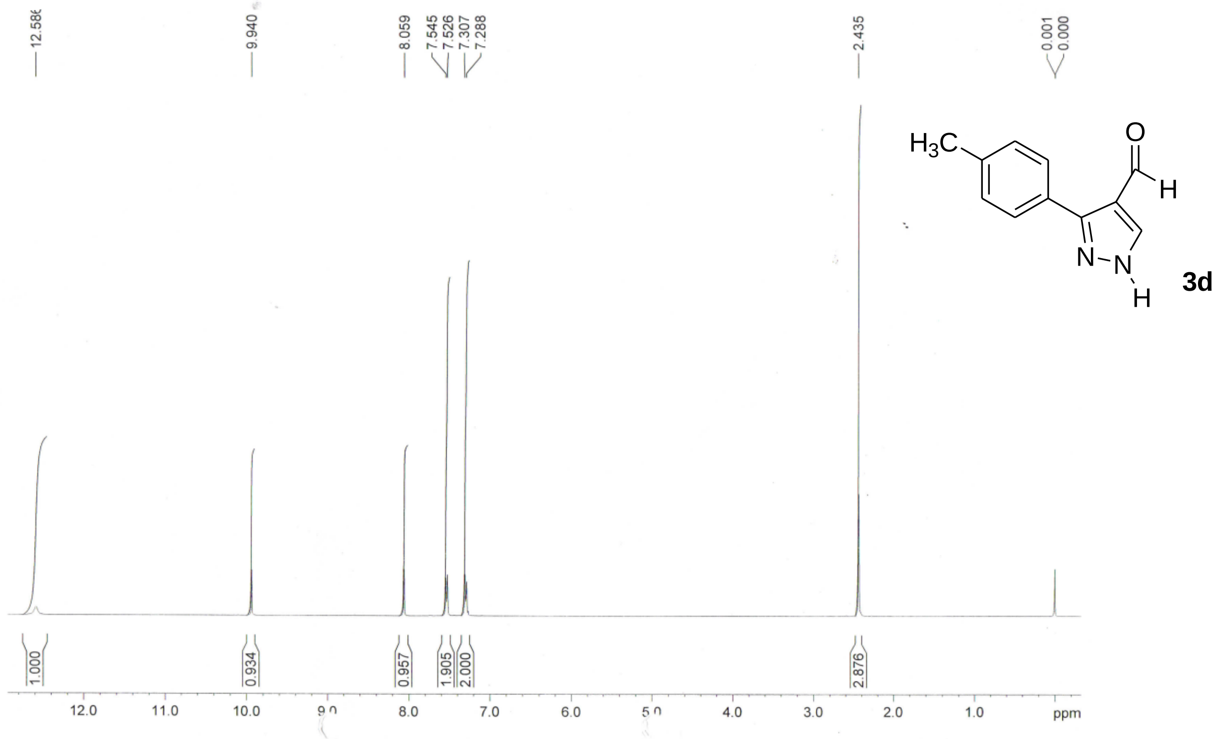

Fig. S30. ^{1}H NMR spectrum of compound **3d**

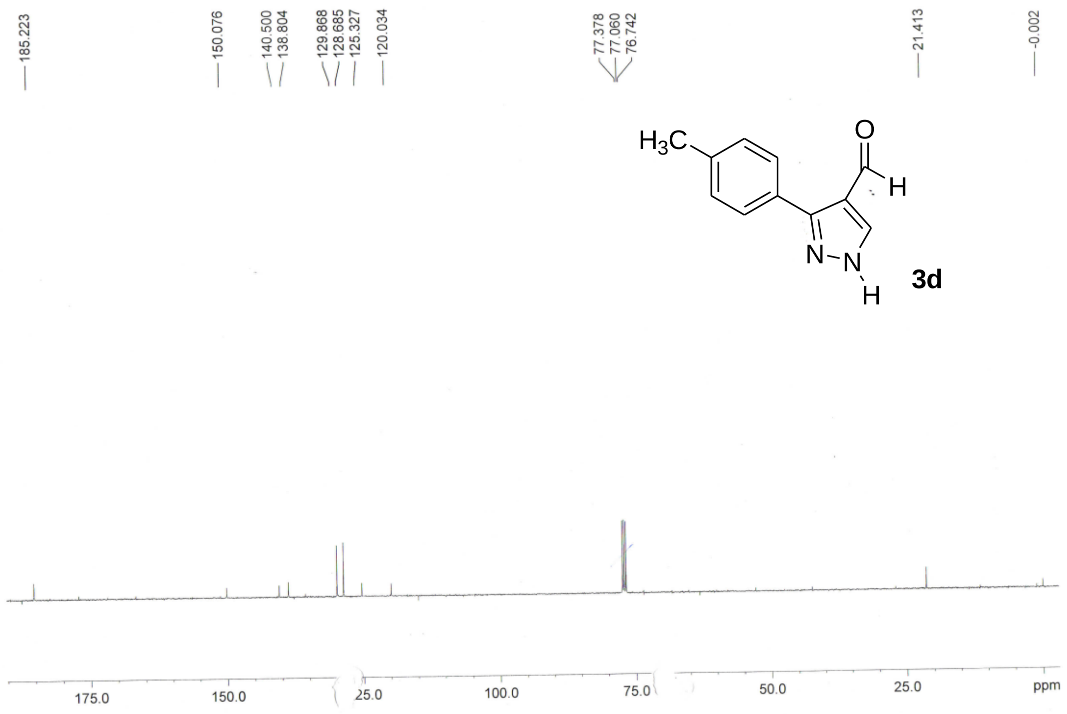

Fig. S30. ^{13}C NMR spectrum of compound **3d**

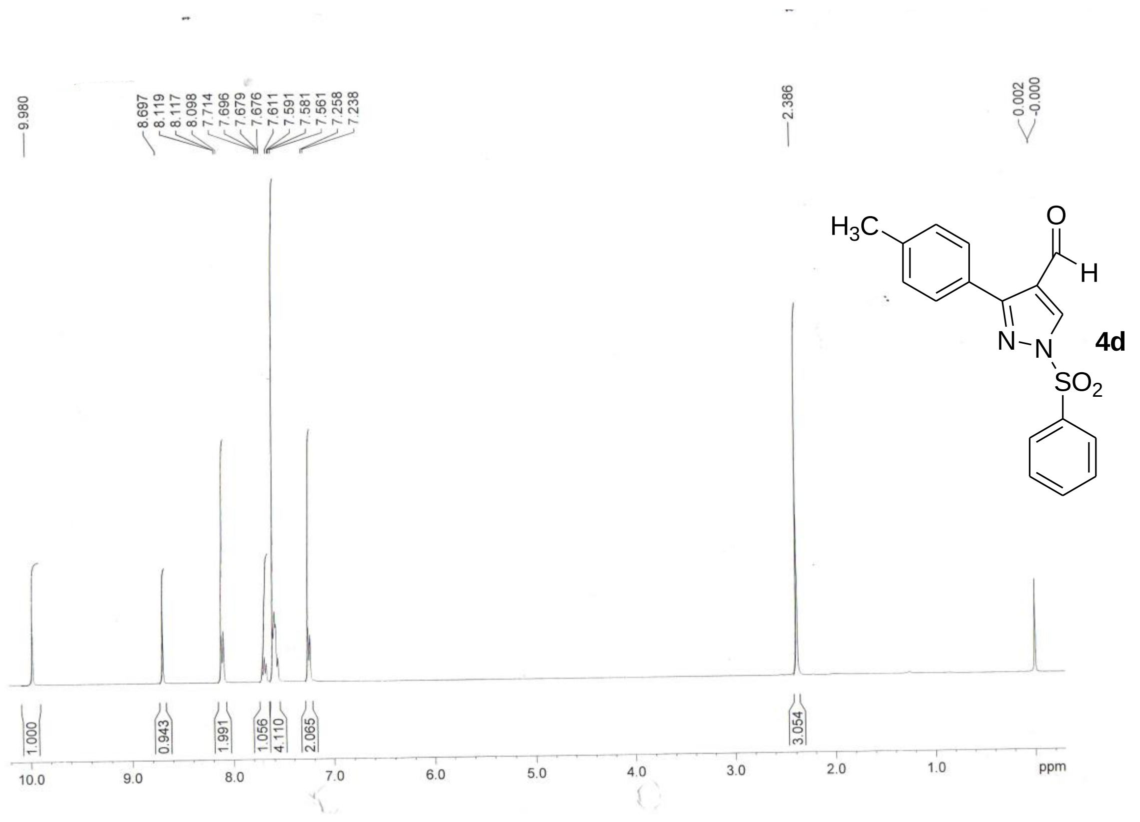

Fig. S31. ^{1}H NMR spectrum of compound **4d**

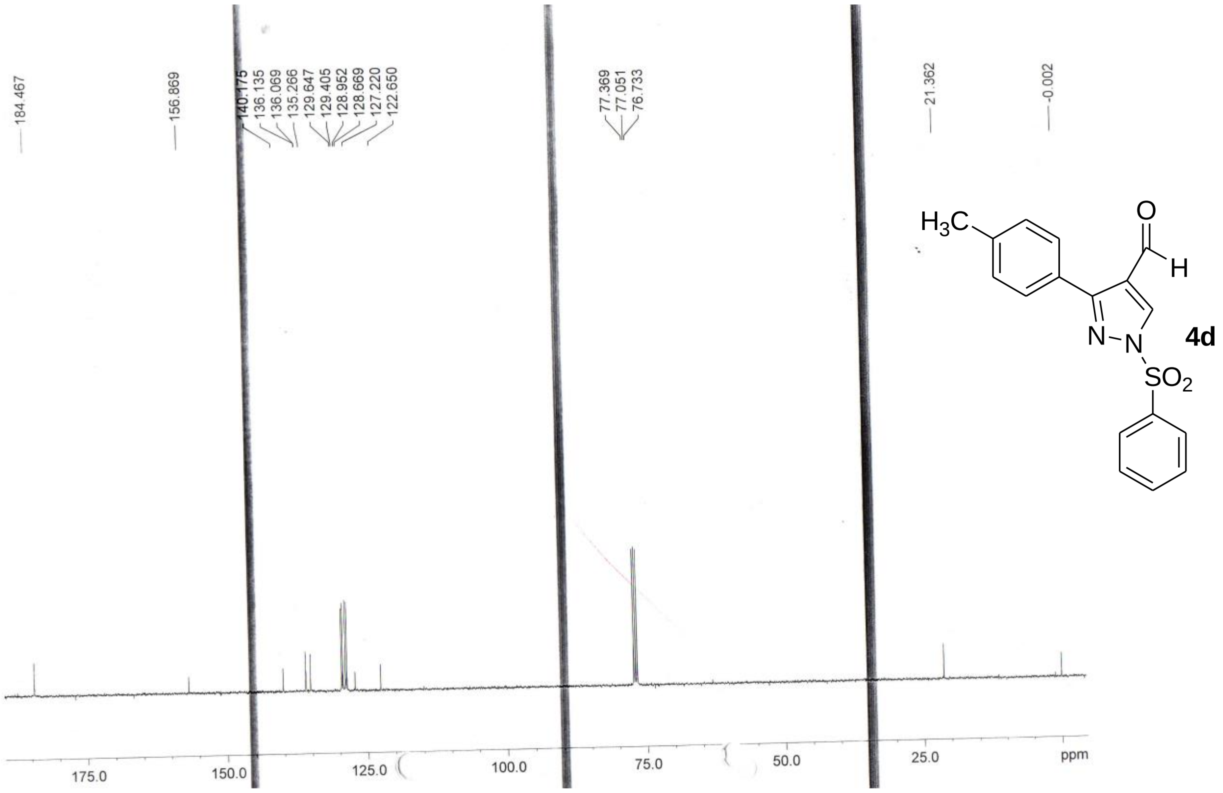

Fig. S31. ^{1}H NMR spectrum of compound **4d**
